# Deciphering the underlying genetics of galling resistance to the blueberry stem gall wasp in northern highbush blueberry

**DOI:** 10.1093/hr/uhaf197

**Published:** 2025-07-29

**Authors:** Scott J Teresi, Mélanie J A Body, Alder Fulton, Adrian E Platts, Marivi Colle, Philip D Fanning, Jacquelyn A Perkins, Rodrigo R Amadeu, Juliana Benevenuto, Patricio Munoz, Jack C Schultz, Rufus Isaacs, Patrick P Edger

**Affiliations:** Department of Horticulture, Michigan State University, East Lansing, MI 48824, USA; Genetics and Genome Sciences Program, Michigan State University, East Lansing, MI 48824, USA; Department of Horticulture, Michigan State University, East Lansing, MI 48824, USA; Department of Horticulture, Michigan State University, East Lansing, MI 48824, USA; Department of Horticulture, Michigan State University, East Lansing, MI 48824, USA; Department of Horticulture, Michigan State University, East Lansing, MI 48824, USA; Department of Entomology, Michigan State University, East Lansing, MI 48824, USA; Department of Entomology, Michigan State University, East Lansing, MI 48824, USA; Blueberry Breeding and Genomics Lab, Horticultural Sciences Department, University of Florida, Gainesville, FL 32611, USA; Blueberry Breeding and Genomics Lab, Horticultural Sciences Department, University of Florida, Gainesville, FL 32611, USA; Blueberry Breeding and Genomics Lab, Horticultural Sciences Department, University of Florida, Gainesville, FL 32611, USA; Department of Biology and Biochemistry, University of Houston, Houston, TX 77204, USA; Department of Entomology, Michigan State University, East Lansing, MI 48824, USA; Ecology, Evolution, and Behavior Program, Michigan State University, East Lansing, MI 48824, USA; Department of Horticulture, Michigan State University, East Lansing, MI 48824, USA; Genetics and Genome Sciences Program, Michigan State University, East Lansing, MI 48824, USA; Ecology, Evolution, and Behavior Program, Michigan State University, East Lansing, MI 48824, USA

## Abstract

Certain specialist herbivorous insects have evolved elegant mechanisms to manipulate the physiology of their host plants, including the ability to redirect the fate of plant cells toward the creation of a novel, tumor-like organ, called ‘galls’. While some plants have evolved resistance to gall-inducing insects, the underlying genetic mechanisms remain poorly understood. In this study, we focused on the chalcid gall-inducing wasp, *Hemadas nubilipennis* (Ormyridae) and its host plant, highbush blueberry *Vaccinium corymbosum* (Ericaceae). To identify the genetic basis of resistance to gall induction in blueberry, we developed a genetic mapping population derived from the susceptible ‘Liberty’ and resistant ‘Draper’ cultivars. We identified four quantitative trait loci (QTLs) associated with galling resistance, with candidate genes in these regions associated with plant defense, biotic stress response, and phytohormone metabolism. Furthermore, we analyzed gene expression on days one through seven post-oviposition in both susceptible and resistant genotypes, compared to controls, to identify genes and pathways that may contribute to galling resistance. Gene expression analyses, including genes within the four identified QTL regions, revealed a robust early defense response in the resistant genotype, marked by upregulation of defense, stress, and immunity genes following oviposition, ultimately leading to insect death. Conversely, the susceptible genotype exhibited a delayed and weaker response, allowing gall development and insect survival. We expect these results to serve as a resource that will enable breeding programs to employ molecular approaches for selection of resistant cultivars, while also guiding future research aimed at studying the evolution of galling resistance.

## Introduction

The constant threat of herbivory has driven the evolution of an impressive array of plant defenses, sparking an ongoing evolutionary arms race with insect herbivores [1]. With >400 000 insect species being phytophagous (plant-eating), plants have evolved diverse strategies to deter herbivory, including physical barriers, chemical deterrents, and intricate signaling pathways [2–4]. Roughly 10 000 of these insect species are considered major crop pests, highlighting the ongoing challenge for agriculture to manage herbivore damage and ensure food security [5–8].

Plants have coevolved with herbivorous insects for at least 400 million years, and these interactions form a continuum ranging from mutualism (e.g. pollinators) to antagonism (e.g. herbivores) [9, 10]. Here, we focus on antagonist relationships between insects and their host plant. During their coevolution, plants and insects have developed elegant adaptive strategies to avoid or resist each other’s defense systems. Through an ongoing evolutionary dynamic, plants have developed sophisticated systems to perceive nonself components, which then trigger their immune defenses against herbivorous insects [11–14]. In return, herbivorous insects have evolved mechanisms to counteract these defensive strategies, including the establishment of a parasitic symbiosis with their host plant by manipulating the host’s morphology and/or physiology to their own advantage [15–20].

One example of a parasitic symbiosis found in nature is gall induction by insects [21]. These insects redirect the fate of undifferentiated host plant cells toward the formation of nutrient-rich, tumor-like outgrowths of plant tissues, called galls [17]. Galling has evolved independently among multiple insect orders including Hymenoptera [22–27], and has been documented for >20 000 species [28]. Plant-manipulating insects pose a significant threat to global flora and agricultural output, impacting an estimated 15%–50% of vascular plant species worldwide. This vulnerability extends to vital crops like grape, wheat, rice, and blueberry, where these insects can lead to considerable decreases in crop yield [29–32].

Gall formation stems from a complex interaction where the insect essentially ‘hijacks’ the plant’s molecular processes. Despite this understanding, the specific chemical identity and the precise way that stimuli or effectors, originating from insect salivary secretions or oviposition fluids, alter the host plant remain undefined [33, 34]. In certain plant–galler systems, the location where a gall forms is distinct from where the insect feeds, which suggests the involvement of a chemical signal [35, 36]. Because galls grow via cell hypertrophy and tissue hyperplasia, attention has focused on plant hormones, especially growth phytohormones, such as auxins (AUXs) and/or cytokinins (CKs) [16, 37–43]. A recent study on grape phylloxera showed that this insect hijacks plant molecular pathways, usually involved in plant reproduction and maintenance of undifferentiated cells, to induce galls on grapevine leaves [17].

**Figure 1:**
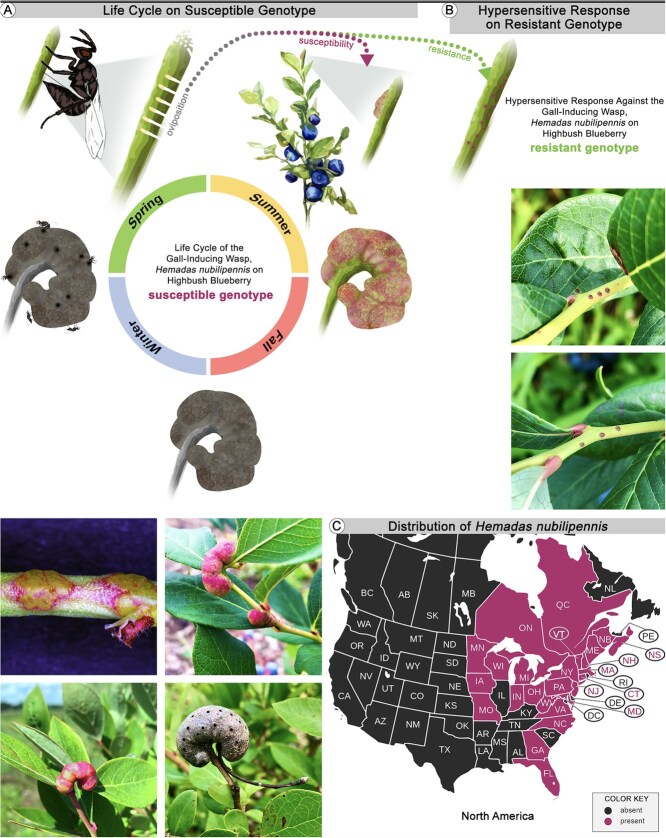
Life cycle and distribution of the gall-inducing wasp *H. nubilipennis* on the highbush blueberry *V. corymbosum*; (A) Life cycle on the susceptible genotype (‘Liberty’), (B) Hypersensitive response on the resistant genotype (‘Draper’), (C) Distribution of *H. nubilipennis* in North America [44–46].

In this study, we focused on the chalcid gall-inducing wasp, *Hemadas nubilipennis* (Hymenoptera, Ormyridae), and its host plant, the northern highbush blueberry *Vaccinium corymbosum* (Ericaceae) (Fig. 1). While some previous studies have aimed at identifying genomic loci associated with resistance to galling by other insect species in different host plant species [47–49], no resistance loci for the blueberry stem gall wasp have been reported to date. The discovery of such regions could significantly accelerate molecular breeding efforts to develop superior resistant blueberry cultivars. As the world’s leading producer of blueberries, the USA was responsible for 36% of global production, yielding 711.2 million pounds (322 595 mt) in 2023 [50]. The highbush blueberry industry generates >$4.7 billion in annual economic impact in the USA [50] (US Highbush Blueberry Council, 2025). It is estimated that the wasp currently impacts ~10% of blueberry acreage in Michigan, among the top five blueberry-producing states in the USA, with its distribution and population size steadily increasing over the past 10 years [51, 52]. The galls formed by the wasp reduce yield and complicate harvest due to their proximity to and similar appearance to blueberries [44]. The native range of this wasp extends through Central and most of Atlantic Canada, as well as most of the upper midwest and eastern seaboard of the USA (Fig. 1C) [44], and overlaps with that of the various blueberry species across this region [53].

Upon finding a suitable shoot tip, the female first taps the surface with her antennae. She then faces the apex and positions herself 5–15 mm below the tip [54, 55]. Next, she inserts her ovipositor into the shoot to deposit an egg, subsequently moving a few millimeters closer to the apex to repeat the process, forming a row of eggs [54, 55]. Immediately after laying her eggs, she ascends the shoot and repeatedly stabs the apical meristem and nearby tissues with her ovipositor until significant damage occurs [54, 55]. This ovipositor-induced stabbing halts the shoot’s growth, consequently redirecting nutrients that would normally nourish the shoot toward the developing gall tissues and larvae [54, 55]. Following oviposition, within 3–4 days, the plant tissues surrounding the deposited eggs initiate meristematic activity and begin rapid proliferation, forming thick layers of parenchymatous cells around each egg. Concurrently, scar tissues develop on the shoot’s surface, above the row of eggs. Hatching occurs after 12–14 days, at which point the larvae commence feeding on the cells lining their individual chambers within the developing gall. Continued cycles of cell division and cellular hypertrophy then drive the increase in gall size. These combined processes culminate in the development of a kidney-shaped (reniform) gall, each containing a distinct chamber for every larva [54, 55]. The wasps spend the winter as mature larvae encased within their galls, where they then undergo pupation during the spring. The adult insects, exclusively females, emerge by chewing their way out of the galls, a process that typically occurs between May and early July. On susceptible genotypes, the gall starts being visible 3–4 days post-oviposition (dpo), when the plant cells surrounding the eggs proliferate (Fig. 1A) [54, 55].

As part of this study, we identified a northern highbush blueberry cultivar, ‘Draper’, that is completely resistant to the galling wasp, and induces a hypersensitive response (HR) that kills the eggs within the first few days after oviposition (Fig. 1B). As a consequence, oviposition sites on the stem are surrounded by dead cells (Fig. 1B). To examine the genetic mechanisms underlying resistance to *H. nubilipennis* gall induction in blueberry, we first developed a genetic mapping population derived from two heterozygous parents ‘Liberty’ (susceptible genotype), and ‘Draper’ (resistant genotype). Secondly, we analyzed gene expression at 1–7 dpo in both susceptible and resistant genotypes, compared to controls, to identify differentially expressed genes (DEGs) that may contribute to wasp resistance. We hypothesized that genes involved in plant immunity, resistance, stress, and defense-related genes would be more upregulated in the resistant genotype relative to control tissues than in the susceptible genotype.

## Results

### Gene discovery via QTL analyses

**Figure 2:**
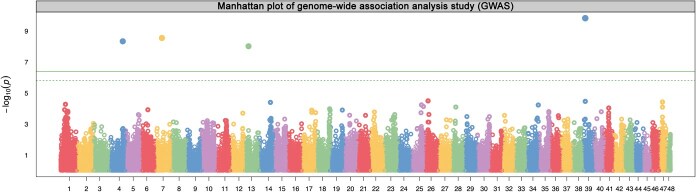
Manhattan plot showing results from a genome-wide association study (GWAS) for resistance to the gall-inducing wasp H. nubilipennis in blueberry, genomic coordinates are shown on the x-axis, and each point representing a single nucleotide polymorphism (SNP), with each chromosome being a different color, is plotted against its association p-value on the y-axis as a negative logarithm, with the most significant associations appearing as the highest points above a solid green line (Bonferroni-corrected 0.05 FDR threshold) and a dashed green line (Benjamini–Hochberg 0.05 non-FDR threshold) marking the significance thresholds.

The results were not strongly impacted by the model selected and in general suggested a similar set of four loci associated with the galling resistance phenotype (Fig. 2; Table 1; Data S1; Table S1). The allelic breakdown of the four significant SNPs is available in Fig. S1. Combining these individual sites into a single predictive model partitioned the resistant and susceptible individuals with mean groupwise scores 0.60 (resistant, *N* = 283) and −0.97 (susceptible, *N* = 204) respectively (*P* < 0.001) suggesting that the four sites jointly capture 70.2% of the genetic contribution to the gall resistance phenotype. We identified four QTLs across chromosomes chromosomes 4, 7, 13, and 39 that are associated with resistance using the BLINK (Bayesian-information and Linkage-disequilibrium Iteratively Nested Keyway [84]) model. The QTL analysis returned 56 blueberry genes (Table S1; Data S1) lying within 100 kb of the four FDR-significant marker SNPs. A recent study revealed that the highbush blueberry genome consists of 10 683 haplotype blocks with an average size of ~61 kb [85]. Thus, our approach of expanding our analysis to 100 kb on either side of the marker is conservative and intended to retain a broader set of genes for downstream analyses and filtering. Of the 56 blueberry genes, 42 were expressed in at least one of our galling stage libraries and 35 were identified as differentially expressed in at least one comparison (e.g. 1 dpo Control vs Treatment Draper) (Table S1; Data S1).

**Table 1:** Genomic regions associated with resistance to stem gall wasp resistance

**Chromosome**	**Position**	***P*-value**	**Effect**	***P*-value**	**Effect**	***P*-value**	**Effect**	***P*-value**	**Effect**	**Effect**
		**BLINK**	**BLINK**	**FarmCPU**	**CFrmCPU**	**GLM**	**GLM**	**MLM**	**MLM**	**GEMMA**
VaccDscaff39	5 172 680	1.49E−10		7.08E−10	−0.1320	5.54E−07	−0.1713	2.43E−06	−0.1675	0.09/0.09 (0.03)
VaccDscaff7	15 315 390	2.82E−09		8.44E−09	0.1717	4.20E−08	0.2376	4.85E−07	0.2274	0.005/− (0.001)
VaccDscaff4	36 106 086	4.60E−09		7.80E−08	0.1344	5.61E−07	0.1851	1.56E−05 (•)	0.1663	0.311/0.578 (0.13)
VaccDscaff13	9 802 715	9.55E−09		4.43E−06 (•)	−0.1193	8.50E−07	−0.1877	1.20E−05 (•)	−0.1753	0.01/0.015 (0.003)

Raw *P*-value (FDR significance limit 3.0E−06, values marked with (•) exceed this threshold) and effect sizes for variants at the four loci consistently suggested by each of the association models. GEMMA, a Bayesian Sparse Linear Mixed Model, was used for analyzing binary trait data in GWAS, to provide gamma for blsmm model Probit/Linear (sparse effect size for probit model).

Here, we report the gene expression of the top five selected QTL-residing genes and their known functions in other systems. The expression values mentioned hereafter refer to Log2FC values between oviposition and control stem tissues. We consider a change as significant when the Log2FC value reaches the threshold of −0.5 for downregulated genes (or a fold-change of 0.560 on a linear scale) and 0.5 for upregulated genes (or a fold-change of 1.625 on a linear scale). Taken together, these results provide a range of promising candidate genes for further investigation in northern highbush blueberry.

All of the candidate genes identified within QTLs are presented in Table S1 and Data S1. These candidates include putative homologs to, on *Chromosome 13*, *KCS5*, and AT5G16200 (50S ribosomal protein-like protein), on *chromosome 39*, *SCL14*, *RLP7*, and AT5G37540 (Eukaryotic aspartyl protease family protein). The top five resistance candidate genes are all involved in resistance against pathogens and insects. Additionally, the upregulation of two genes, *ALDH12A1* on chromosome 4 and *MEE31*/*PMI1* on chromosome 13, both of which are involved in the first line of defense against wound- and pathogen-induced ROS [43], suggests the presence of damaging oxidative stress in the resistant genotype upon oviposition. Moreover, this Genome-Wide Association (GWA) analysis also identified a gene within one QTL that could, at least partially, explain the parasitism success in the susceptible genotype. *SOD3* (chromosome 13) was upregulated on 4–5 dpo in the susceptible parent, when cells surrounding the eggs begin proliferating and differentiating (Days 3–4). Interestingly, transcripts of a gene with high sequence similarity to SOD3 were previously found in the venom glands of parasitoid wasps that inject venom into the host along with their eggs to inhibit the host’s immune system and ensure successful parasitism [86]. In the susceptible genotype, the induction of this plant gene by *H. nubilipennis* could impede the host plant’s immune response during the oviposition similar to the parasitoid wasp. In the resistant genotype, this gene expression remained unchanged when compared with controls.

### Overall gene expression patterns

**Figure 3:**
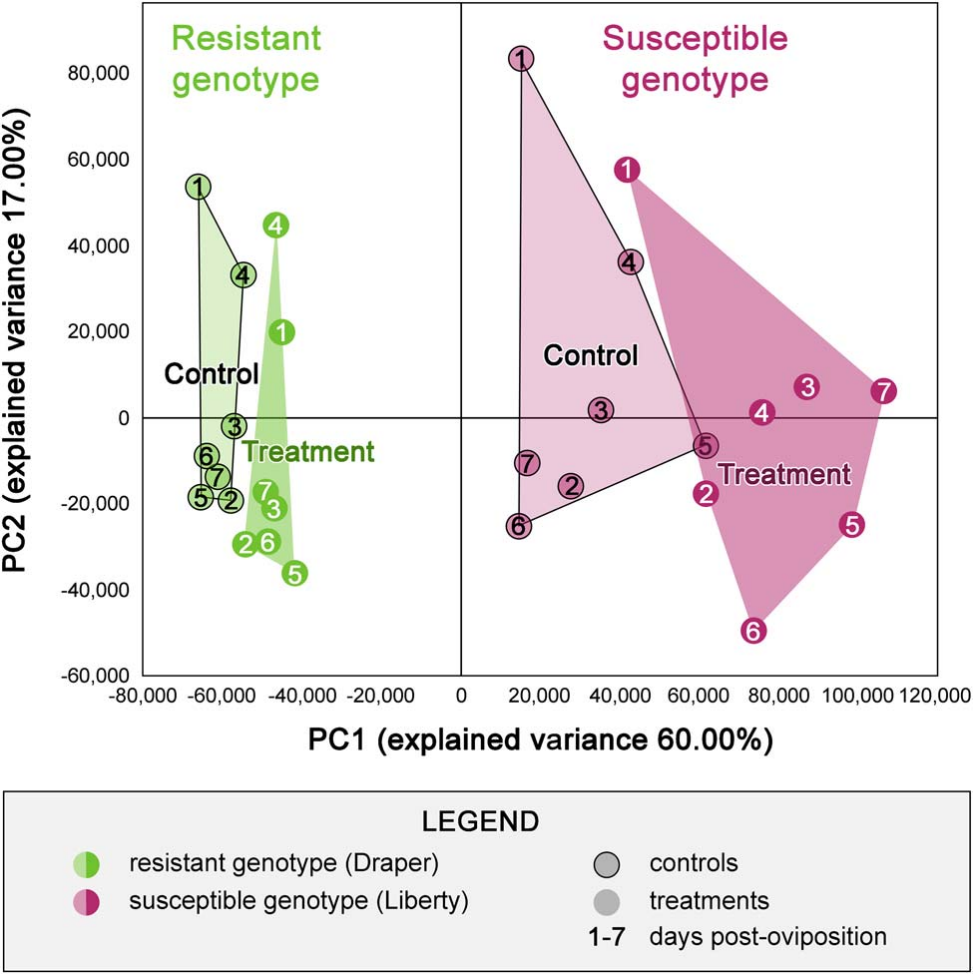
Principal component (PC) analysis of all the genes of control and treated samples from Day 1 to 7 post-oviposition in both susceptible and resistant genotypes to the blueberry stem gall wasp.

To identify the genes or pathways activated during the resistance process against *H. nubilipennis* in highbush blueberry, our RNA-seq experiment included stem tissues that were exposed to the wasp oviposition (‘treatment’ hereafter) and stem tissues that were damaged with a needle to mimic the female’s stabbing behavior during oviposition (‘control’ hereafter), during the first 7 dpo (1–7 dpo), in both resistant (‘Draper’) and susceptible (‘Liberty’) genotypes. Principal component analysis (PCA) revealed a clear separation between the genotypes on the first principal component with an explained variance of 60.00% (Fig. 3).

Analysis of the PCA plot revealed that time points 1 and 4 in the resistant genotype exhibited distinct clustering compared to other time points, suggesting unique transcriptional dynamics at these stages. While pattern in the expression of genes within the identified QTLs did not fully explain this divergence, further investigation uncovered a single, previously uncharacterized gene (VaccDscaff39-processed-gene-52.3) within these QTL regions. This gene, lacking sequence similarity to any known genes, displayed a unique expression pattern, being upregulated in the resistant ‘Liberty’ genotype and downregulated in the susceptible ‘Draper’ genotype at these specific time points. This intriguing finding warrants further functional characterization to elucidate its role in gall resistance.

### Global patterns of differentially expressed genes

We identified DEGs that were significantly different between each treatment and control across each day post-oviposition for the resistant and susceptible genotypes. We identified a total of 740 unique blueberry genes as DEGs, and identified putative *Arabidopsis* homologs for roughly 41% of these genes (301 genes total, and an overall *Arabidopsis* homolog identification rate of 81%). The overall number and pattern of DEGs following wasp oviposition was different between the susceptible and resistant genotypes, with the resistant genotype possessing the most DEGs overall (Fig. 4A). We analyzed the DEGs in three contexts. First, we compared treatment versus control (resistant) and treatment versus control (susceptible) to control for the response to oviposition. Secondly, we examined DEGs unique to each comparison to identify differences between susceptible and resistant genotypes. Third, we examined shared DEGs between the comparisons to better understand common responses in both genotypes to wasp infestation.

**Figure 4:**
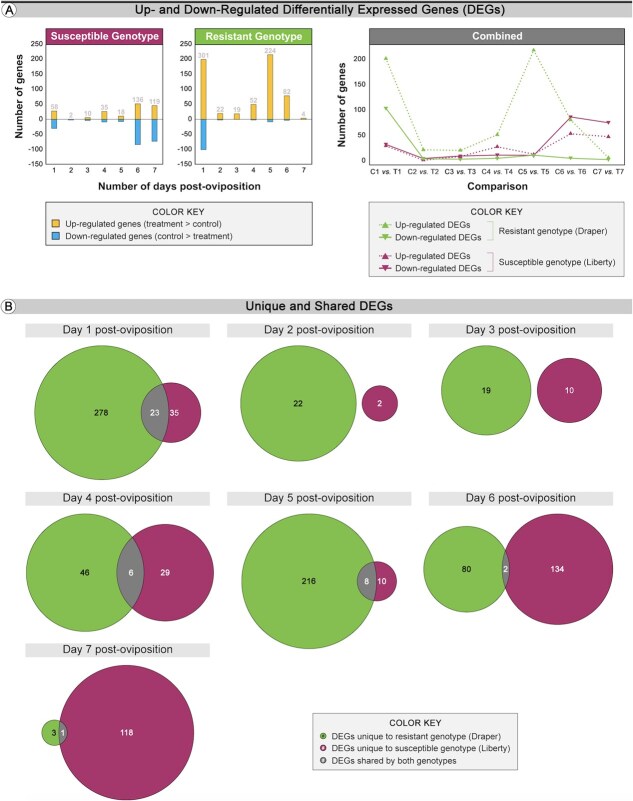
DEGs between resistant and susceptible highbush blueberry in response to blueberry stem gall wasp; (A) Number of DEGs that are up- and downregulated in susceptible and resistant genotypes between treated and control stem tissues (edgeR analysis) (see Table S2 for all DEG details); (B) Venn diagrams. Each circle displays the number of DEGs unique to either the resistant or susceptible genotype, as well as the number of DEGs shared by both genotypes, (C) Selected significantly overrepresented (classic Fisher test) GO categories for all DEGs unique to either the susceptible or resistant genotype with the size of each shape determined using the *P*-values from the TopGO analysis (see Table S3 for all TopGO analysis results).

**Figure 4:**
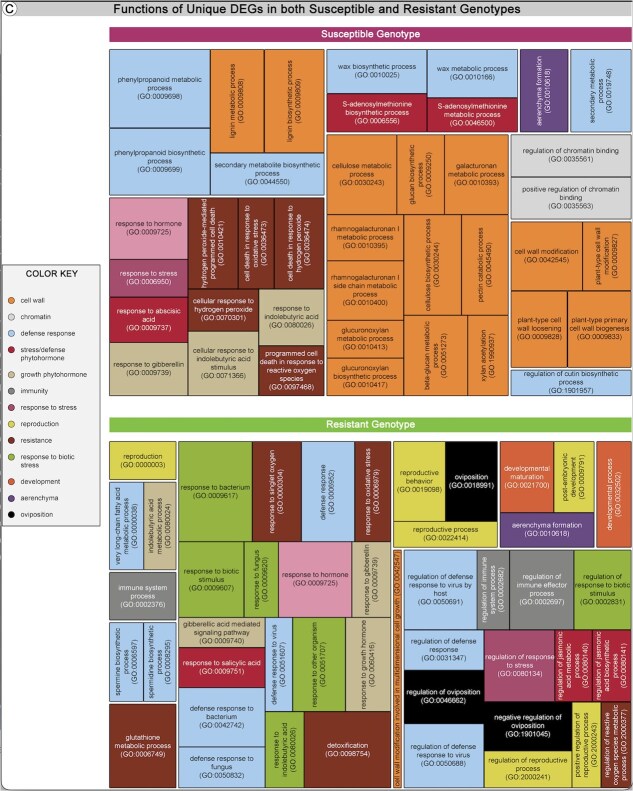
Continued.

We found five times more DEGs in the resistant genotype (301 total DEGs) than in the susceptible genotype (58 total DEGs) on 1 dpo, suggesting a stronger response to oviposition in the resistant genotype early on with 200 upregulated DEGs (second highest) and 101 downregulated DEGs (highest) (Fig. 4A). On Days 2 and 3, the number of DEGs was minimal (2–22 DEGs) for all conditions. On Day 4, the number of upregulated DEGs was higher than on Day 3 for both genotypes (50 DEGs for the resistant genotype and 26 DEGs for the susceptible genotype). On Day 5, while the number of up- and downregulated DEGs (11 and 7 DEGs, respectively) was lower than on Day 4 in the susceptible genotype, the number of upregulated DEGs was at its maximum in the resistant genotype (216 DEGs), although the number of downregulated DEGs remained low (8 DEGs). On Days 6 and 7, the number of up- and downregulated genes (52 and 84 DEGs, respectively, on Day 6; 46 and 73 DEGs, respectively, on Day 7) was at their highest in the susceptible genotype. In contrast, in the resistant genotype, the number of up- and downregulated genes (79 and 3 DEGs, respectively, on Day 6; 4 and 0 DEG, respectively, on Day 7) decreased drastically on Day 6 and then Day 7 post-oviposition.

**Figure 5:**
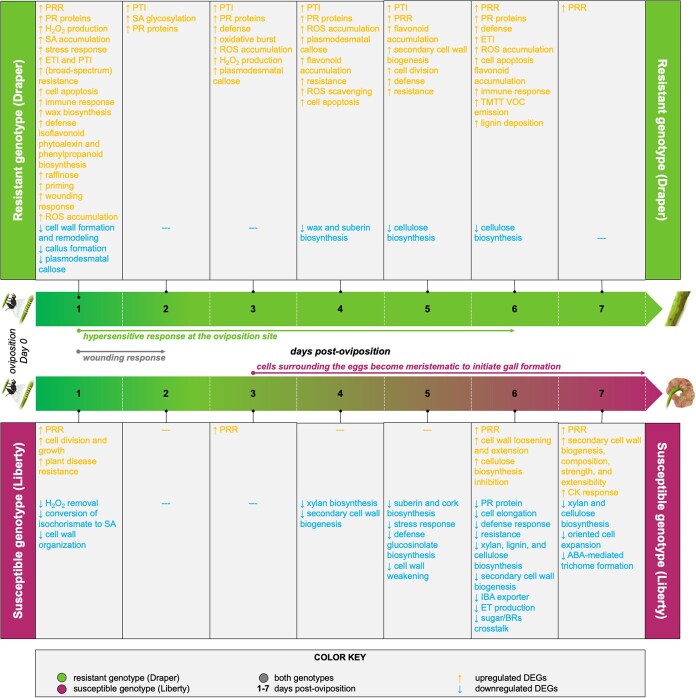
Functions of DEGs in resistant and susceptible genotypes in response to blueberry stem gall wasp infestation. DEG functions across the first 7 dpo in both resistant (‘Draper’) and susceptible (‘Liberty’) genotypes.

We also investigated DEGs unique to either genotype (Table S2). A summary of unique DEGs, and their predicted functions, identified in the susceptible and resistant genotypes is presented in Fig. 5. Detailed information on all key DEGs across each day are further discussed in Data S2.

DEGs shared by both the resistant and susceptible genotypes were also investigated to determine their potential roles in blueberry–wasp interactions (Fig. 4B; Table S2). On Day 1, we found 23 DEGs, 8 of which were assigned putative *Arabidopsis* homologs, that were shared between resistant and susceptible genotypes. Expression of three genes (*RLP1*, Log2FC = 2.94 (susceptible) and 4.83 (resistant); *PBL36*, Log2FC = 2.19 (susceptible) and 2.56 (resistant); *NAC082* / *VNI1*, Log2FC = 7.58 (susceptible) and 6.07 (resistant); see Data S2 for detailed information on those genes) have previously been associated with disease resistance [87–89]. Expression of all three genes was upregulated in both genotypes on Day 1 after oviposition. We did not identify any shared DEGs for Days 2–3. On Day 4 and beyond, we identified DEGs but relatively few of them had identifiable homologs, and none of the homologs had putative functions relevant to gall induction or plant resistance.

We ran GO enrichment analysis to summarize the functions of unique DEGs (Fig. 4C; Table S3). In the susceptible genotype, the up- and downregulated DEGs belonged to gene ontologies related to cell wall, chromatin, defense response, stress/defense phytohormone, growth phytohormone, response to stress, resistance, development, and aerenchyma, among other functions (Fig. 4C top panel; Table S3). In the resistant genotype, the up- and downregulated DEGs were related to GO involved in cell wall, defense response, stress/defense phytohormone, immunity, response to stress, reproduction, resistance, response to biotic stress, development, aerenchyma, and oviposition (Fig. 4C bottom panel; Table S3).

### Gene Coexpression network analysis of differentially expressed genes

**Figure 6:**
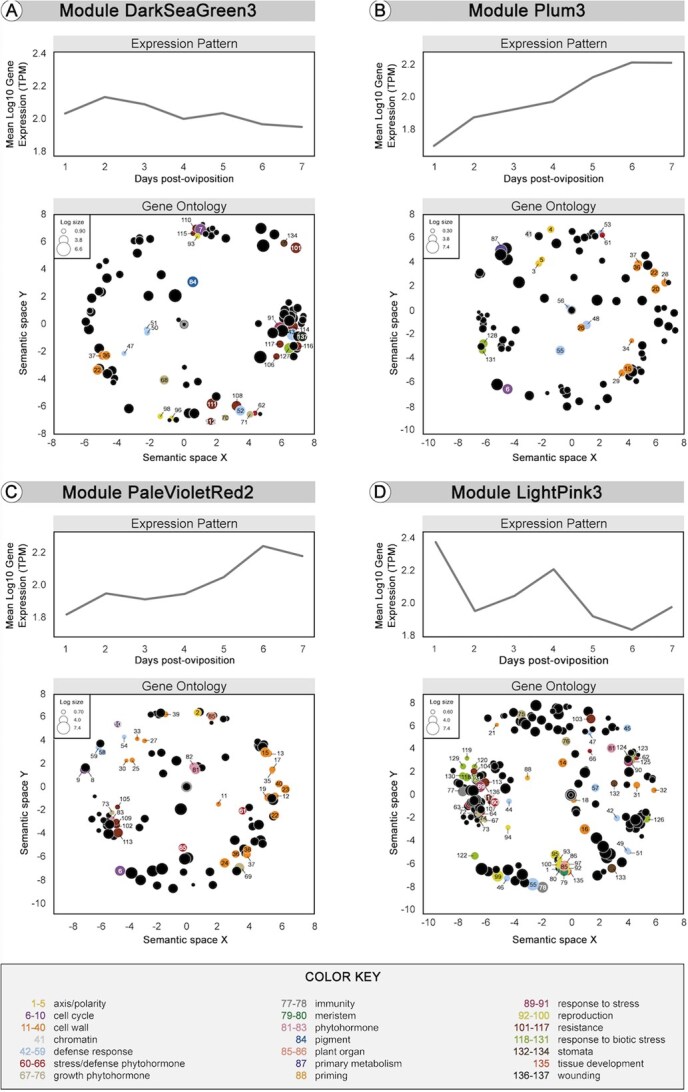
Coexpression network modules for gene responses to infestation of highbush blueberry by blueberry stem gall wasp with expression pattern (top panels) and GO terms (bottom panels) shown for each of the four main coexpression network modules: (A) DarkSeaGreen3 module, (B) Pum3 module, (C) PaleVioletRed2 module, and (D) LightPink3 module.

Gene coexpression modules that are enriched with DEGs were used to identify pathways and gene functions overrepresented during gall development. First, a gene coexpression network was constructed from the transcriptome data, which identified 89 gene modules. The average module size consisted of 1051 genes, the maximum size was 14 499 genes, and the minimum size was 166 genes. Gene coexpression network analysis revealed four main modules that consisted of >5% DEGs: DarkSeaGreen3, Plum3, PaleVioletRed2, and LightPink3 (Fig. 6; Tables S4 and S5 for the details of GO categories). These are described in more detail below.

### DarkSeaGreen3 module

Module DarkSeaGreen3 contained 188 genes with an expression pattern showing a peak on Days 2–3 dpo (Fig. 6A; Tables S4 and S5 for the details of GO categories). This module contained 23.40% (19.68% were upregulated on 1 dpo) and 19.15% (all were upregulated on 5 dpo) of the total DEGs in the resistant genotype. Additionally, it contained 6.38% (5.32% were upregulated) of the total DEGs on Day 1 dpo in the susceptible genotype. Gene functions of the DEGs in the DarkSeaGreen3 module were enriched with the following categories; cell wall, defense response, growth phytohormone, pigments, response to stress, reproduction, resistance, response to biotic stress, stomata, and wounding.

### Plum3 module

Module Plum3 contained 324 genes with an expression pattern displaying an increase between Day 1 and 6 dpo and a plateau on 6–7 dpo (Fig. 6B; Tables S4 and S5 for the details of GO categories). This module contained 10.19% (all were upregulated) and 5.86% of the total DEGs (all were upregulated) on 5 and 6 dpo, respectively, in the resistant genotype. Additionally, it contained 7.10% (6.48% were downregulated) and 6.79% (all were downregulated) of the total DEGs on Days 6 and 7 dpo, respectively, in the susceptible genotype. The DEGs in the Plum3 module were involved in axis/polarity, cell cycle, cell wall, chromatin, defense response, stress/defense phytohormone, primary metabolism, and response to biotic stress.

### PaleVioletRed2 module

Module PaleVioletRed2 contained 274 genes with an expression pattern presenting similarities with the module Plum3 (Fig. 6C; Tables S4 and S5 for details of GO categories). In this module, the expression increased from 1 to 6 dpo, with a slight depression/dip on 3–4 dpo, and a slight decrease on 7 dpo. This module contained 10.95% (10.58% were downregulated) and 9.85% (all were downregulated) of the total DEGs on 6 and 7 dpo, respectively, in the susceptible genotype. The DEGs in the PaleVioletRed2 module were involved in axis/polarity, cell cycle, cell wall, defense response, stress/defense phytohormone, growth hormone, phytohormone, plant organ, and resistance.

### LightPink3 module

Module LightPink3 contained 251 genes with an expression pattern displaying highest expression on Days 1 and 4 (Fig. 6D; Tables S4 and S5 for the details of GO categories). This module contained 5.58% (5.18% were upregulated) of the total DEGs on 1 dpo in the resistant genotype. The DEGs in the LightPink3 module were involved in axis/polarity, cell wall, defense response, stress/defense phytohormone, immunity, meristem, phytohormone, plant organ, priming, reproduction, resistance, response to biotic stress, stomata, tissue development, and wounding.

## Discussion

This study investigated genetic and transcriptional differences between resistant and susceptible highbush blueberry genotypes against the gall-inducing wasp, *H. nubilipennis*, examining marker–phenotype associations and gene expression patterns to discern differences between resistant and susceptible genotypes. The resistant genotype exhibited a robust early response to oviposition, marked by upregulation of genes associated with defense, stress response, and immunity. Conversely, the susceptible genotype showed delayed and weaker responses, with increased expression during gall initiation. Notably, the hypersensitive response in the resistant genotype resulted in insect death shortly after oviposition, contrasting with gall development and insect survival in the susceptible genotype. Receptor-mediated recognition of oviposition involves various receptor-like proteins and kinases, particularly prevalent among DEGs in the resistant genotype, indicating a complex defense cascade activation.

The resistant genotype exhibited components of both Pattern-Triggered Immunity (PTI) and Effector-Triggered Immunity (ETI) as early as 1 dpo, whereas these were lacking or limited in the susceptible genotype. PTI, initiated by pattern recognition receptors (PRRs), offers moderate resistance against a broad spectrum of pathogens [90, 91], while ETI provides strong and localized resistance to specific pathogens carrying avirulence (*Avr*) genes [92, 93]. These two immune branches synergistically enhance each other, with ETI boosting PTI signaling components [90, 92]. The resistant genotype’s reaction included components of PRR induction, ROS production, callose deposition, hypersensitive response leading to cell death, and the release of volatile organic compounds (VOCs) that may attract natural pest enemies. Conversely, the susceptible genotype exhibited limited activation of these genes, and failed to mount a hypersensitive response. Furthermore, only the resistant genotype displayed Systemic Acquired Resistance (SAR), characterized by the induction of pathogenesis-related proteins (PRs) upon oviposition. Two uncharacterized PR thaumatin superfamily proteins were identified, with differential expression patterns between genotypes, suggesting their involvement in blueberry resistance against this pest.

Plant cell wall-mediated resistance was evident in the upregulation of genes such as LAC14 involved in lignin deposition and strengthening cell walls in the resistant genotype [94–96]. Additionally, upregulation of genes for callose deposition along plasmodesmata (PD) was observed, which would potentially limit gall initiation by impeding cell-to-cell communication. In contrast, the susceptible genotype exhibited downregulation of genes associated with cell wall fortification, potentially increasing susceptibility to the wasp.

This study also identified several key pathways and genes involved in plant defense and growth hormone biosynthesis in response to oviposition by *H. nubilipennis*. In the susceptible genotype, genes associated with stress/defense phytohormone biosynthesis, such as UGT74F1 and SAM2, showed expression patterns suggesting an initial resistance response followed by decreased resistance as the larvae hatched from the laid eggs. However, in the resistant genotype, these genes exhibited enhanced and prolonged resistance. UGT74F1, involved in salicylic acid metabolism [97], was upregulated for multiple days, potentially contributing to sustained resistance. SAM2, involved in ethylene (ET) production [98, 99], showed increased expression on Day 6, enhancing ET emission and resistance. Additionally, genes involved in growth hormone biosynthesis, such as IBR10, were upregulated in both genotypes, suggesting roles in either gall initiation or wound healing after oviposition.

Further analysis revealed genes within QTLs involved with cytokinin and brassinosteroid biosynthesis, indicating potential roles in cell division, growth, and resistance. Notably, the resistant genotype exhibited upregulation of genes associated with direct and indirect defense mechanisms, including pathways for phenylpropanoids, terpenoids, and lipoxygenases. These pathways likely contribute to the production of chemical defenses and the emission of VOCs that may also repel harmful insects and attract natural enemies of the attacker [43, 100]. The GWA analysis also identified potential resistance genes, such as a homolog to AT1G66880 (a member of the LRK10L subfamily of receptor-like protein kinases). This protein has previously been shown to be involved in plant defense against pathogens, and its expression is also observed during plant embryo development [101–103]. The resistant genotype demonstrated a stronger and longer lasting resistance response to wasp oviposition compared to the susceptible genotype. These findings provide insights into the molecular mechanisms underlying blueberry resistance to gall-inducing wasps and identified candidate genes for further investigation.

Overall, the study underscores the intricate interplay of molecular pathways governing resistance of plants to gall-inducing wasps, with implications for future research into plant–insect interactions and breeding strategies for pest resistance. Our results show the timeline of plant responses post-oviposition, with distinct waves of reaction observed in both the susceptible and resistant genotypes. These responses are characterized by changes in gene expression related to defense, stress, growth, and reproduction, highlighting the dynamic and complex nature of plant–insect interactions. To clarify the specific functions of individual candidate genes and validate the SNP markers associated with blueberry resistance to these pests, further functional analyses with a narrower focus than the GO and pathway enrichment analyses presented here are necessary as part of future studies. A more comprehensive understanding of resistance mechanisms could inform targeted breeding efforts to enhance blueberry resilience against insect pests and pathogens, contributing to more sustainable crop protection strategies.

## Material and methods

### Plant material and phenotypic data collection

Two genotypes, one susceptible and one completely resistant of northern highbush blueberry (*V. corymbosum*), were identified after screening a diversity panel of cultivars using no-choice assays [56] with *H. nubilipennis.* Wounding at oviposition sites in resistant genotypes and early signs of gall development in susceptible genotypes were observed within a week and were tracked over 3 months to verify susceptibility. Once two cultivars, Draper (resistant) and Liberty (susceptible), were identified, individuals of various ages (3, 2, 1 year, and 6 months old) were screened for resistance. We observed that resistance was consistent across all ages (i.e. not age-related resistance). In 2016, a hybrid F1 population derived from reciprocal crosses of Draper and Liberty was generated at Michigan State University. All 530 progeny in this the population, at minimum 9 months old, grown in individual pots grown in a peat moss potting soil mixture, were screened for resistance in the Horticulture Teaching and Research Center greenhouses at Michigan State University in spring 2017. Each plant was individually bagged with a minimum of 20 females (postcopulation). We observed roughly half of the individuals in the population exhibiting resistance to the galling insect. The population was screened again in the field in 2018 using no-choice assays to verify findings. Only wounding at oviposition sites was observed in the resistant genotypes across both years, while full gall development was observed in susceptible individuals. Individuals were scored as either resistant (no galls formed) or susceptible (galls formed).

### Association mapping

Quantitative trait loci (QTLs) were identified through association mapping of polymorphic sites relative to the gall-resistance phenotype in a population of 530 individuals from the ‘Draper’ × ‘Liberty’ population. Leaf samples from each individual were collected for DNA extraction and genotyping at RAPiD Genomics (Gainesville, FL, USA). A capture-seq genotyping approach [57] was performed with 10 000 biotinylated probes of 120-mer. Target-enriched libraries were sequenced to a target depth of 60× with 2× 150 bp Illumina reads [58, 59]. The reads were trimmed (Cutadapt v3.3) [60] for adapters and low-quality (*q* < 20) 3′ sequences and aligned with Burrows–Wheeler Aligner (BWA v0.7.17) [61] in paired-end mem mode to the Draper reference assembly [62]. Sequence Alignment Map (SAM) formatted reads were converted to a Binary Alignment Map (BAM) using Samtools (Samtools v1.13) [63], sorted and readgroups added by Genome Analysis Toolkit (GATK v4.2.0) [64]. After removing samples with low average sequence depth (<10×), 488 samples were used for association with the phenotype, of which 205 individuals were phenotyped as resistant to gall formation by the wasp. Given the potential for unusual allele frequencies to arise from aberrant recombination between the four haplotypes/subgenomes, autopolyploid inheritance [65,], and the reduced power of tools such as R/qtl in the context of binary categorical traits, we opted for association mapping essentially as described in [66] in which polymorphic sites were initially identified on a per sample basis with GATK haplotypecaller in Genomic Variant Call Format (GVCF) mode, combined hierarchically and genotyped for population single nucleotide polymorphisms (SNPs) with GATK CombineGVCFs and GATK GenotypeGVCFs. The raw VCF file was further processed using VCFtools (v0.1.15) [67] to filter for biallelic SNPs with a minimum mean depth of 20, a minimum quality score (Q) of 30, and a minor allele frequency >0.05. The filtered VCF file was then analyzed in R using the GAPIT package (v3.0) [68] with the following statistical models: GLM, MLM, FarmCPU, and BLINK. The Benjamini–Hochberg procedure was used to correct for multiple testing [68–70]. GEMMA [71] was used to generate a BSLMM and to estimate the associated effect size.

### Gall induction for RNA-seq

One-year-old individuals of northern highbush blueberry cultivars, ‘Liberty’ (susceptible genotype) and ‘Draper’ (resistant genotype), were planted in individual pots grown in a soil and peat moss potting soil mixture. The plants were grown in growth chambers under the following conditions: temperature of 18°C lights on/15°C lights off, 60% relative humidity, and 16:8 (L:D) photoperiod. To minimize variation, all plants were of identical age, propagated from microshoots derived from a common mother plant, and grown in large walk-in growth chambers. Sampling from both treated and control plants was performed at a consistent time of day, specifically between 4 and 5 h Zeitgeber Time. Plants were randomized daily within the growth chambers to mitigate any potential within-chamber environmental effects. This rigorous control strategy was designed to ensure that gall induction exerted a dominant effect on the stem (target organ), aimed at making any confounding within-chamber variation.

On the day of the experiment, each blueberry plant was placed in a cage and one mated female *H. nubilipennis* wasp was added to each cage. We developed a video tracking platform using Raspberry Pi and OpenCV v3.1.0 [72] to track the insect in real time within a cage containing the host plant. This allowed us to observe and record the oviposition process, to identify the precise location and time of oviposition by a single wasp. Both Liberty and Draper control plants were pierced with a syringe needle across young stem tissue to simulate tissue damage caused by oviposition. From 1 to 7 dpo, sections of stem tissues (exposed or not to oviposition) from the resistant and susceptible genotypes (three biological replicates per time point per genotype) were harvested every 24 h. Samples were immediately flash frozen in liquid nitrogen, and stored at −80°C until dissection and RNA extraction.

### RNA-seq experiment

*RNA Extraction* – Total RNA was extracted and DNase1-treated, on column, using the RNeasy Mini Kit (Qiagen, Germantown, MD, USA). The resulting RNA was further purified and concentrated with the RNeasy MinElute Cleanup Kit (Qiagen) and eluted with water. The quality of the resulting RNA was assessed using the Agilent 2100 BioAnalyzer (Agilent, Santa Clara, CA, USA), and all RNA integrity number values were found to be >8.

#### Illumina library and construction

Illumina libraries (three biological replicates for each of the seven gall developmental stages and respective control from both genotypes, for a total of 84 libraries) were constructed using the RNA TruSeq Kit (Illumina, Inc., San Diego, CA, USA), barcoded, enriching for mRNA by utilizing poly(A) selection, and sequenced paired-end with 150-bp reads on the Illumina HiSeq-4000 platform at Michigan State University Research Technology Support Facility Genomics Core.

#### Illumina read processing and expression quantification

The northern highbush blueberry genome [62] was indexed using STAR v2.6.1 [73]. Reads were trimmed using Trimmomatic v0.38 [74] and the Illumina TruSeq3-PE adapter sequence. Reads were then aligned to the genome using STAR and multimapping reads were removed from further analysis. Aligned reads were sorted using SAMTools v1.9 [75]. Counts of reads were generated using HTSeq v0.12.4 [76].

### Differentially expressed genes analysis

DEGs were identified using edgeR v3.30.3 [77] and R v4.0.2 [69]. Comparisons were made between samples of similar time points, e.g. 1 dpo susceptible genotype versus 1 dpo resistant genotype. A *P*-value cut-off of 0.05 and a Benjamini–Hochberg multiple-test false discovery rate (FDR) correction was applied to each comparison individually.

### Gene homolog prediction

In order to leverage known gene functions in *Arabidopsis thaliana*, we predicted putative *A. thaliana*–*V. corymbosum* homologs using a combination of synteny-based approaches using SynMap [78] and reciprocal BLASTp [79] analyses (protein databases). Homolog pairs with E-values ≥0.05 were excluded from both datasets. In the event that SynMap or BLASTp returned multiple *Arabidopsis* genes for a single blueberry gene, the *Arabidopsis* homolog with the lowest E-value score was kept. In the event that a blueberry gene had a homolog identified through BLASTp and SynMap, we gave priority to the SynMap synteny results, preferentially keeping that homolog pair.

### Gene coexpression network construction

We constructed gene coexpression networks with the aforementioned transcriptome data using R v4.0.0 [69] and WGCNA v1.69 [80], resulting in a network of 93 584 blueberry genes spread across 89 modules. Prior to network construction, 34 976 genes were removed due to a paucity of expression data as consistent with the guidelines of WGCNA [80].

### Gene ontology analysis

Gene Ontology (GO) enrichment analyses were performed using our *Arabidopsis* homolog predictions using R v3.6.2 [69] with TopGO v2.38.1 [81]. GO terms for the *Arabidopsis* homologs were acquired from TAIR and the GO SLIM dataset [82]. Each coexpression module was evaluated for over/underrepresentation of GO terms in the biological process (BP) sub-ontology. GO terms with a *P*-value ≥0.05 were excluded from the final results for both analyses.

Fold-change between treatment (stems subjected to oviposition) and their respective ungalled control stems was calculated using the following formula for each gene by subtracting the base-2 logarithm of the fragments per kilobase million (FPKM) value of treatments from the base-2 logarithm of the FPKM value of ungalled stems, for each day post-oviposition (1–7 dpo), and both genotypes (susceptible ‘Liberty’ and resistant ‘Draper’): [Log2FC = Log2 (averaged treatment tech rep FPKM + 1) − Log2 (averaged control tech rep FPKM + 1)]. The GO enrichment analyses were limited to genes with a Log2 fold-change (Log2FC) threshold of −0.5 for downregulated genes and 0.5 for upregulated genes.

### Principal component analysis

The principal component analysis (PCA) analysis was generated using WebMeV (Multiple Experiment Viewer) [83]. For this analysis, we used our RNA-seq expression table (FPKM) after calculating the average of the three biological replicates for each time point and genotype.

### Identification of differentially expressed candidate genes within QTL regions

Among the 56 blueberry genes present within 100 kb of the four markers, we used gene functions and gene expression patterns in both genotypes to determine the best candidates to explain blueberry resistance to the gall-inducing wasp.

## Supplementary Material

Web_Material_uhaf197

## Data Availability

Genomic target sequence capture and RNA-seq data that were generated for this study are available at NCBI SRA under BioProject PRJNA842728. All version-controlled code related to the analyses can be found at https://github.com/sjteresi/Blueberry_RNA_Seq_Expression_Analysis and https://github.com/sjteresi/Blueberry_Network_Rewiring. Annotated scripts and READMEs containing additional information can be found in the GitHub repositories.

## References

[1] Ehrlich PR, Raven PH. Butterflies and plants: a study in coevolution. Evolution. 1964;18:586–608

[2] Fürstenberg-Hägg J, Zagrobelny M, Bak S. Plant defense against insect herbivores. Int J Mol Sci. 2013;14:10242–97 23681010 10.3390/ijms140510242 PMC3676838

[3] Mithöfer A, Boland W. Plant defense against herbivores: chemical aspects. Annu Rev Plant Biol. 2012;63:431–50 22404468 10.1146/annurev-arplant-042110-103854

[4] Erb M . Plant defenses against herbivory: closing the fitness gap. Trends Plant Sci. 2017;23:187–94 29223923 10.1016/j.tplants.2017.11.005

[5] Schoonhoven LM, Van Loon B, van Loon JJA. et al. Insect-Plant Biology. New York, USA: Oxford University Press; 2005

[6] Pieterse CMJ, Dicke M. Plant interactions with microbes and insects: from molecular mechanisms to ecology. Trends Plant Sci. 2007;12:564–9 17997347 10.1016/j.tplants.2007.09.004

[7] Pimental D, Goodman RM. Economic impact of insects. In: Goodman RM (ed.), Encyclopedia of Plant and Crop Science. New York, USA: CRC Press, 2004,407–9

[8] Kerchev PI, Fenton B, Foyer CH. et al. Plant responses to insect herbivory: interactions between photosynthesis, reactive oxygen species and hormonal signalling pathways. Plant Cell Environ. 2012;35:441–53 21752032 10.1111/j.1365-3040.2011.02399.x

[9] Bernays EA . Interaction of insects and plants. Sci Prog. 1992;76:247–71

[10] Bronstein JL, Alarcón R, Geber M. The evolution of plant-insect mutualisms. New Phytol. 2006;172:412–28 17083673 10.1111/j.1469-8137.2006.01864.x

[11] Edwards PJ, Wratten SD. Ecology of Insect-Plant Interactions. London, UK: Edward Arnold (Publishers) Ltd.; 1980:

[12] Kessler A, Baldwin IT. Plant responses to insect herbivory: the emerging molecular analysis. Annu Rev Plant Biol. 2002;53:299–328 12221978 10.1146/annurev.arplant.53.100301.135207

[13] Jones JDG, Dangl JL. The plant immune system. Nature. 2006;444:323–9 17108957 10.1038/nature05286

[14] Mithöfer A, Boland W. Recognition of herbivory-associated molecular patterns. Plant Physiol. 2008;146:825–31 18316636 10.1104/pp.107.113118 PMC2259064

[15] Body M, Kaiser W, Dubreuil G. et al. Leaf-miners co-opt microorganisms to enhance their nutritional environment. J Chem Ecol. 2013;39:969–77 23807431 10.1007/s10886-013-0307-y

[16] Giron D, Huguet E, Stone GN. et al. Insect-induced effects on plants and possible effectors used by galling and leaf-mining insects to manipulate their host-plant. J Insect Physiol. 2016;84:70–89 26723843 10.1016/j.jinsphys.2015.12.009

[17] Schultz JC, Edger PP, Body MJA. et al. A galling insect activates plant reproductive programs during gall development. Sci Rep. 2019;9:1833 30755671 10.1038/s41598-018-38475-6 PMC6372598

[18] Gutzwiller F, Dedeine F, Kaiser W. et al. Correlation between the green-island phenotype and Wolbachia infections during the evolutionary diversification of Gracillariidae leaf-mining moths. Ecology. 2015;5:4049–62 10.1002/ece3.1580 PMC4588643 26442762

[19] Lieutier F, Bermudez-Torres K, Cook J. et al. Chapter three – from plant exploitation to mutualism. In: Sauvion N, Thiéry D, Calatayud P-A (eds.), Advances in Botanical Research, Vol. 81. Academic Press, 2004,55–109

[20] Tooker JF, Giron D. The evolution of endophagy in herbivorous insects. Front Plant Sci. 2020;11:581816 33250909 10.3389/fpls.2020.581816 PMC7673406

[21] Shorthouse J, Wool D, Raman A. Gall-inducing insects – nature’s most sophisticated herbivores. Basic Appl Ecol. 2005;6:407–11

[22] Gullan PJ, Miller DR, Cook LG. Gall-inducing scale insects (Hemiptera: Sternorrhyncha: Coccoidea). In: Raman A, Schaefer CW, Withers TW (eds.), Biology, Ecology, and Evolution of Gallinducing Arthropods. Enfield, New Hampshire, USA: Science Publishers, 2004,159–229

[23] Salle JL . Biology of gall inducers and evolution of gall induction in Chalcidoidea (Hymenoptera: Eulophidae, Eurytomidae, Pteromalidae, Tanaostigmatidae, Torymidae). In: Raman A, Schaefer CW, Withers TW (eds.), Biology, Ecology and Evolution of Gall-Inducing Arthropods, Volume 1 and 2. Enfield, New Hampshire, USA: Science Publishers, 2005,507–37

[24] Roskam JC . Evolution of the gall-inducing guild. In: Shorthouse JD, Rohfritsch O (eds.), Biology of Insect-Induced Galls. New York, USA: Oxford University Press, 1992,34–49

[25] Roskam HC . Phylogeny of gall midges (Cecidomyiidae). In: Raman A, Schaefer CW, Withers TW (eds.), Biology, Ecology and Evolution of Gall-Inducing Arthropods, Volume 1 and 2. Enfield, New Hampshire, USA: Science Publishers, 2005,305–19

[26] Stone GN, Schönrogge K. The adaptive significance of insect gall morphology. Trends Ecol Evol. 2003;18:512–22

[27] Raman A, Schafer CW, Withers TM. Galls and gall-inducing arthropods: an overview of their biology, ecology, and evolution. In: Withers TM (ed.), Biology, Ecology, and Evolution of Gall-Inducing Arthropods. Enfield, New Hampshire, USA: Science Publishers, 2005,1–33

[28] Espírito-Santo MM, Wilson Fernandes G. How Many Species of Gall-Inducing Insects Are There on Earth, and Where Are They?. Ann Entomol Soc Am. 2007;100:95–9

[29] Collins JA, Drummond FA. The blueberry gall midge (Diptera: Cecidomyiidae): a recent pest of wild blueberry (Vaccinium angustifolium; Ericales: Ericaceae) and its impact on potential yield. J Econ Entomol. 2019;112:1151–61 30835789 10.1093/jee/toz038

[30] Nacro S, Heinrichs EA, Dakouo D. Estimation of rice yield losses due to the African rice gall midge, Orseolia oryzivora Harris and Gagne. Int J Pest Manag. 1996;42:331–4

[31] Yin L, Burkness EC, Hutchison WD. et al. Effects of foliar Phylloxera (Hemiptera: Phylloxeridae) infestations on wine grape photosynthesis, yield, and fruit quality. J Entomol Sci. 2021;56:504–18

[32] Schmid RB, Knutson A, Giles KL. et al. Hessian fly (Diptera: Cecidomyiidae) biology and management in wheat. J Integr Pest Manag. 2018;9:14

[33] Chen M-S, Liu X, Yang Z. et al. Unusual conservation among genes encoding small secreted salivary gland proteins from a gall midge. BMC Evol Biol. 2010;10:296 20920202 10.1186/1471-2148-10-296 PMC2955719

[34] Hogenhout SA, Bos JIB. Effector proteins that modulate plant–insect interactions. Curr Opin Plant Biol. 2011;14:422–8 21684190 10.1016/j.pbi.2011.05.003

[35] Matsukura K, Matsumura M, Tokuda M. Host manipulation by the orange leafhopper Cicadulina bipunctata: gall induction on distant leaves by dose-dependent stimulation. Naturwissenschaften. 2009;96:1059–66 19513593 10.1007/s00114-009-0566-1

[36] Sopow SL, Shorthouse JD, Strong W. et al. Evidence for long-distance, chemical gall induction by an insect. Ecol Lett. 2003;6:102–5

[37] Body MJA, Zinkgraf MS, Whitham TG. et al. Heritable phytohormone profiles of poplar genotypes vary in resistance to a galling aphid. Mol Plant-Microbe Interact. 2019;32:654–72 30520677 10.1094/MPMI-11-18-0301-R

[38] Cornell HV . The secondary chemistry and complex morphology of galls formed by the Cynipinae (Hymenoptera): why and how? Am Midl Nat. 1983;110:225–34

[39] Shorthouse JD, Rohfritsch O. Biology of Insect-Induced Galls. New York, USA: Oxford University Press; 1992,285

[40] Suzuki H, Yokokura J, Ito T. et al. Biosynthetic pathway of the phytohormone auxin in insects and screening of its inhibitors. Insect Biochem Mol Biol. 2014;53:66–72 25111299 10.1016/j.ibmb.2014.07.008

[41] Tooker JF, Helms AM. Phytohormone dynamics associated with gall insects, and their potential role in the evolution of the gall-inducing habit. J Chem Ecol. 2014;40:742–53 25027764 10.1007/s10886-014-0457-6

[42] Richardson RA, Body M, Warmund MR. et al. Morphometric analysis of young petiole galls on the narrow-leaf cottonwood, Populus angustifolia, by the sugarbeet root aphid, *Pemphigus betae*. Protoplasma. 2017;254:203–16 26739691 10.1007/s00709-015-0937-8 PMC5216080

[43] Mishra P, Saini P, Patni V. Biochemical dynamics during development of insect-induced plant galls: a review. J Plant Dis Prot. 2024;131:1803–18

[44] Isaacs R, Fanning P, Van Timmeren S, Perkins J, Garcia-Salazar C. Biology and Management of Stem Gall Wasp in Highbush Blueberries. 2020. Last accessed August 5th, 2025. Last accessed August 5th, 2025. https://www.canr.msu.edu/blueberries/uploads/files/BlueberryStemGallWasp-WEB-final.pdf.

[45] The Natural History Museum, London . Taxon Record: Hemadas Nubilipennis Ashmead, 1887. Universal Chalcidoidea Database. Accessed August 5, 2025. https://www.nhm.ac.uk/our-science/data/chalcidoids/database/detail.dsml?FamilyCode=PZ&VALAUTHOR=%28Ashmead%29&VALGENUS=Hemadas&HOMCODE=0&VALDATE=1887&VALSPECIES=nubilipennis&ValidAuthBracket=false&&listPageURL=listChalcids%2edsml%3famp%3bFamily%3dPteromalidae%26Species%3dnubilipennis%26amp%3bSuperfamily%3dChalcidoidea%26amp%3bGenus%3dHemadas&tab=distribution.

[46] Blueberry Stem Gall Wasp—Hemadas nubilipennis (Ashmead, 1887). Maryland Biodiverty Project. https://www.marylandbiodiversity.com/view/10395.

[47] Zhang M, Zhou C, Song Z. et al. The first identification of genomic loci in plants associated with resistance to galling insects: a case study in Eucalyptus L’Hér. (Myrtaceae). Sci Rep. 2018;8:2319 29396525 10.1038/s41598-018-20780-9 PMC5797152

[48] Torello Marinoni D, Nishio S, Valentini N. et al. Development of high-density genetic linkage maps and identification of loci for chestnut gall wasp resistance in Castanea spp. Plants. 2020;9:1048 32824716 10.3390/plants9081048 PMC7465717

[49] Moriya S, Iwanami H, Takahashi S. et al. Evaluation and inheritance of crown gall resistance in apple rootstocks. J Japan Soc Hotic Sci. 2008;77:236–41

[50] Data and Insights Center. USHBC. 2020. https://ushbc.blueberry.org/all-resources/data-and-insights-center/ (31 May 2025, date last accessed).

[51] Blueberry Outlook . https://www.reportlinker.com/p05815076/?utm_source=PRN (22 November 2024, date last accessed).

[52] Isaacs R, Edger P, Fanning P. AgBioResearch. AgBioResearch. 2019. https://www.canr.msu.edu/news/combating-the-blueberry-stem-gall-wasp (22 November 2024, date last accessed).

[53] DeVisser AK, Vandervoort C, Isaacs R. et al. Systemic insecticides for control of stem gall wasp in highbush blueberry. J Econ Entomol. 2023;116:1737–49 37598313 10.1093/jee/toad162 PMC10564264

[54] Shorthouse JD, West A, Landry RW. et al. Structural damage by female Hemadas nubilipennis (Hymenoptera: Pteromalidae) as a factor in gall induction on lowbush blueberry. Can Entomol. 1986;118:249–54

[55] West A, Shorthouse JD. Initiation and development of the stem gall induced by Hemadas nubilipennis (Hymenoptera: Pteromalidae) on lowbush blueberry, Vaccinium angustifolium (Ericaceae). Can J Bot. 1989;67:2187–98

[56] De Vos M, Jander G. Choice and no-choice assays for testing the resistance of A. thaliana to chewing insects. J Vis Exp. 2008;15:e683 10.3791/683 PMC2583011 19066591

[57] Giolai M, Paajanen P, Verweij W. et al. Targeted capture and sequencing of gene-sized DNA molecules. Biotechniques. 2016;61:315–22 27938323 10.2144/000114484

[58] Benevenuto J, Ferrão LFV, Amadeu RR. et al. How can a high-quality genome assembly help plant breeders? Gigascience. 2019;8:giz068 31184361 10.1093/gigascience/giz068 PMC6558523

[59] Ferrão LFV, Amadeu RR, Benevenuto J. et al. Genomic selection in an outcrossing autotetraploid fruit crop: lessons from blueberry breeding. Front Plant Sci. 2021;12:676326 34194453 10.3389/fpls.2021.676326 PMC8236943

[60] Martin M . Cutadapt removes adapter sequences from high-throughput sequencing reads. EMBnet.journal. 2011;17:10–2

[61] Li H, Durbin R. Fast and accurate short read alignment with Burrows-Wheeler transform. Bioinformatics. 2009;25:1754–60 19451168 10.1093/bioinformatics/btp324 PMC2705234

[62] Colle M, Leisner CP, Wai CM. et al. Haplotype-phased genome and evolution of phytonutrient pathways of tetraploid blueberry. Gigascience. 2019;8:8 10.1093/gigascience/giz012 PMC6423372 30715294

[63] Danecek P, Bonfield JK, Liddle J. et al. Twelve years of SAMtools and BCFtools. Gigascience. 2021;10:10 10.1093/gigascience/giab008 PMC7931819 33590861

[64] GATK . https://gatk.broadinstitute.org/ (31 May 2025, date last accessed).

[65] Mengist MF, Bostan H, de Paola D. et al. Autopolyploid inheritance and a heterozygous reciprocal translocation shape chromosome genetic behavior in tetraploid blueberry (*Vaccinium corymbosum*). New Phytol. 2023;237:1024–39 35962608 10.1111/nph.18428 PMC10087351

[66] Jacobs M, Thompson S, Platts AE. et al. Uncovering genetic and metabolite markers associated with resistance against anthracnose fruit rot in northern highbush blueberry. Hortic Res. 2023;10:uhad169 38025975 10.1093/hr/uhad169 PMC10660357

[67] Danecek P, Auton A, Abecasis G. et al. The variant call format and VCFtools. Bioinformatics. 2011;27:2156–8 21653522 10.1093/bioinformatics/btr330 PMC3137218

[68] Lipka AE, Tian F, Wang Q. et al. GAPIT: genome association and prediction integrated tool. Bioinformatics. 2012;28:2397–9 22796960 10.1093/bioinformatics/bts444

[69] Ihaka R, Gentleman R. R: a language for data analysis and graphics. J Comput Graph Stat. 1996;5:299–314

[70] Zhang Z, Ersoz E, Lai CQ. et al. Mixed linear model approach adapted for genome-wide association studies. Nat Genet. 2010;42:355–60 20208535 10.1038/ng.546 PMC2931336

[71] Yang S, Zhou X. Accurate and scalable construction of polygenic scores in large biobank data sets. Am J Hum Genet. 2020;106:679–93 32330416 10.1016/j.ajhg.2020.03.013 PMC7212266

[72] Bradski G . The OpenCV Library. Dr. Dobb's J Software Tools. 2000;120:122–5

[73] Dobin A, Davis CA, Schlesinger F. et al. STAR: ultrafast universal RNA-seq aligner. Bioinformatics. 2013;29:15–21 23104886 10.1093/bioinformatics/bts635 PMC3530905

[74] Bolger AM, Lohse M, Usadel B. Trimmomatic: a flexible trimmer for Illumina sequence data. Bioinformatics. 2014;30:2114–20 24695404 10.1093/bioinformatics/btu170 PMC4103590

[75] Li H, Handsaker B, Wysoker A. et al. The sequence alignment/map format and SAMtools. Bioinformatics. 2009;25:2078–9 19505943 10.1093/bioinformatics/btp352 PMC2723002

[76] Anders S, Pyl PT, Huber W. HTSeq—a Python framework to work with high-throughput sequencing data. Bioinformatics. 2014;31:166–9 25260700 10.1093/bioinformatics/btu638 PMC4287950

[77] Robinson MD, McCarthy DJ, Smyth GK. edgeR: a Bioconductor package for differential expression analysis of digital gene expression data. Bioinformatics. 2010;26:139–40 19910308 10.1093/bioinformatics/btp616 PMC2796818

[78] Tang H, Lyons E, Pedersen B. et al. Screening synteny blocks in pairwise genome comparisons through integer programming. BMC Bioinformatics. 2011;12:102 21501495 10.1186/1471-2105-12-102 PMC3088904

[79] Altschul SF, Madden TL, Schäffer AA. et al. Gapped BLAST and PSI-BLAST: a new generation of protein database search programs. Nucleic Acids Res. 1997;25:3389–402 9254694 10.1093/nar/25.17.3389 PMC146917

[80] Langfelder P, Horvath S. WGCNA: an R package for weighted correlation network analysis. BMC Bioinformatics. 2008;9:559 19114008 10.1186/1471-2105-9-559 PMC2631488

[81] Alexa A, Rahnenfuhrer J. Others. topGO: enrichment analysis for gene ontology. *R package version 2.61.1*. 2025. https://bioconductor.org/packages/topGO

[82] Berardini TZ, Mundodi S, Reiser L. et al. Functional annotation of the Arabidopsis genome using controlled vocabularies. Plant Physiol. 2004;135:745–55 15173566 10.1104/pp.104.040071 PMC514112

[83] Wang YE, Kutnetsov L, Partensky A. et al. WebMeV: a cloud platform for analyzing and visualizing cancer genomic data. Cancer Res. 2017;77:e11–4 29092929 10.1158/0008-5472.CAN-17-0802 PMC5679251

[84] Huang M, Liu X, Zhou Y. et al. BLINK: a package for the next level of genome-wide association studies with both individuals and markers in the millions. Gigascience. 2019;8:8 10.1093/gigascience/giy154 PMC6365300 30535326

[85] Clare SJ, Driskill M, Millar TR. et al. Development of a targeted genotyping platform for reproducible results within tetraploid and hexaploid blueberry. Front Hortic. 2024;2:1339310

[86] Colinet D, Cazes D, Belghazi M. et al. Extracellular superoxide dismutase in insects: characterization, function, and interspecific variation in parasitoid wasp venom. J Biol Chem. 2011;286:40110–21 21937434 10.1074/jbc.M111.288845 PMC3220525

[87] Jiang Z, Ge S, Xing L. et al. RLP1.1, a novel wheat receptor-like protein gene, is involved in the defence response against *Puccinia striiformis* f. sp. tritici. J Exp Bot. 2013;64:3735–46 23881396 10.1093/jxb/ert206 PMC3745730

[88] Rao S, Zhou Z, Miao P. et al. Roles of receptor-like cytoplasmic kinase VII members in pattern-triggered immune signaling. Plant Physiol. 2018;177:1679–90 29907700 10.1104/pp.18.00486 PMC6084675

[89] Viswanath KK, Kuo S-Y, Tu C-W. et al. The role of plant transcription factors in the fight against plant viruses. Int J Mol Sci. 2023;24:24 10.3390/ijms24098433 PMC10179606 37176135

[90] Yuan M, Jiang Z, Bi G. et al. Pattern-recognition receptors are required for NLR-mediated plant immunity. Nature. 2021;592:105–9 33692546 10.1038/s41586-021-03316-6 PMC8016741

[91] Couto D, Zipfel C. Regulation of pattern recognition receptor signalling in plants. Nat Rev Immunol. 2016;16:537–52 27477127 10.1038/nri.2016.77

[92] Ngou BPM, Ahn H-K, Ding P. et al. Mutual potentiation of plant immunity by cell-surface and intracellular receptors. Nature. 2021;592:110–5 33692545 10.1038/s41586-021-03315-7

[93] Jung HW, Panigrahi GK, Jung GY. et al. Pathogen-associated molecular pattern-triggered immunity involves proteolytic degradation of core nonsense-mediated mRNA decay factors during the early defense response. Plant Cell. 2020;32:1081–101 32086363 10.1105/tpc.19.00631 PMC7145493

[94] Hoffmann N, Benske A, Betz H. et al. Laccases and peroxidases co-localize in lignified secondary cell walls throughout stem development. Plant Physiol. 2020;184:806–22 32699027 10.1104/pp.20.00473 PMC7536695

[95] Kumar P, Khanal S, da Silva M. et al. Transcriptome analysis of a nematode resistant and susceptible upland cotton line at two critical stages of Meloidogyne incognita infection and development. PLoS One. 2019;14:e0221328 31504059 10.1371/journal.pone.0221328 PMC6736245

[96] Qin S, Fan C, Li X. et al. LACCASE14 is required for the deposition of guaiacyl lignin and affects cell wall digestibility in poplar. Biotechnol Biofuels. 2020;13:197 33292432 10.1186/s13068-020-01843-4 PMC7713150

[97] Ibanez F, Suh JH, Wang Y. et al. Long-term, sustained feeding by Asian citrus psyllid disrupts salicylic acid homeostasis in sweet orange. BMC Plant Biol. 2019;19:493 31718546 10.1186/s12870-019-2114-2 PMC6852996

[98] Mao D, Yu F, Li J. et al. FERONIA receptor kinase interacts with S-adenosylmethionine synthetase and suppresses S-adenosylmethionine production and ethylene biosynthesis in Arabidopsis. Plant Cell Environ. 2015;38:2566–74 25988356 10.1111/pce.12570

[99] Jasrotia S, Jasrotia R. Role of ethylene in combating biotic stress. In: Singh S, Husain T, Singh VP, Tripathi DK, Prasad SM, Dubey NK, eds. Ethylene in Plant Biology. Hoboken: Wiley, 2022,388–97

[100] Kutty NN, Mishra M. Dynamic distress calls: volatile info chemicals induce and regulate defense responses during herbivory. Front Plant Sci. 2023;14:1135000 37416879 10.3389/fpls.2023.1135000 PMC10322200

[101] Mendy B, Wang'ombe MW, Radakovic ZS. et al. Arabidopsis leucine-rich repeat receptor-like kinase NILR1 is required for induction of innate immunity to parasitic nematodes. PLoS Pathog. 2017;13:e1006284 28406987 10.1371/journal.ppat.1006284 PMC5391088

[102] Bassetti N, Caarls L, Bukovinszkine’Kiss G. et al. Genetic analysis reveals three novel QTLs underpinning a butterfly egg-induced hypersensitive response-like cell death in Brassica rapa. BMC Plant Biol. 2022;22:140 35331150 10.1186/s12870-022-03522-y PMC8944062

[103] Little D, Gouhier-Darimont C, Bruessow F. et al. Oviposition by pierid butterflies triggers defense responses in Arabidopsis. Plant Physiol. 2007;143:784–800 17142483 10.1104/pp.106.090837 PMC1803735

