# Supplementary material for: Deciphering the underlying genetics of galling resistance to the blueberry stem gall wasp in northern highbush blueberry
Source: Hortic Res. 2025 Jul 29;12(11):uhaf197. doi: 10.1093/hr/uhaf197 (PMC12552771; doi:10.1093/hr/uhaf197)
Supplement: Web_Material_uhaf197 [file web_material_uhaf197.zip › Supplemental Data S1 - QTL results.pdf]

Supplemental Data S1. QTL results.

1.1 QTL Region 1 (Chromosome 4)

There are thirteen genes within 100 Kb of the most significant SNP on chromosome 4 (Supplemental Data S1). Gene *maker-VaccDscaff4-augustus-gene-361.31* is the closest to the most significant SNP of chromosome 4. Its putative *Arabidopsis* ortholog is AT5G62530 (*ALDEHYDE DEHYDROGENASE 12A1*, *ALDH12A1*). Upregulation of ALDHs leads to the elimination of oxidative stress in plants, as well as proline degradation (Singh et al. 2013). Proline is an amino acid protecting plants from various biotic stresses by scavenging the ROS generated in response to the stress (Hayat et al. 2012). The increase of *ALDH12A1* expression protects the plants from the toxicity generated by ROS or high levels of proline (Hayat et al. 2012). This ortholog of *ALDH12A1* was slightly downregulated on days 1 and 4 in the susceptible genotype (Log2FC = -0.27-0.31). In contrast, this gene was only slightly downregulated on day 1 (Log2FC = -0.26), and upregulated on days 2 and 5-7 (Log2FC = 0.23-0.72) in the resistant genotype.

Another interesting candidate gene within the QTL on chromosome 4 is *maker-VaccDscaff4-augustus-gene-360.31,* a putative ortholog to *Arabidopsis* AT3G47780 (*ATP-BINDING CASSETTE A7*, *ABCA7*)*,* which has a role in cellular phospholipid metabolism (N. Wang et al. 2003). Phospholipid-generated signal transduction is an important component of the plant defense phytohormone, salicylic acid (SA) (Rodas-Junco et al. 2020). The ortholog of *ABCA7* was on average (3 loci) slightly downregulated on day 4 (Log2FC= -0.32) in the susceptible genotype, while in the resistant genotype, its expression was on average slightly upregulated on days 1, 4, and 7 (Log2FC = 0.31-0.50), and downregulated on day 2 (Log2FC = -0.82).

1.2 QTL Region 2 (Chromosome 7)

A total of nine genes were identified within 100 Kb of the most significant SNP on chromosome 7. Eight of these genes were assigned a putative *Arabidopsis* ortholog.

Gene *maker-VaccDscaff7-snap-gene-153.23* is closest to the most significant SNP on chromosome 7. Its putative *Arabidopsis* ortholog is AT4G31120 (*SHK1 BINDING PROTEIN 1*, *SKB1*), which is associated with regulation of transcription — DNA-templated (GO:0006355), and regulation of flower development (GO:0009909). *SKB1* was identified as one of the genes involved in soybean resistance against the common cutworm, *Spodoptera litura*, by altering chromatin processing, DNA replication, transcription, and/or RNA translation (H. Wang et al. 2015). In the susceptible genotype, the ortholog of *SKB1* was downregulated on days 2, 4, and 6 (Log2FC = -0.38-1.03), and slightly upregulated on days 5 and 7 (Log2FC = 0.44-0.49). In the resistant genotype, the ortholog was downregulated on days 1, 3, 5, and 7 (Log2FC = -0.35-0.75), and slightly upregulated on days 4 and 6 (Log2FC = 0.28-0.39).

A noteworthy candidate gene within this region is *maker-VaccDscaff7-augustus-gene-153.15,* which is a putative ortholog of AT4G31160 (*DDB1-CUL4 ASSOCIATED FACTOR 1*, *DCAF1*). *DCAF1* is associated with leaf development (GO:0048366), shoot system development (GO:0048367), phyllome development (GO:0048827), fruit development (GO:0010154), embryo development ending (GO:0009793) and flower development (GO:0009908). It is essential for plant embryogenesis, and potentially involved in multiple developmental pathways (Y. Zhang et al. 2008). In humans, this gene forms a CUL4-DDB1-DCAF1 complex (*CUL4*, *CULLIN 4*; *DDB1*, *DNA DAMAGE-BINDING PROTEIN 1*) that is involved in the regulation of tumor growth (Han et al. 2020). In *A. thaliana* the lack of *DCAF1* eliminates proliferating cells (Han et al. 2020) and leads to embryonic lethality at the late globular stage (Y. Zhang et al.

2008). Mutant plants show defects throughout vegetative and reproductive development with the production of abnormal flowers (Y. Zhang et al. 2008). The blueberry ortholog of *DCAF1* was upregulated on days 1 and 4 (Log2FC = 0.48-0.60) in the susceptible genotype, while in the resistant genotype, it was downregulated on day 5 (Log2FC = -0.56) and slightly upregulated on day 6 (Log2FC = 0.31).

QTL Region 3 (Chromosome 13)

A total of eighteen genes were identified within 100 Kb of the most significant SNP on chromosome 13, with seventeen of these genes assigned to a putative *Arabidopsis* ortholog.

The blueberry gene *snap_masked-VaccDscaff13-processed-gene-97.14* has the *Arabidopsis* ortholog AT1G25450 (*3-KETOACYL-COA SYNTHASE 5*, *KCS5*). *KCS5* is involved in the biosynthesis of very long chain fatty acids (VLCFA; more than 18 carbon atoms), mainly producing C24 to C28 [1]. VLCFA are precursors of abundant lipids involved in different physiological processes in plants, including the production of suberin (C20-C24), storage lipids (C20-C24), phospholipids (C14-C24), sphingolipids (C22-C26), and cuticular waxes (C26-C38) [1–3]. Sphingolipids are involved in *Pieris brassicae* oviposition-induced HR-like cell death in *A. thaliana* and *Brassica nigra* [4]. Oviposition by *P. brassicae* can alter the ratio of the wax components in *A. thaliana* with an increase of tetracosanoic acid and decrease of tetratriacontanoic acid [5]. This change in cuticular wax can inform natural enemies, such as parasitoid wasps, that host-eggs have been laid [5, 6]. *KCS5* was also listed in the top 50 genes differentially expressed in *A. thaliana* between female-associated syncytia (FAS) and male-associated syncytia (MAS) induced by the beet cyst nematode *Heterodera schachtii* [7]. This gene was upregulated (2.5 times) in the FAS, along with genes involved in immune response, nutrient availability, and structural modifications [7]. The ortholog of *KCS5* was strongly

downregulated on days 1-4 and 6-7 in the susceptible genotype (Log2FC = -0.67-1.67. Its expression was downregulated on days 1-2 and 4-6 (Log2FC = -0.28-2.34), and upregulated on days 3 and 7 (Log2FC = 0.42-1.81) in the resistant genotype.

The blueberry gene *snap_masked-VaccDscaff13-processed-gene-97.16* has the *Arabidopsis* ortholog AT5G38450 (*CYTOCHROME P450 FAMILY 735 A1*, *CYP735A1*). It catalyzes the biosynthesis of the cytokinin (CK) *trans*-zeatin (*t*Z) [8]. CKs are important regulators of invertase activity and elicitors of sink strength. This gene was reported as strongly upregulated during the development of phylloxera galls on grapevine leaves [9]. Additionally, poplar trees susceptible to the gall-inducing aphid, *Pemphigus betae*, contained higher constitutive CKs in leaves, and had a greater ability of aphids to elicit CK accumulation, compared to resistant poplar trees [10]. Increased CK concentrations may facilitate forming strong photosynthate sinks in the galls, a requirement for the success of gall-inducing insects [10]. Therefore, CKs may be involved in the underlying mechanism of gall failure and tree resistance. In the susceptible genotype, the ortholog of *CYP735A1* was upregulated on days 3-4 and 7 (Log2FC = 0.44-0.56), and downregulated on days 5 (Log2FC = -0.46). In the resistant genotype it was downregulated on days 1 and 5 (Log2FC = -0.50-1.22), and upregulated on day 6 (Log2FC = 0.62).

Gene *maker-VaccDscaff13-augustus-gene-97.23* has the *Arabidopsis* ortholog AT5G38460, which is an uncharacterized gene that encodes a protein that is part of the ALG8 glycosyltransferase family. This gene was identified as a candidate for disease resistance against the bacteria *Pseudomonas syringae* [11]. The blueberry gene corresponding to this ortholog was strongly downregulated on day 1 (Log2FC = -1.21), and strongly upregulated on days 5-6 (Log2FC = 0.81-1.79), in the susceptible genotype. In contrast, its expression was slightly

downregulated on days 1-3 (Log2FC = -0.38-0.45), and upregulated on day 6 (Log2FC = 0.75), in the resistant genotype.

The expression pattern of the uncharacterized gene AT5G15270 significantly altered potato blight pathogen *Phytophthora infestans* infestation [12]. The overexpression of this gene led to an enhanced pathogen colonization of the host plant [12]. In the susceptible genotype, the ortholog of AT5G15270 was downregulated on days 1-2 (Log2FC = -0.42-1.26), and upregulated on days 4 and 6-7 (Log2FC = 0.66-0.88). In the resistant genotype, its expression was upregulated on days 1-3 and 7 (Log2FC = 0.29-1.21), and downregulated on days 4-6 (Log2FC = -0.28-1.04).

Gene *maker-VaccDscaff13-augustus-gene-97.26* has the *Arabidopsis* ortholog AT5G16050 (*GENERAL REGULATORY FACTOR 5*, *GRF5*). *GRF5* is a growth regulating factor encoding a transcription activator, and is involved in the regulation of cell proliferation (Horiguchi, Kim, and Tsukaya 2005). *GRF5* was upregulated in response to *Brevicoryne brassicae* aphid feeding in *A. thaliana*, as part of the biotic stress signaling process (Barah 2013). Another study revealed a role for microRNAs (miRNAs), such as Gma-miR396e which targets *GRF3* and *GRF5*, in *Glycine max* soybean resistance to bean pyralid larvae (*Lamprosema indicata*) (Zeng et al. 2019). Gma-miR396e could therefore increase the resistance levels of the highly-resistant material against *L. indicata* larvae infestation through the negative target regulation of the GRF genes (Zeng et al. 2019). Additionally, in *A. thaliana*, miRNAs have a role in host-plant gene silencing mechanisms during *Heterodera schachtii* beet cyst nematode parasitism (Hewezi et al. 2008). *GRFs* are also induced during syncytium formation by the soybean cyst nematode *Heterodera glycines* in soybean *G. max* ("On the Molecular Biology and Evolution of Plant Parasitism by Nematodes" 2016). In the susceptible genotype, the ortholog of *GRF5* was downregulated on days 1, 3, and 6-7 (Log2FC = -0.28-1.69), and upregulated on days

2 and 5 (Log2FC = 0.73-0.84). In contrast, its expression was upregulated on days 1, 4, and 6-7 (Log2FC = 0.35-1.64), and downregulated on days 2-3 and 5 (Log2FC = -0.35-1.43).

Gene *maker-VaccDscaff13-augustus-gene-98.27* has the *Arabidopsis* ortholog of AT3G02580 (*STEROL 1*, *STE1*). It is a sterol biosynthetic enzyme that catalyzes the Δ^7 sterol C-5 desaturation step [13]. Sterols are precursors of the plant hormone brassinosteroids (BRs) [14]. Sterols are also a required precursor of insects' steroid hormones involved in the regulation of molting and developmental processes [15]. However, insects are sterol auxotrophs and thus, rely solely on dietary sterol sources. Altering the expression of *STE1* leads to the production of unsuitable sterols [15]. In a recent study, the alteration of sterol concentration and/or suitability was suggested as a novel strategy to control herbivorous insect pests [16]. In the susceptible genotype, the ortholog of *STE1* was strongly downregulated on days 3 and 6 (Log2FC = -0.59-1.27), and slightly upregulated on day 5 (Log2FC = 0.25), while in the resistant genotype, its expression was upregulated on days 1 and 5 (Log2FC = 0.59-0.70), and downregulated on days 2 and 7 (Log2FC = -0.73-0.86).

Gene *maker-VaccDscaff13-augustus-gene-98.24* is an ortholog of the uncharacterized gene AT1G67050, which is a membrane-associated kinase regulator. This gene was downregulated in *A. thaliana* after recognition of Turnip Crinkle Virus (TCV), leading to HRT-mediated resistance (HRT is a member of the class of resistance (R) genes) with HR [17]. In our system, its ortholog was upregulated on days 1 and 4-6 (Log2FC = 0.36-1.85), and downregulated on days 2-3 and 7 (Log2FC = -0.47-1.11). In contrast, its expression was downregulated on days 2 and 4 (Log2FC = -0.51-0.75), and strongly upregulated on day 3 (Log2FC = 1.23).

maker-VaccDscaff13-snap-gene-98.35 has the *Arabidopsis* ortholog of AT5G16150 (*PLASTIDIC GLUCOSE TRANSLOCATOR*, *PGLCT*). It is involved in carbohydrate

transport[18]. In French Marigold, the alteration of sugar metabolism, including *PGLCT* gene expression, enhanced the feeding behavior of Colorado potato beetle larvae[18]. In our system, the ortholog of *PGLCT* was upregulated on days 1-2 and 5 (Log2FC = 0.51-0.62), and downregulated on day 4 (Log2FC = -0.55), in the susceptible genotype. Its expression was upregulated on day 1 (Log2FC = 0.50), and downregulated on day 3 (Log2FC = -0.52), in the resistant genotype.

maker-VaccDscaff13-augustus-gene-98.25 has the *Arabidopsis* ortholog AT5G22460, which is an uncharacterized gene in the alpha/beta-hydrolases superfamily protein, involved in cell wall modification [19]. The loss-of-function of *WRKY19*, a regulator of basal levels of immunity, led to a decrease in AT5G22460 expression during the root-knot nematode *Meloidogyne incognita* infection in *A. thaliana* [20]. The ortholog of AT5G22460 was upregulated on days 3 and 6 (Log2FC = 0.53), and downregulated on day 5 (Log2FC = -0.54) in the susceptible genotype. In contrast, its expression was upregulated on days 1 and 3-6 (Log2FC = 0.33-0.55), and strongly downregulated on day 2 (Log2FC = -0.95) in the resistant genotype.

augustus_masked-VaccDscaff13-processed-gene-98.8 has the *Arabidopsis* ortholog AT5G16200, which is an uncharacterized gene encoding for a 50S ribosomal protein-like protein. This gene is thought to be involved in the mitochondrial stress response in *A. thaliana* [21]. Mitochondria are targets of ROS, but also major sources of cellular ROS, which can lead to cell death [21]. A recent study identified mitochondria as a signal hub leading to induced plant resistance by epigenetic-lasting changes, with epigenetic mechanisms being mainly related to the control of chromatin structure [22]. In the susceptible genotype, the ortholog of AT5G16200 was strongly downregulated on days 4-5 (Log2FC = -1.54-2.77), while in the resistant genotype, its expression was strongly upregulated on day 1 (Log2FC = 3.11), and strongly downregulated on days 3 and 5 (Log2FC = -1.38-1.51).

maker-VaccDscaff13-augustus-gene-98.30 has the *Arabidopsis* ortholog of AT3G02570 (*MATERNAL EFFECT EMBRYO ARREST 31* also known as *PHOSPHOMANNOSE ISOMERASE 1*, *MEE31* / *PMI1*). It is associated with embryo development ending in seed dormancy (GO0009793). This gene is also an ascorbate (vitamin C) biosynthetic gene. Ascorbate, the most abundant water-soluble antioxidant in plants, provides the first line of defense against damaging ROS, and helps protect plant cells from many factors that induce oxidative stress, including wounding and pathogen attack [23]. In our system, the ortholog of *MEE31* was upregulated on days 1 and 5 (Log2FC = 0.63-1.05), and downregulated on days 4 and 7 (Log2FC = -0.53-1.30), in the susceptible genotype. In contrast, its expression was downregulated on days 2 and 4 (Log2FC = -0.76-0.82), and upregulated on day 6 (Log2FC = 0.51), in the resistant genotype.

The gene *maker-VaccDscaff13-augustus-gene-98.29* is closest to the most significant SNP on Chromosome 13, and a putative ortholog to AT1G67100 (*LOB DOMAIN-CONTAINING PROTEIN 40*, *LBD40*). *LBD40* is a gibberellin- (GA) and DELLA-responsive gene that is predicted to encode transcriptional regulators, and has a role in plant defense against pathogens (Zentella et al. 2007). *LBD40* expression profiles were previously shown to be altered by pathogens, decreased expression following treatment with the leaf-infecting necrotrophic fungal pathogen *Alternaria brassicicola*, and increased following treatment with the leaf-infecting necrotrophic fungal pathogen *Botrytis cinerea*, root pathogen *Phytophthora parasitica* (oomycete), and root-knot nematode *Meloidogyne incognita* (Thatcher, Kazan, and Manners 2012). Additionally, *LBD40* was shown to be upregulated in Scots pine (*Pinus sylvestris*) upon feeding by the large pine weevil (*Hylobius abietis*) (Kovalchuk et al. 2015). In our system, in the susceptible genotype, the ortholog of *LBD40* was downregulated on days 1-2 (Log2FC = -0.30-0.51), and upregulated on days 3-4 and 6-7 (Log2FC = 0.55-0.79). In the resistant genotype, we

observed the opposite pattern with gene expression being upregulated on days 2-3 and 5-6 (Log2FC = 0.60-1.02), and downregulated on days 4 and 7 (Log2FC = -0.83-1.02).

Another interesting candidate gene is *maker-VaccDscaff13-snap-gene-98.37*, which is a putative ortholog of AT5G23310 (*FE SUPEROXIDE DISMUTASE 3*, *FSD3*, *SOD3*). *SOD3* is an antioxidant gene protecting against ROS by detoxifying superoxide. It is associated with removal of superoxide radicals (GO:0019430) and superoxide dismutase activity (GO:0004784) (Sytykiewicz 2014). Interestingly, the transcripts of this gene were previously found in the venom apparatus of two *Leptopilina sp.* endoparasitoid wasps of *Drosophila* (Colinet et al. 2011). These wasp's venom proteins are secreted and injected with their eggs to protect them from the host immune response in order to ensure successful parasitism, suggesting that insect extracellular SODs can be used as virulence factors to counteract the host's immune response (Colinet et al. 2011). Additionally, a recent study identified *SOD3* in Steinernema and Strongyloididae nematode larvae (L. Xu et al. 2020). They reported a divergence between parasitic nematodes and their closest free-living nematode, the specifically high expression of *SOD3* in the female parasitic stage, and presence in excretory-secretory proteome of Strongyloides suggest that *SOD3* may play a role in the parasitism process (L. Xu et al. 2020). In the susceptible parent, the expression of *SOD3*'s ortholog was upregulated on days 4-5 (Log2FC = 0.58-0.77), and downregulated on days 6-7 (Log2FC = -0.61-1.21). In the resistant genotype, its expression remained unchanged when compared with controls during the seven days of our study.

maker-VaccDscaff13-augustus-gene-98.31 has the *Arabidopsis* ortholog AT5G16160, which is an uncharacterized gene that was listed in the 50 most downregulated genes after induction of the *Agrobacterium tumefaciens* virulence protein *VirE3* on *A. thaliana* [24]. In the susceptible genotype, the ortholog of AT5G16160 was strongly upregulated on days 2-5

(Log2FC = 0.84-1.77), and downregulated on day 7 (Log2FC = -0.88). In contrast, in the resistant genotype, its expression was strongly downregulated on days 2 and 7 (Log2FC = -1.93-2.04), and upregulated on day 6 (Log2FC = 0.84).

maker-VaccDscaff13-snap-gene-99.37 has the *Arabidopsis* ortholog AT1G66880. The uncharacterized gene AT1G66880 encodes a protein kinase superfamily protein, belonging to the family LRKL and subfamily LRK10L-1. In *A. thaliana*, this gene was upregulated in response to Turnip mosaic virus [25], and green peach aphid *Myzus persicae* [26]. In a study on the induction of innate immunity to parasitic nematodes, AT1G66880 was induced in response to HsNemaWater treatment (a cocktail of nematode elicitors) in *A. thaliana* as well [27]. This gene was also upregulated in response to oviposition by *P. brassicae* in the wild black mustard *Brassica rapa* [28] and in *A. thaliana* [29], as well as upon treatment with the bacterial elicitor flg22 in *A. thaliana* [29]. In our system, the ortholog of AT1G66880 was upregulated on days 1 and 5 (Log2FC = 0.62-0.92), and downregulated on days 2-4 and 6-7 (Log2FC = -0.43-1.02), in the susceptible genotype. In contrast, its expression was downregulated on days 1 and 4 (Log2FC = -0.42-0.48), and upregulated on days 2 and 6 (Log2FC = 0.55-1.08), in the resistant genotype.

augustus_masked-VaccDscaff13-processed-gene-97.1 has the *Arabidopsis* ortholog AT5G16080 (*CARBOXYESTERASE 17*, *CXE17*, AT5G16080). In our system, the ortholog of AT5G16080 was downregulated on days 1 and 7 (Log2FC = -0.83, -0.98) in the susceptible genotype and downregulated on day 7 (Log2FC = -0.90) in the resistant genotype.

QTL Region 4 (Chromosome 39)

The nearest nearest gene is *augustus_masked-VaccDscaff39-processed-gene-51.0*, a putative ortholog of AT1G47890 (*RECEPTOR-LIKE PROTEIN 7*, *RLP7*), which belongs to a *Verticillium* wilt resistance Ve-like family of genes, and is involved in the resistance against the

highly aggressive fungus *Verticillium dahlia* (Ojeda-Martinez, Diaz, and Santamaria 2021). Receptor-like proteins (RLPs) have been found to play a role in disease resistance (G. Wang et al. 2008). Additionally, the expression of this gene was altered in response to the lepidopteran *Pieris brassicae* oviposition on *A. thaliana* (Ojeda-Martinez, Diaz, and Santamaria 2021). In our system, the ortholog of *RLP7* was only upregulated on day 1 (Log2FC = 0.48) in the susceptible genotype, while its expression was upregulated on days 1-2, 4, and 7 (Log2FC = 0.33-1.31) and downregulated on day 6 (Log2FC = -0.30) in the resistant genotype.

An additional candidate gene within this region is *maker-VaccDscaff39-augustus-gene-52.32*, a putative ortholog to AT1G07530 (*SCARECROW-LIKE 14*, *SCL14*). *SCL14* is a GRAS regulatory protein, and is critical for the production of *cis*-jasmone-induced (*c*J) indirect defenses, and the regulation of *c*J-induced gene expression. The volatile organic compound (VOC) *c*J is produced by the metabolism of jasmonic acid (JA) *via* a decarboxylation, and enhances direct and indirect plant defense against herbivorous insects (Matthes et al. 2010). The emission of *c*J by infested plants is a repellent for damson-hop aphids (*Phorodon humuli*) and grain aphids (*Sitobion avenae*), while it is an attractant for insects such as seven-spot ladybug (*Coccinella septempunctata)* and aphid parasitoid (*Aphidius ervi*) which prey upon the aphids (Birkett et al. 2000). The expression of the *SCL14* ortholog in samples subjected to oviposition remained similar to controls on days 1-7 in the susceptible genotype, while its expression was upregulated on days 1 and 4 (Log2FC = 0.59-1.10), and downregulated on day 2 (Log2FC = -0.31), in the resistant genotype.

maker-VaccDscaff39-augustus-gene-52.33 has the *Arabidopsis* ortholog AT5G37600 (*GLUTAMINE SYNTHASE CLONE R1* or *GLUTAMINE SYNTHETASE CYTOSOLIC ISOZYME 1-1*, *GSR1* / *GLN1-1*). *GSR1* is a co-repressor with *AUXIN RESPONSE 16* (*ARF16*), regulating auxin-induced seed germination [30], and plays a key role during ABA-mediated brassinosteroid

(BR) biosynthesis [31]. This gene was also upregulated in response to the phloem-feeding silverleaf whitefly nymphs, *Bemisia tabaci* [32]. In the susceptible genotype, the expression of *GSR1* ortholog was upregulated on days 1 and 3-4 (Log2FC = 0.50-0.99), and downregulated on day 2 (Log2FC = -0.55). In the resistant genotype, its expression was upregulated on days 1-3 and 7 (Log2FC = 0.27-0.84), and downregulated on day 4 (Log2FC = -0.72).

Gene *augustus_masked-VaccDscaff39-processed-gene-52.7* has the uncharacterized *Arabidopsis* ortholog AT5G37570, encoding a pentatricopeptide repeat (PPR-like) superfamily protein. It was shown to be differentially expressed during induced resistance against pathogens [33]. In the susceptible genotype, the ortholog of AT5G37570 was downregulated on day 7 (Log2FC = -0.60), while its expression remained similar to controls on all seven days in the resistant genotype.

snap_masked-VaccDscaff39-processed-gene-51.10 has the uncharacterized *Arabidopsis* ortholog of AT5G37540, which is part of the eukaryotic aspartyl protease family protein [34]. Aspartyl protease is a protease inhibitor, a component of plant defense proteins that inhibits the protease activity responsible for protein digestion of the attacking herbivorous insect [35]. Some aspartyl proteases, such as phytepsin and nucellin in barley, are also involved in plant programmed cell death [36]. The expression of AT5G37540 was differentially regulated by Pierid butterfly oviposition on *A. thaliana* [29]. The ortholog of this gene was upregulated on day 1 (Log2FC = 0.87), and downregulated on days 5-6 (Log2FC = -0.70-1.17), in the susceptible genotype. In contrast, its expression was upregulated on days 1-3 and 5 (Log2FC = 0.31-1.30), and downregulated on day 4 (Log2C = -1.12), in the resistant genotype.

Bibliography

1. Batsale M, Bahammou D, Fouillen L, Mongrand S, Joubès J, Domergue F (2021) Biosynthesis and Functions of Very-Long-Chain Fatty Acids in the Responses of Plants to Abiotic and Biotic Stresses. *Cells* , 10(6)https://doi.org/10.3390/cells10061284

2. Bach L, Faure J-D (2010) Role of very-long-chain fatty acids in plant development, when chain length does matter. *Comptes rendus biologies*, 333(4):361–370. https://doi.org/10.1016/j.crvi.2010.01.014

3. Alberts B, Johnson A, Lewis J, Raff M, Roberts K, Walter P (2002) The Lipid Bilayer. https://www.ncbi.nlm.nih.gov/books/NBK26871/

4. Groux R, Fouillen L, Mongrand S, Reymond P (2021) Sphingolipids are involved in Pieris brassicae egg-induced cell death in Arabidopsis thaliana. *bioRxiv*, :2021.07.09.451813. https://doi.org/10.1101/2021.07.09.451813

5. Blenn B, Bandoly M, Küffner A, Otte T, Geiselhardt S, Fatouros NE, Hilker M (2012) Insect egg deposition induces indirect defense and epicuticular wax changes in Arabidopsis thaliana. *Journal of chemical ecology*, 38(7):882–892. https://doi.org/10.1007/s10886-012-0132-8

6. Hilker M, Fatouros NE (2015) Plant responses to insect egg deposition. *Annual review of entomology*, 60:493–515. https://doi.org/10.1146/annurev-ento-010814-020620

7. Anjam MS (2017) Identification of host genes with a role in sex determination of the plant parasitic cyst nematode, Heterodera schachtii. https://bonndoc.ulb.uni-bonn.de/xmlui/handle/20.500.11811/7027

8. Takei K, Yamaya T, Sakakibara H (2004) Arabidopsis CYP735A1 and CYP735A2 encode cytokinin hydroxylases that catalyze the biosynthesis of trans-Zeatin. *The Journal of biological chemistry*, 279(40):41866–41872. https://doi.org/10.1074/jbc.M406337200

9. Body MJA, Appel HM, Edger PP, Schultz JC (2019) A gall-forming insect manipulates hostplant phytohormone synthesis, concentrations, and signaling. *bioRxiv*, :658823. https://doi.org/10.1101/658823

10. Body MJA, Zinkgraf MS, Whitham TG, Lin C-H, Richardson RA, Appel HM, Schultz JC (2019) Heritable Phytohormone Profiles of Poplar Genotypes Vary in Resistance to a Galling Aphid. *Molecular plant-microbe interactions: MPMI*, 32(6):654–672. https://doi.org/10.1094/MPMI-11-18-0301-R

11. Ravichandran S (2014) An Arabidopsis Purple Acid Phosphatase5 (pap5) is Essential for Maintaining Basal Resistance Against Pseudomonas Syringae. https://dalspace.library.dal.ca/handle/10222/55961

12. McLellan H, Harvey SE, Steinbrenner J, Armstrong MR, He Q, Clewes R, Pritchard L, Wang W, Wang S, Nussbaumer T, Dohai B, Luo Q, Kumari P, Duan H, Roberts A, Boevink PC, Neumann C, Champouret N, Hein I, Falter-Braun P, Beynon J, Denby K, Birch PRJ (2022) Exploiting breakdown in nonhost effector–target interactions to boost host disease resistance. *Proceedings of the National Academy of Sciences*, 119(35):e2114064119. https://doi.org/10.1073/pnas.2114064119

13. Silvestro D, Andersen TG, Schaller H, Jensen PE (2013) Plant sterol metabolism. Δ(7)-Sterol-C5-desaturase (STE1/DWARF7), Δ(5,7)-sterol-Δ(7)-reductase (DWARF5) and Δ(24)-sterol-Δ(24)-reductase (DIMINUTO/DWARF1) show multiple subcellular localizations in Arabidopsis thaliana (Heynh) L. *PloS one*, 8(2):e56429. https://doi.org/10.1371/journal.pone.0056429

14. Bajguz A, Chmur M, Gruszka D (2020) Comprehensive Overview of the Brassinosteroid Biosynthesis Pathways: Substrates, Products, Inhibitors, and Connections. *Frontiers in plant science*, 11:1034. https://doi.org/10.3389/fpls.2020.01034

15. Jing X, Behmer ST (2020) Insect Sterol Nutrition: Physiological Mechanisms, Ecology, and Applications. *Annual review of entomology*, 65:251–271. https://doi.org/10.1146/annurev-ento-011019-025017

16. Chen W (2019) Manipulation of plant sterol profile and its effect on insects. https://oaktrust.library.tamu.edu/handle/1969.1/189002

17. (2016) Role of Plasmodesmata Localizing-and Double-Stranded RNA Binding-Proteins in Systemic Immunity and Plant Defense. https://search.proquest.com/openview/eabf1107ef53a093790fb885a628c756/1?pq-origsite=gscholar&cbl=18750&diss=y

18. Stupar S, Dragićević M, Tešević V, Stanković-Jeremić J, Maksimović V, Ćosić T, Devrnja N, Tubić L, Cingel A, Vinterhalter B, Ninković S, Savić J (2021) Transcriptome Profiling of the Potato Exposed to French Marigold Essential Oil with a Special Emphasis on Leaf Starch Metabolism and Defense against Colorado Potato Beetle. *Plants*, 10(1)https://doi.org/10.3390/plants10010172

19. Zhou X-Y, Song L, Xue H-W (2013) Brassinosteroids regulate the differential growth of Arabidopsis hypocotyls through auxin signaling components IAA19 and ARF7. *Molecular plant*, 6(3):887–904. https://doi.org/10.1093/mp/sss123

20. (2019) Susceptibility Genes, a Novel Strategy to Improve Resistance against the Root-Knot Nematode Meloidogyne incognita. https://search.proquest.com/openview/976d69a226c6c8165805a85dc144a769/1?pq-origsite=gscholar&cbl=2026366&diss=y

21. Van Aken O, Zhang B, Carrie C, Uggalla V, Paynter E, Giraud E, Whelan J (2009) Defining the mitochondrial stress response in Arabidopsis thaliana. *Molecular plant*, 2(6):1310–1324. https://doi.org/10.1093/mp/ssp053

22. López Sánchez A, Hernández Luelmo S, Izquierdo Y, López B, Cascón T, Castresana C (2021) Mitochondrial Stress Induces Plant Resistance Through Chromatin Changes. *Frontiers in plant science*, 12:704964. https://doi.org/10.3389/fpls.2021.704964

23. Suza WP, Avila CA, Carruthers K, Kulkarni S, Goggin FL, Lorence A (2010) Exploring the impact of wounding and jasmonates on ascorbate metabolism. *Plant physiology and biochemistry: PPB / Societe francaise de physiologie vegetale*, 48(5):337–350. https://doi.org/10.1016/j.plaphy.2010.02.004

24. Niu X, Henkel C, Heusden GPH van, Hooykaas PJJ The influence of the Agrobacterium tumefaciens virulence proteins VirE3 and VirF on the Arabidopsis thaliana transcriptome. https://scholarlypublications.universiteitleiden.nl/access/item%3A2922258/download

25. (2010) The regulation of host translation initiation in plant-pathogen interactions. https://search.proquest.com/openview/201b2d076e540c2b57f2df69817d2527/1?pq-origsite=gscholar&cbl=18750

26. (2005) Molecular insights into Arabidopsis response to Myzus persicae Sulzer (green peach aphid). https://search.proquest.com/openview/1ac9497fd676562bade6d669ea5675fc/1?pq-origsite=gscholar&cbl=18750&diss=y

27. Mendy B, Wang'ombe MW, Radakovic ZS, Holbein J, Ilyas M, Chopra D, Holton N, Zipfel C, Grundler FMW, Siddique S (2017) Arabidopsis leucine-rich repeat receptor-like kinase NILR1 is required for induction of innate immunity to parasitic nematodes. *PLoS pathogens*, 13(4):e1006284. https://doi.org/10.1371/journal.ppat.1006284

28. Bassetti N, Caarls L, Bukovinszkine'Kiss G, El-Soda M, Veen J van, Bouwmeester K, Zwaan BJ, Schranz ME, Bonnema G, Fatouros NE (2022) Genetic analysis reveals three novel QTLs underpinning a butterfly egg-induced hypersensitive response-like cell death in Brassica rapa. *BMC plant biology*, 22(1):140. https://doi.org/10.1186/s12870-022-03522-y

29. Little D, Gouhier-Darimont C, Bruessow F, Reymond P (2007) Oviposition by pierid butterflies triggers defense responses in Arabidopsis. *Plant physiology*, 143(2):784–800. https://doi.org/10.1104/pp.106.090837

30. Ye Y, Gong Z, Lu X, Miao D, Shi J, Lu J, Zhao Y (2016) Germostatin resistance locus 1 encodes a PHD finger protein involved in auxin-mediated seed dormancy and germination. *The Plant journal: for cell and molecular biology*, 85(1):3–15. https://doi.org/10.1111/tpj.13086

31. Li Q, Xu F, Chen Z, Teng Z, Sun K, Li X, Yu J, Zhang G, Liang Y, Huang X, Du L, Qian Y, Wang Y, Chu C, Tang J (2021) Synergistic interplay of ABA and BR signal in regulating plant growth and adaptation. *Nature plants*, 7(8):1108–1118. https://doi.org/10.1038/s41477-021-00959-1

32. Kempema LA, Cui X, Holzer FM, Walling LL (2007) Arabidopsis transcriptome changes in response to phloem-feeding silverleaf whitefly nymphs. Similarities and distinctions in responses to aphids. *Plant physiology*, 143(2):849–865. https://doi.org/10.1104/pp.106.090662

33. Sundar AR, Selvaraj N, Muthiah M, Ramadass A, Malathi P, Viswanathan R (2012) Induced resistance-a potential supplementary strategy for the management of red rot in sugarcane. *Functional plant science & biotechnology*, 6:63–72. https://www.researchgate.net/profile/N-M-R-Ashwin/publication/278713077_Induced_Resistance_-_A_Potential_Supplementary_Strategy_for_the_Management_of_Red_Rot_in_Sugarcane/links/57e50f9608ae7c90cefc25bd/Induced-Resistance-A-Potential-Supplementary-Strategy-for-the-Management-of-Red-Rot-in-Sugarcane.pdf

34. Berardini TZ, Reiser L, Li D, Mezheritsky Y, Muller R, Strait E, Huala E (2015) The Arabidopsis information resource: Making and mining the "gold standard" annotated reference

plant genome. *Genesis* , 53(8):474–485. https://doi.org/10.1002/dvg.22877

35. Zhu-Salzman K, Zeng R (2015) Insect response to plant defensive protease inhibitors. *Annual review of entomology*, 60:233–252. https://doi.org/10.1146/annurev-ento-010814-020816

36. Simões I, Faro C (2004) Structure and function of plant aspartic proteinases. *European journal of biochemistry / FEBS*, 271(11):2067–2075. https://doi.org/10.1111/j.1432-1033.2004.04136.x
