# Supplementary material for: Deciphering the underlying genetics of galling resistance to the blueberry stem gall wasp in northern highbush blueberry
Source: Hortic Res. 2025 Jul 29;12(11):uhaf197. doi: 10.1093/hr/uhaf197 (PMC12552771; doi:10.1093/hr/uhaf197)
Supplement: Web_Material_uhaf197 [file web_material_uhaf197.zip › Supplemental Data S2 - DEG results.docx]

**Supplemental Data S2.** Significantly differentially expressed genes (DEGs).

***1. DEGs unique to the susceptible genotype***

***1.1 Day 1***

On day 1, we found 15 differentially regulated *Arabidopsis* orthologs, 6 genes (6 loci) were upregulated and 9 genes (10 loci) were downregulated.

***Upregulated DEGs*** *—* Among the upregulated genes, we identified orthologs of *ENHANCED DISEASE SUSCEPTIBILITY 1* (*EDS1*, Log2FC = 1.74), and *CELL DIVISION CYCLE PROTEIN 26* (*CDC26*, Log2FC = 3.32).

*EDS1* is indispensable for the function of plant disease resistance (R) genes [[1]](https://paperpile.com/c/slGjxJ/RCf4v).

*CDC26* controls cell division, growth and embryo development [[2]](https://paperpile.com/c/slGjxJ/XrjRF).

***Downregulated DEGs*** *—* Among the downregulated genes, we identified *PEROXIDASE 42* (*PER42*/*PRXR1*, Log2FC = -1.59) and *EXTENSIN 21* (*EXT21*, Log2FC = -1.14-1.20, 2 loci).

*PRXR1* is a member of the peroxidase superfamily protein involved in the removal of H_2_O_2_ [[3]](https://paperpile.com/c/slGjxJ/nluvH). Additionally, *PRXR1* has an isochorismate pyruvate lyase activity, and is involved in the conversion of isochorismate to salicylic acid (SA) [[3]](https://paperpile.com/c/slGjxJ/nluvH).

*EXT21* is involved in plant cell wall organization [[4]](https://paperpile.com/c/slGjxJ/VgBMb).

***1.2 Day 2***

On day 2, we did not find any DEGs. We found two blueberry genes as DEGs unique to the susceptible genotype on day 2; however, we were unable to recover an *Arabidopsis* ortholog for both.

***1.3 Day 3***

On day 3, we identified 5 differentially regulated *Arabidopsis* orthologs, 1 gene (1 locus) was upregulated and 4 genes (4 loci) were downregulated.

***Upregulated DEGs*** *—* Among the upregulated genes, we identified *RECEPTOR LIKE PROTEIN 1* (*RLP1*, Log2FC = 4.32).

*RLP1* is a leucine-rich repeat receptor protein (LRR-RP)-type pattern recognition receptor (PRRs), and acts as a receptor against some pathogens [[5, 6]](https://paperpile.com/c/slGjxJ/WKgGb+9nuk4).

***1.4 Day 4***

On day 4, we found 11 differentially regulated *Arabidopsis* orthologs, 5 genes (5 loci) were upregulated and 6 genes (6 loci) were downregulated.

***Downregulated DEGs*** *—* Among the downregulated genes, we identified *GLUCURONOXYLAN METHYLTRANSFERASE 1* (*GXM1*, Log2FC = -3.04).

*GXM1* plays a role in xylan biosynthesis and plant secondary cell wall biogenesis [[7]](https://paperpile.com/c/slGjxJ/ABjSu). More specifically, *GXM1* is involved in 4-*O*-methylation of glucuronic acid side chains [[8]](https://paperpile.com/c/slGjxJ/odSBS).

***1.5 Day 5***

On day 5, we found 5 differentially regulated *Arabidopsis* orthologs, all 5 genes (5 loci) were downregulated.

***Downregulated DEGs*** *—* Among the downregulated genes, we identified *IAA-LEUCINE RESISTANT 3* (*ILR3*, Log2FC = -2.56), and *SUBTILISIN-LIKE SERINE PROTEASE 1.7* (*ARA12* or *SBT1.7*, Log2FC = -1.12), as well as a GDSL-motif esterase / acyltransferase / lipase (AT5G22810, Log2FC = -3.01).

*ILR3* is a bHLH transcription factor that modulates multiple stress responses in *A. thaliana*. *ILR3* functions in a regulatory network that regulates aliphatic glucosinolate (GSL) biosynthesis and pathogen response [[9]](https://paperpile.com/c/slGjxJ/kMYZN). GSL are produced as part of plant defenses against some pathogens, such as the sugarbeet root cyst nematode (*Heterodera schachtii*) in *A. thaliana* [[9]](https://paperpile.com/c/slGjxJ/kMYZN). In mutants, long-chain GSL concentrations were correlated with elevated nematode resistance [[9]](https://paperpile.com/c/slGjxJ/kMYZN).

*ARA12* (or *SBT1.7*) is a subtilisin-like protease that negatively modifies/regulates factors that contribute to cell wall weakening, such as pectin methylesterase (PME) activity during the later stages of seed development [[10]](https://paperpile.com/c/slGjxJ/B2eNx). *ARA12* triggers the accumulation and/or activation of enzymes involved in cell wall modification leading to the outer primary cell wall loosening [[11]](https://paperpile.com/c/slGjxJ/9F6zv). The protease was found predominantly in the silique and stem of plants, but was hardly detectable in leaf and not seen in root tissue. *ARA12* is a calcium-dependent subtilase, whose proteolytic activity is stimulated by the presence of Ca^2+^ ions [[12]](https://paperpile.com/c/slGjxJ/GF4fh).

AT5G22810 is a GDSL esterase/lipase protein (GELP) and candidate genes for suberin biosynthesis and cork regulation [[13]](https://paperpile.com/c/slGjxJ/Ck3Vw). More specifically, some GELPs have been proposed to play a role in suberin monomer (building block) polymerization in *A. thaliana* [[13]](https://paperpile.com/c/slGjxJ/Ck3Vw). The formation of cork-wars (wall thickening and suberin deposition) in the oviposition wounds have been reported during the early stages of development of galls induced by the Cecidomyiidae fly (Diptera) *Parazalepidota clusiae* on *Clusia fluminensis* leaves [[14]](https://paperpile.com/c/slGjxJ/YVXQU). Expression of genes involved in cell wall synthesis, lignin production, suberin deposition, and vascular tissue morphogenesis were also upregulated in the early stage of development of galls induced by the aphid *Schlechtendalia chinensis* on the Chinese sumac, *Rhus javanica* [[15]](https://paperpile.com/c/slGjxJ/AX2Tn). In galls, cell wall reinforcement *via* lignin and/or suberin biosynthesis can be a defense mechanism to prevent the gall-inducing insect from forming a feeding site, such as in the system wheat / Hessian fly *Mayetiola destructor* [[16]](https://paperpile.com/c/slGjxJ/tZSU9). Alternatively, heavy tissue lignification of the outer gall layers can also allow the creation of a protective microenvironment for the benefit of the gall-inducing insect [[17]](https://paperpile.com/c/slGjxJ/EXWFQ).

***1.6 Day 6***

On day 6, we found 73 differentially regulated *Arabidopsis* orthologs, 22 genes (24 loci) were upregulated and 43 genes (49 loci) were downregulated.

***Upregulated DEGs*** *—* Among the upregulated genes, we identified *MDIS1-INTERACTING RECEPTOR LIKE KINASE 2* (*MIK2*, Log2FC = 3.26), *PECTIN METHYL ESTERASE INHIBITOR-PECTIN METHYLESTERASE 25* (*PME25*, Log2FC = 1.52), and *EXPANSIN 1* and *5* (*EXPA1*, Log2FC = 2.71-2.82, 2 loci; *EXPA5*, Log2FC = 2.50).

*MIK2* is a leucine-rich repeat receptor kinase (LRR-RK) that plays a role in response to diverse environmental stresses, including cell wall integrity sensing, and resistance to some plant pathogens [[18]](https://paperpile.com/c/slGjxJ/7YChK) and chewing herbivores [[19]](https://paperpile.com/c/slGjxJ/EV3t0). *MIK2* functions as a pattern recognition receptor (PRRs) that recognizes pathogen- or microbe-associated molecular patterns (PAMPs or MAMPs) or damage-associated molecular patterns (DAMPs) to initiate plant pattern-triggered immunity (PTI) [[19]](https://paperpile.com/c/slGjxJ/EV3t0). *MIK2* has also been identified as an important regulator of responses to cellulose biosynthesis inhibition, as evidenced by the impaired gene expression, JA accumulation and lignin deposition triggered by chemical inhibition of cellulose biosynthesis mutant plants [[20]](https://paperpile.com/c/slGjxJ/PaABx).

The methylesterification status of cell wall pectins is mediated through the interplay of pectin methylesterases (PMEs) and pectin methylesterase inhibitors (PMEIs), and thus, influences the biophysical properties of plant cell walls. PME proteins control pectin methylesterification to regulate cell adhesion, cell wall porosity, and elasticity, as well as stress perception and signaling [[21]](https://paperpile.com/c/slGjxJ/50ISO). The role of *PMEI-PME25* remains unclear. The overexpression of another PMEI (*PMEI-PME2*) led to the development of callus-like structures in presence of exogenous auxin, indicating a role in cell reprogramming during callus formation [[22]](https://paperpile.com/c/slGjxJ/PXPUU).

The modification of cell wall components leads to cell wall loosening or stiffening and can impact undifferentiated cell growth during organ morphogenesis [[23]](https://paperpile.com/c/slGjxJ/dCw9Y). Expansins are cell wall proteins causing wall loosening and irreversible wall extension, a process essential for cell enlargement [[24]](https://paperpile.com/c/slGjxJ/RdMoH). Expansins are present in all plants, and are also involved in softening of ripening fruits and in patterning of floral organs, among other functions. These proteins consist of four sub families: α-expansin (EXPA), β-expansin (EXPB), expansin-like A (EXPLA) and expansin-like B (EXPLB) [[25]](https://paperpile.com/c/slGjxJ/xdnaV). *EXPA1* was upregulated in galls induced by the root-knot nematode *Meloidogyne incognita* on *Populus tremula × P. alba* poplar roots on days 7, 14, and 21 dpi [[26]](https://paperpile.com/c/slGjxJ/Ms2TX). *EXPA1* was also upregulated in galls induced on *A. thaliana* by *M. incognita* at 7, 14, and 21 dpi, together with six other *EXPA* genes (*EXPA6*, *EXPA7*, *EXPA10*, *EXPA11*, *EXPA15*, *EXPA16*) and two *EXPB* genes (*EXPB1*, *EXPB3*) [[27]](https://paperpile.com/c/slGjxJ/k3a5p). *EXPA1* and *EXPB1* were strongly upregulated during syncytium formation of cyst nematode *Heterodera schachtii* on *A. thaliana* roots [[28]](https://paperpile.com/c/slGjxJ/vWxiI).

*EXPA5* was specifically induced in tomato *Lycopersicon esculentum* roots on day 10 dpi upon the potato cyst nematode *Globodera rostochiensis* infection, and on days 4 and 10 dpi upon the root-knot nematode *Meloidogyne javanica* infection [[29–31]](https://paperpile.com/c/slGjxJ/PuAHG+2A4D4+lCh24). In the later system, impaired expression of *EXPA5* reduced parasitism by the nematodes by limiting their ability to establish feeding sites and complete their life cycles [[31]](https://paperpile.com/c/slGjxJ/lCh24).

***Downregulated DEGs*** *—* Among the downregulated genes, we identified *FRAGILE FIBER 8* (*FRA8*, Log2FC = -3.21), *TRICHOME BIREFRINGENCE-LIKE 34* (*TBL34*; Log2FC = -3.35), *CELLULOSE SYNTHASE A4* (*CESA4*; Log2FC = -1.61), *CINNAMYL ALCOHOL DEHYDROGENASE 4* and *5* (*CAD4* or *CAD-C,* Log2FC = -1.70-2.09, 2 loci; *CAD5* or *CAD-D*, Log2FC = 1.91), *UDP-GLUCOSE-DEPENDENT GLUCOSYLTRANSFERASE 72 B1* (*UGT72B1*, Log2FC = -3.88), *PECTIN METHYLESTERASE 1* ​​(*PME1*, Log2FC = 1.59), *S-ADENOSYLMETHIONINE SYNTHETASE 2* (*SAM2*, Log2FC = -1.53), *L-TYPE LECTIN RECEPTOR KINASE VIII.2* (*LECRK-VIII.2*, Log2FC = -0.55), *ATP-BINDING CASSETTE G37* (*ABCG37*, Log2FC = -1.76), *SMALL AUXIN UPREGULATED RNA 64* (*SAUR64*, Log2FC = -5.43), as well as AT1G20030 (Log2FC = -1.60), a pathogenesis-related (PR) thaumatin superfamily protein.

*FRA8* encodes a xylan glucuronyltransferase (GT) involved in the biosynthesis of glucuronoxylan during secondary wall formation [[32]](https://paperpile.com/c/slGjxJ/iv9tf). This GT belonging to the GT family 47 that is expressed during wood formation in poplar shows high sequence identity to *FRA8* (88 % similarity within the pfam03016 domain) [[32]](https://paperpile.com/c/slGjxJ/iv9tf). *FRA8* is expressed specifically in developing vessels and fiber cells [[32]](https://paperpile.com/c/slGjxJ/iv9tf). The stem of *far8* mutants have a drastically reduced xylan and cellulose content, whereas xyloglucan and pectin are elevated [[32]](https://paperpile.com/c/slGjxJ/iv9tf).

*TBL34* is involved in xylan acetylation, an essential for normal secondary wall deposition and plant growth [[33, 34]](https://paperpile.com/c/slGjxJ/yq0KP+rNSMf).

*CESA4* is required for the production of secondary cell wall cellulose taking place after the arrest of cell expansion [[35]](https://paperpile.com/c/slGjxJ/KqGQM).

The lignin biosynthesis-specific CAD enzymes act downstream of phenylpropanoid biosynthetic enzymes and catalyze the biosynthesis of specific monolignols [[36, 37]](https://paperpile.com/c/slGjxJ/ONLoY+Z8B5d). Lignification renders the cell wall more resistant to pathogen attack [[38]](https://paperpile.com/c/slGjxJ/YQqiK). *CAD4* and *CAD5* act as the primary genes involved in lignin biosynthesis in the floral stem of *A. thaliana* by supplying both coniferyl and sinapyl alcohols for monolignol biosynthesis [[37]](https://paperpile.com/c/slGjxJ/Z8B5d). In *cad4 cda5* double mutants, lignin content was reduced, ferulic acid and sinapic acid accumulated, while coniferyl alcohol and sinapyl alcohol decreased [[37]](https://paperpile.com/c/slGjxJ/Z8B5d).

*UGT72B1* is essential for the normal cell wall lignification in *A. thaliana* [[39]](https://paperpile.com/c/slGjxJ/HCHag). *UGT72B1* catalyzes the glucose conjugation of monolignols, especially coniferyl alcohol and coniferyl aldehyde [[40]](https://paperpile.com/c/slGjxJ/LnK6x). The expression of *UGT72B1* can be induced by both SA and JA [[41]](https://paperpile.com/c/slGjxJ/4e4aP). Additionally, *ugt72b1* mutant showed aggravated and ectopic lignification in floral stems along with arrested growth and anthocyanin accumulation [[39]](https://paperpile.com/c/slGjxJ/HCHag).

Pectin is synthesized in a highly esterified form and then de-esterified by pectin methylesterases (PMEs). The regulation of PME activity can change the biomechanical properties of organs, and thus the driving forces for cell growth by expansion [[42]](https://paperpile.com/c/slGjxJ/qNnJl). PMEs participate in organ initiation *via* changes in the viscoelastic properties of meristem cells (mechanical signal) [[42]](https://paperpile.com/c/slGjxJ/qNnJl). PMEs are also directly involved in plant defense response to pathogens by impacting pectin, the main component of plant cell walls. *PME1* was identified as a fruit-development-related gene [[43]](https://paperpile.com/c/slGjxJ/Vvv6o). Strawberry plants (*Fragaria* × *ananassa*) overexpressing *PME1* have an increased resistance to some pathogens [[44]](https://paperpile.com/c/slGjxJ/RZZXC). Herbivore attack by *Manduca sexta* larvae increased oral-secretion (OS)-inducible PME expression and activity, as well as methanol emissions [[45]](https://paperpile.com/c/slGjxJ/7AQkU). Additionally, the lack of *PME1* in tobacco (*Nicotiana attenuata*) led to an increased performance of *M. sexta* caterpillars [[45]](https://paperpile.com/c/slGjxJ/7AQkU). OS-induced jasmonic acid (JA) and salicylic acid (SA) burst were significantly reduced in the mutant plants [[45]](https://paperpile.com/c/slGjxJ/7AQkU).

SAM is a methyl donor for methylation reactions and participates in an early step in ethylene (ET) production [[46]](https://paperpile.com/c/slGjxJ/mXnC4). The expression of *SAM2* increases in response to wounding [[47]](https://paperpile.com/c/slGjxJ/jXOvt).

*LECRK-VIII.2* acts upstream of the mitogen-activated protein kinase (MAPK) gene *MPK6*, and is involved in mediating the signaling crosstalk among sucrose, glucose and brassinosteroids [[48]](https://paperpile.com/c/slGjxJ/PAVUE).

In *A. thaliana*, *ABCG37* acts as a broad substrate specificity exporter for endogenous auxin precursor IBA, but not IAA, and is involved in regulating auxin homeostasis and plant development [[49]](https://paperpile.com/c/slGjxJ/e9zk2).

*SMALL AUXIN UPREGULATED RNA* (*SAUR*) is a major auxin-inducible gene class, with some *SAUR* genes inducing cell elongation. *SAUR64* is specifically upregulated during the flower opening process (from flower buds to flower starting opening) [[50]](https://paperpile.com/c/slGjxJ/MntVF), and is also involved in the fruit size regulation [[51]](https://paperpile.com/c/slGjxJ/jTex8). *SAUR64*, along with 9 other *SAUR* genes (*SAUR1*, *SAUR5*, *SAUR14*, *SAUR45*, *SAUR61*, *SAUR66*, *SAUR67*/*68*, *SAUR72*, *SAUR74*), was reported specifically present in the galls induced by the psyllid *Metrosideros polymorpha* on *Eucalyptus grandis*, but absent from control tissues [[52]](https://paperpile.com/c/slGjxJ/3cRuJ).

***1.7 Day 7***

On day 7, we found 51 differentially regulated *Arabidopsis* orthologs, 10 genes (10 loci) were upregulated and 37 genes (41 loci) were downregulated.

***Upregulated DEGs*** *—* Among the upregulated genes, we identified *RPL1* (Log2FC = 3.71), and *BETA-GALACTOSIDASE 3* (*BGAL3*, Log2FC = 1.32).

*BGAL3* is involved in plant secondary cell wall biogenesis, composition, strength, and extensibility [[53]](https://paperpile.com/c/slGjxJ/MEsxx). *BGAL3* is upregulated by cytokinins (CKs) [[53]](https://paperpile.com/c/slGjxJ/MEsxx).

***Downregulated DEGs*** *—* Among the downregulated genes, we identified *COBRA* (*COB*, Log2FC = -2.31), *TRICHOME BIREFRINGENCE-LIKE 33* (*TBL33*, Log2FC = -2.72), *TBL34* (Log2FC = -3.10), *CESA4* (Log2FC = -3.05-3.48, 2 loci), *CESA 7* (Log2FC = -2.95), *IRX15-L* (Log2FC = -2.59), *UGT72B1* (Log2FC = -4.15), *CAD4* (Log2FC = -2.70), *CAD5* (Log2FC = -1.85), and *SVB3* (Log2FC = -4.41).

*CESA7* is an essential component of the secondary cell wall cellulose synthase complex [[54]](https://paperpile.com/c/slGjxJ/i4Wby).

*COB* encodes ​​glycosylphosphatidylinositol (GPI)-anchored protein that modulates cellulose deposition and oriented cell expansion [[55]](https://paperpile.com/c/slGjxJ/ABwCV).

*IRX15-L* affects xylan biosynthesis in secondary cell walls, resulting in a decrease in xylan content and an increase in the degree of methylation [[56, 57]](https://paperpile.com/c/slGjxJ/DeYtR+0P3tN).

*TBL33* is involved in xylan acetylation, an essential for normal secondary wall deposition and plant growth [[33, 34]](https://paperpile.com/c/slGjxJ/yq0KP+rNSMf).

*SVB3* is involved in ABA-mediated regulation of trichome formation [[58]](https://paperpile.com/c/slGjxJ/tcRzI).

***2. DEGs unique to the resistant genotype***

***2.1 Day 1***

On day 1, we found 114 differentially regulated *Arabidopsis* orthologs, 65 genes (78 loci) were upregulated and 28 genes (36 loci) were downregulated.

***Upregulated DEGs*** *—* Among the upregulated genes, we identified ​​*MIK2* (Log2FC = 4.41), *PBS1-LIKE 21* (*PBL21*; Log2FC = 0.95), *WRKY DNA-BINDING PROTEIN 18* and *40* (*WRKY18*, Log2FC = 4.30; *WRKY40*, Log2FC = 3.29), *EARLY-RESPONSIVE TO DEHYDRATION* 9 (*ERD9*, Log2FC = 4.04), *HEAVY METAL ASSOCIATED PROTEIN 9* (*HMAD1* or *HMP09*, Log2FC = 3.94, 4.03, 4.95, 3 loci), *NUCLEOREDOXIN 1* (*NRX1*, Log2FC = 2.68-2.70, 2 loci), *XERICO* (*XER*, Log2FC = 3.04), *S-ADENOSYLMETHIONINE DECARBOXYLASE* (*SAMDC*, Log2FC = 2.86), *L-TYPE LECTIN RECEPTOR KINASE S.5* (*LECRK-S.5*, Log2FC = 3.30), *ENHANCED DISEASE SUSCEPTIBILITY 1* (*EDS1*, Log2FC = 2.03), *CAP-DERIVED PEPTIDE 1* (*CAPE1*, Log2FC = 4.15), *CYSTEINE SYNTHASE C1* (*CYSC1*, Log2FC = 4.26), *TRANSPARENT TESTA 7* / *CYTOCHROME P450, FAMILY 75, SUBFAMILY B, POLYPEPTIDE 1* (*TT7* or *CYP75B1*, Log2FC = 3.67), *GLUTATHIONE S-TRANSFERASE TAU 18* (*GSTU18* or *GST29*, Log2FC = 3.36), *UDP-GLUCOSE-DEPENDENT GLYCOSYLTRANSFERASE 72B1*, *73B4*, and *74F1* (*GT72B1* / *UGT72B1*, Log2FC = 2.76; *UGT73B4*, Log2FC = 6.65; *UGT74F1*, Log2FC = 6.80), *GLUTATHIONE S-TRANSFERASE TAU 8* and *PHI 9* (*GSTU8*, Log2FC = 2.97; *GSTF9*, Log2FC = 3.29), *ONE-HELIX PROTEIN 2* (*OHP2*, Log2FC = 2.81), *S-METHYL-5-THIORIBOSE KINASE* (*MTK1*, Log2FC = 2.42-2.69, 2 loci), *NAC DOMAIN CONTAINING PROTEIN 103* (*NAC103*, Log2FC = 5.87), *3-KETOACYL-COA SYNTHASE 6* (*KCS6*, Log2FC = 4.16), *RAFFINOSE SYNTHASE 2* (*RS2* or *SIP2*, Log2FC = 2.37), as well as two unknown pathogenesis-related thaumatin superfamily proteins involved in plant defense (AT4G38660, Log2FC = 5.97; AT1G20030, Log2FC = 6.33), a CAP (Cysteine-rich secretory proteins, Antigen 5, and Pathogenesis-related 1 protein) superfamily protein (AT1G50050, Log2FC = 3.70), and a NADH-ubiquinone oxidoreductase chain protein (AT1G76185, Log2FC = 3.30).

*PBL21* is a plant receptor-like cytoplasmic kinase belonging to family VII (RLCK VII) [[59]](https://paperpile.com/c/slGjxJ/OTxRf). The orthologue of *PBL21* in rice (*OsRLCK176*) is part of chitin-mediated MAPK activation [[60]](https://paperpile.com/c/slGjxJ/iB7so). *PBL21* is involved in *Pseudomonas syringae* effector-​​triggered immune response (ETI) that limits bacterial proliferation *via* HR in *A. thaliana* [[59]](https://paperpile.com/c/slGjxJ/OTxRf). Additionally, *PBL21* is also involved in the resistant phenotype observed in response to *Xanthomonas campestris* effector protein AvrAC/XopAC after wound-inoculation of *A. thaliana* [[61]](https://paperpile.com/c/slGjxJ/RIbTP).

AT3G58640 is involved in MAPKKK-like protein phosphorylation. This gene was downregulated in susceptible *Nicotiana attenuata* mutant plants in response to *Manduca sexta* larvae oral secretion elicitors [[62]](https://paperpile.com/c/slGjxJ/ygiwG).

The transcription factor *WRKY40* is related to PAMP-triggered immunity [[15]](https://paperpile.com/c/slGjxJ/AX2Tn), and is induced by the green leaf volatile E-2-hexenal produced upon wounding, herbivory, or pathogen infection [[63]](https://paperpile.com/c/slGjxJ/5CIux). *WRKY40* was more upregulated in the resistant cultivar of *Gossypium hirsutum* cotton than in the susceptible cultivar in response to *Bemisia tabaci* whitefly infestation [[64]](https://paperpile.com/c/slGjxJ/qj8BU). *A. thaliana* *wrky40* mutants were more susceptible to *Frankliniella occidentalis* thrips feeding damage and oviposition than the wildtype plants [[65]](https://paperpile.com/c/slGjxJ/9wKRc). *WRKY40* was also induced during *A. thaliana* resistance to *Spodoptera littoralis* and *S. exigua* infestation [[66, 67]](https://paperpile.com/c/slGjxJ/AMdDU+PCKwW). *WRKY40* was downregulated in *Solanum tuberosum* potato plants during a compatible interaction with *Meloidogyne javanica* root-knot nematodes [[68, 69]](https://paperpile.com/c/slGjxJ/MXOFa+ILvP). Additionally, *WRKY40* was highly expressed during the early gall induction process by the aphid *Schlechtendalia chinensi*s in the sumac *Rhus javanica* [[15]](https://paperpile.com/c/slGjxJ/AX2Tn).

*ERD9* is part of the plant’s core environmental stress response. *ERD9*, involved in glutathione metabolic process, was induced by *Myzus persicae* feeding at 6 hpi on *A. thaliana* [[70]](https://paperpile.com/c/slGjxJ/1qv28).

*HMAD1* (or *HMP09*) is a regulator of plant immunity [[71]](https://paperpile.com/c/slGjxJ/y63Kf). In *hmad1* mutant *A. thaliana* plants, inoculation with an avirulent strain of *Pseudomonas syringae* led to a negative regulation of R-gene mediated resistance (HR and cell death) [[71]](https://paperpile.com/c/slGjxJ/y63Kf).

Simultaneous ROS production and scavenging by pathogen-induced *NRX1* is key for the correct orchestration of plant immune cascades in response to the fungus *Verticillium dahliae* in cotton *Gossypium barbadense* [[72]](https://paperpile.com/c/slGjxJ/SmYVt). In the absence of *NRX1*, ROS accumulated and resistance was reduced [[72]](https://paperpile.com/c/slGjxJ/SmYVt). This gene was upregulated in response to galls induced by the root-knot nematode *Meloidogyne incognita* during the early stages of *A. thaliana* parasitism [[73]](https://paperpile.com/c/slGjxJ/bO1nD).

*XERICO* was upregulated in response to *Brevicoryne brassicae* aphid feeding on *A. thaliana* [[74]](https://paperpile.com/c/slGjxJ/fI1rP).

*SAMDC* is a key enzyme in polyamine (spermidine and spermine) biosynthesis [[75]](https://paperpile.com/c/slGjxJ/oeyvx). Polyamine catabolism produces H_2_O_2_, which plays a role in plant defense by contributing to the HR that acts against different biotic stressors. During an incompatible interaction between barley (*Hordeum vulgare*) and powdery mildew fungus (*Blumeria graminis*), levels of free and conjugated spermidine, as well as SAMDC enzyme activity, increased [[76]](https://paperpile.com/c/slGjxJ/0wECX). In contrast, spermidine content was strongly decreased in successful galls induced by *Cynips quercusfolii*, *Neuroterus numismalis*, and *Neuroterus quercusbaccarum* on *Quercus robur* oak, when compared with controls [[77]](https://paperpile.com/c/slGjxJ/nE3bm). Spermine level was also strongly reduced in successful galls induced by *N. numismalis* [[77]](https://paperpile.com/c/slGjxJ/nE3bm). Polyamines accumulated during *Rhopalosiphum padi* aphid infestation in resistant cultivar of triticale (*Triticosecale*; hybrid of wheat and rye) [[78]](https://paperpile.com/c/slGjxJ/gnNQ1). *R. padi* aphid feeding behavior was disturbed by polyamine, suggesting a role of polyamines in plant disease resistance [[79]](https://paperpile.com/c/slGjxJ/LPwdz).

*LECRK-S.5* positively regulates plant immunity at the transcriptional level and mediates broad-spectrum resistance *via* the regulation of priming [[80]](https://paperpile.com/c/slGjxJ/kYFXp). In the absence of *LECRK-S.5* expression, the pepper *Capsicum annuum* displayed an enhanced susceptibility to several viruses (pepper mild mottle virus, *Xanthomonas campestris*, *Phytophthora capsici*, and tobacco mosaic virus) with reduced HR, ROS burst, MAPK activation, and secondary metabolite biosynthesis [[80]](https://paperpile.com/c/slGjxJ/kYFXp). *LECRK-S.5* was upregulated in *A. thaliana* at the migratory stage of infection by the sugarbeet cyst nematode, *Heterodera schachtii*, as well as upon plant exposure to nematode elicitors [[81]](https://paperpile.com/c/slGjxJ/Lf9Fu).

Pathogen-induced *EDS1* controls defense activation and programmed cell death *via* SA accumulation [[82]](https://paperpile.com/c/slGjxJ/2EZGK). *EDS1* is needed for basal resistance to invasive pathogens by restricting disease progression. *EDS1* was upregulated in rice (*Oryza sativa*) during resistance against the brown planthopper *Nilaparvata lugens* [[83]](https://paperpile.com/c/slGjxJ/YeCJN). *Pieris brassicae* egg-induced *PR1* expression in *A. thaliana* is dependent on ROS accumulation which requires *EDS1* and *NPR1* activity, as well as activation of the SA pathway [[84–86]](https://paperpile.com/c/slGjxJ/8G0mD+yAwYI+cgG3M).

*CAPE1* is derived from *PR1*. *CAPE1* acts as an elicitor for PAMP/MAMP/DAMP, as well as an inducer of H_2_O_2_ in response to wounding, insect attacks, and pathogen infections, thus leading to the activation of defense cascades [[87]](https://paperpile.com/c/slGjxJ/E84Gy).

AT1G50050 is part of the pathogenesis-related 1 superfamily.

The gene AT1G20030, a pathogenesis-related thaumatin superfamily protein, was in the top 25 upregulated genes in *Ulmus minor* elms during invasion by the fungus *Ophiostoma novo-ulmi* [[88]](https://paperpile.com/c/slGjxJ/9WX2Z).

*CYSC1* was identified in *A. thaliana* as a group of protein interactors involved in cell death and HR, linking glucosinolate defense compounds and defense against biotrophic pathogens [[89]](https://paperpile.com/c/slGjxJ/6XNaw). *CYSC1* is involved in activation of defense genes and enhanced resistance against the pathogenic bacteria *Pseudomonas syringae* [[90]](https://paperpile.com/c/slGjxJ/Ph0Gq)*.*

*TT7* (or *CYP75B1*) is a flavonoid 3’-hydroxylase (F3H) involved in the phenylpropanoid/flavonoid biosynthesis. *TT7* was upregulated in the spruce *Picea sitchensis* in response to mechanical wounding, as well as upon the white pine weevil *Pissodes strobi* and spruce budworm *Choristoneura occidentalis* herbivory [[91]](https://paperpile.com/c/slGjxJ/sMgni). *TT7* was also upregulated in Indian mustard *Brassica juncea* during unsuccessful infestation by the cowpea aphid *Aphis craccivora* [[92]](https://paperpile.com/c/slGjxJ/6Fx4o). *TT7* was induced by *Helicoverpa armigera* caterpillars feeding in cotton *Gossypium hirsutum* [[93]](https://paperpile.com/c/slGjxJ/tPaZO), and by the pine weevil *Hylobius abietis* in the pine *Pinus sylvestris* [[94]](https://paperpile.com/c/slGjxJ/KC3AB).

AT1G76185 was induced in *A. thaliana* during the oxidative burst in response to the virulent bacterial strain *Pseudomonas syringae* [[95]](https://paperpile.com/c/slGjxJ/kqJpv).

*WRKY18* is a positive regulator of effector-triggered immunity (ETI [[96]](https://paperpile.com/c/slGjxJ/dxoDZ)) and defense responses [[97]](https://paperpile.com/c/slGjxJ/z8uib), and is involved in ABA signaling. The overexpression of *WRKY18* in *A. thaliana* led to an increased resistance against the bacteria *Pseudomonas syringae* and the caterpillar *Spodoptera littoralis* [[66, 96, 98]](https://paperpile.com/c/slGjxJ/dxoDZ+KHrVL+AMdDU). *WRKY18* is involved in the isoflavonoid phytoalexin(s) biosynthesis [[99]](https://paperpile.com/c/slGjxJ/r6M8F). Isoflavonoid phytoalexins displayed feeding-deterrent properties against the specialist Mexican bean beetle, *Epilachna varivestis*, by reducing the *Glycine max* soybean plant digestibility [[100]](https://paperpile.com/c/slGjxJ/ghBtQ). ​​An isoflavonoid phytoalexin from *Lotus pedunculatus* was identified as a major feeding deterrent for the grass grub beetle *Costelytra zealandica* [[101]](https://paperpile.com/c/slGjxJ/OJ2TR). Similar results have been demonstrated in soybean *Glycine max*, with an accumulation of isoflavonoid phytoalexins at the aphid *Aphis glycines* feeding site, deterring feeding before the aphids reach the phloem [[102]](https://paperpile.com/c/slGjxJ/XpxQC).

Numerous plant glutathione transferases (GSTs) participate in the binding and transport of secondary metabolites, such as flavonoids including anthocyanins, and polyphenols [[103]](https://paperpile.com/c/slGjxJ/mhZgg).​​ *GSTU18* (or *GST29*) is a serine-GST involved in the catalyzation of glutathione conjugation and has a peroxidase activity [[103]](https://paperpile.com/c/slGjxJ/mhZgg). *GSTU18* is part of the lipid stress response and is phytoprostane-responsive [[103]](https://paperpile.com/c/slGjxJ/mhZgg). *GSTU18* was downregulated in *A. thaliana* in response to infestation by the aphids *Myzus persicae* or *Brevicoryne brassicae* [[104]](https://paperpile.com/c/slGjxJ/kHe9K), as well as in corn (*Zea mays*) in response to colonization by the aphids *Rhopalosiphum padi* or *Sitobion avenae* [[105]](https://paperpile.com/c/slGjxJ/SdktC).

*UGT72B1* (or *GT72B1*) catalyzes the production of the phenolic antioxidant tyrosol, which showed a strong dose-dependent inhibitory effect against several plant pathogens [[106]](https://paperpile.com/c/slGjxJ/kLbir).

*UGT73B4* belongs to the UGT group D which preferably glycosylates the pathogen-responsive phytohormone salicylic acid (SA) [[107]](https://paperpile.com/c/slGjxJ/gdUHo). *UGT73B4* was upregulated in *A. thaliana* in response to several pathogens, including *Alternaria brassiciola*, *Blumeria graminis*, *Escherichia coli*, *Golovinomyces cichoracearum*, *Golovinomyces orontii*, *Hyaloperonospora arabidopsidis*, *Plectospherella cuccumerina*, *Pseudomonas syringae*, *Rhizoctonia solani*, and *Xanthomonas campestri* [[107]](https://paperpile.com/c/slGjxJ/gdUHo). *UGT73B4* was also upregulated in *A. thaliana* in response to the gall-inducing bacteria, *Agrobacterium tumefaciens* [[108]](https://paperpile.com/c/slGjxJ/GA6B0). Additionally, *UGT73B4* was induced in *A. thaliana* in response to egg oviposition by *Pieris brassicae* butterflies, or to treatment with egg extract only [[109]](https://paperpile.com/c/slGjxJ/CcHt6). The induction of SA and H_2_O_2_ accumulation, defense gene expression, and cell death, all part of PTI, was observed in response to phosphatidylcholines, as egg-associated molecular patterns (EAMPs) [[109]](https://paperpile.com/c/slGjxJ/CcHt6).

*UGT74F1* transfers glucose from UDP-glucose to SA to form salicylic acid glucoside (SAG) [[110, 111]](https://paperpile.com/c/slGjxJ/Gfjja+zoZXB). *UGT74F1* can also form other products: anthranilate-glucose ester [[112]](https://paperpile.com/c/slGjxJ/mtZzd), benzoic acid glucose ester and 4-hydroxybenzoic acid glucose ester [[113]](https://paperpile.com/c/slGjxJ/vcijh), and multiple quercetin glucosides [[114]](https://paperpile.com/c/slGjxJ/mFxLO).

*GSTU8* was upregulated in *A. thaliana* in response to *Pieris brassicae* oviposition [[115]](https://paperpile.com/c/slGjxJ/dxAiy).

*GSTF9* is a key glutathione S-transferase (GST) in indole glucosinolate biosynthesis [[19, 116]](https://paperpile.com/c/slGjxJ/fX4x4+EV3t0). The expression of *GSTF9* was uniquely enhanced by inoculation with *Fusarium sporotrichioides* in *A. thaliana* [[117]](https://paperpile.com/c/slGjxJ/KwWiu). Additionally, *GSTF9* transcripts were upregulated in response to *Spodoptera littoralis* infestation [[19]](https://paperpile.com/c/slGjxJ/EV3t0).

*OHP2* was upregulated in *Solanum lycopersicum* tomato plants inoculated with pepper yellow mosaic virus [[118]](https://paperpile.com/c/slGjxJ/3kHLx). Moreover, *OHP2* was differentially upregulated after *Pieris brassicae* oviposition on *Brassica oleracea* Brussels sprout plants [[119]](https://paperpile.com/c/slGjxJ/8x2uq).

*MTK1* is a major mediator of environmental stress signal transduction [[120]](https://paperpile.com/c/slGjxJ/OsBOX). Overexpression of *MTK1* led to cell apoptosis [[120]](https://paperpile.com/c/slGjxJ/OsBOX).

*NAC103* from canola *Brassica napus* induces ROS accumulation and cell death in response to multiple signals, including SA and fungus *Sclerotinia sclerotiorum* [[121]](https://paperpile.com/c/slGjxJ/bGdDI).​​

*KCS6*/*CER6* is a ketoacyl CoA synthase involved in very long chain fatty acid elongation during wax biosynthesis [[122]](https://paperpile.com/c/slGjxJ/bZj0E). *kcs6* mutant barley plants (*Hordeum vulgare*) displayed a 80 % reduced coverage of the cuticle with epicular waxes [[123]](https://paperpile.com/c/slGjxJ/QXcax). *KCS6* was upregulated during incompatible interactions between the hessian fly *Mayetiola destructor* and resistant *Triticum aestivum* wheat plants, leading to a change in wax composition at the feeding site [[122]](https://paperpile.com/c/slGjxJ/bZj0E).

*RS2* (or *SIP2*) catalyzes the conversion of sucrose (glucose-fructose) + galactinol into raffinose (sucrose-galactose). Another raffinose synthase gene (*RS5*) was strongly downregulated in phylloxera *Daktulosphaira vitifoliae* galls on susceptible grapevine *Vitis riparia* leaves [[124]](https://paperpile.com/c/slGjxJ/9TU9d). Resistant *A. thaliana* mutant plants accumulated raffinose at the feeding site during the resistance process against the green peach aphid *Myzus persicae* [[125]](https://paperpile.com/c/slGjxJ/6slKf). Under herbivore attack, plant’s primary metabolites can be redirected towards undamaged storage organs, such reallocation of plant nutrients is preceded by a local increase in soluble forms (such as the non-reducing sugar, raffinose) in preparation for phloem transport. The decrease of nutrients at the site of attack has been reported as an anti-herbivore mechanism [[126, 127]](https://paperpile.com/c/slGjxJ/sbGoP+8f6Vu).

***Downregulated DEGs*** *—* Among the downregulated genes, we identified *GXM1* (Log2FC = -2.59), *ARA12* (or *SBT1.7*, Log2FC = -1.76), *HISTONE THREE RELATED 15* (*H3.15*, Log2FC = -2.09), *BETA-1,3-GLUCANASE_PUTATIVE* (*BG_PPAP*, Log2FC = -2.46), *ARABINOGALACTAN PROTEIN 9* and *13* (*AGP9*, Log2FC = -2.03; *AGP13*, Log2FC = -1.26-1.52, 2 loci), as well as AT4G13210 (Log2FC = -2.65), a pectate lyase.

*H3.15* plays a key role in cell fate reprogramming to produce pluripotent callus cells upon wounding in *A. thaliana* [[128]](https://paperpile.com/c/slGjxJ/WySrm). Overexpression of *H3.15* promotes cell proliferation to form a larger callus at the wounding site, which results in the transcriptional de-repression of downstream genes, such as *WUSCHEL RELATED HOMEOBOX 11* (*WOX11*) as part of cell fate transitioning during *de novo* organogenesis [[128]](https://paperpile.com/c/slGjxJ/WySrm). In contrast, *htr15* mutation inhibits callus formation [[128]](https://paperpile.com/c/slGjxJ/WySrm).

*BG_PPAP*, a β-1,3-glucanase, is a plasmodesmata-associated membrane protein involved in plasmodesmal callose degradation [[129, 130]](https://paperpile.com/c/slGjxJ/cbppl+hTdCw). Callose is deposited in plasmodesmata to reduce cell-to-cell traffic upon pathogen perception, and limit the spread of the pathogen [[129, 130]](https://paperpile.com/c/slGjxJ/cbppl+hTdCw).

*AGP13* is involved in cell wall formation and remodeling [[131]](https://paperpile.com/c/slGjxJ/6F9rp).

*AGP9* was downregulated during activation of systemic acquired resistance (SAR) in *A. thaliana* in response to *Pseudomonas syringae* [[132]](https://paperpile.com/c/slGjxJ/fagsN) or *Brevicoryne brassicae* aphid feeding [[74]](https://paperpile.com/c/slGjxJ/fI1rP).

AT4G13210 was upregulated in *A. thaliana* in successful *Agrobacterium tumefaciens* galls [[133]](https://paperpile.com/c/slGjxJ/hakCx).

***2.2 Day 2***

On day 2, we found 11 differentially regulated *Arabidopsis* orthologs, 9 genes (9 loci) were upregulated and 2 genes (2 loci) were downregulated.

***Upregulated DEGs*** *—* Among the upregulated genes, we identified *GSTU18* (Log2FC = 4.86), *UGT74F1* (Log2FC = 5.91), *UGT73B4* (Log2FC = 5.10), as well as AT4G38660 (Log2FC = 4.42), a pathogenesis-related thaumatin superfamily protein involved in plant defense.

***2.3 Day 3***

On day 3, we found 4 differentially regulated *Arabidopsis* orthologs, all 4 genes (4 loci) were upregulated.

***Upregulated DEGs*** *—* Among the upregulated genes, we identified *SERINE/THREONINE-PROTEIN KINASE 1* (*PBS1*)*-LIKE 1* (*PBL1*, Log2FC = 2.53).

*PBL1* is a receptor-like cytoplasmic kinase (RLCK) required for PAMP/MAMP/DAMP-induced immunity (PTI), including early signaling events, Ca^2+^ signaling and defense responses downstream of the Leucine-rich repeat transmembrane receptor kinase *FLAGELLIN SENSITIVE 2* (*FLS2*) [[134, 135]](https://paperpile.com/c/slGjxJ/mulEp+89nY1). *FLS2* is essential for bacterial flagellin (flg22) perception, and involved in oxidative burst, ROS production, and callose deposition in plasmodesmata, leading to reduced flg22-mediated growth inhibition [[134–136]](https://paperpile.com/c/slGjxJ/mulEp+89nY1+KBmUj). *PBL1* integrates immune signaling from multiple immune receptors and *pbl1* mutants displayed a reduction in flg22-induced H_2_O_2_ production [[137]](https://paperpile.com/c/slGjxJ/ER1Hk). The crucifer anthracnose fungus *Colletotrichum higginsianum* was more virulent on *A. thaliana* mutant plants [[138]](https://paperpile.com/c/slGjxJ/PmCPR).

***2.4 Day 4***

On day 4, we found 25 differentially regulated *Arabidopsis* orthologs, all 20 genes (25 loci) were upregulated.

***Upregulated DEGs*** *—* Among the upregulated genes, we identified *PBL21* (Log2FC = 3.72-3.87, 2 loci), *LECRK-S.5* (Log2FC = 1.43), *UGT73B4* (Log2FC = 4.19), *AGAMOUS-LIKE 17* (*AGL17*, Log2FC = 3.84), *L-GALACTONO-1,4-LACTONE DEHYDROGENASE* (*GLDH*, Log2FC = 3.53), *NAC DOMAIN CONTAINING PROTEIN 82* / *VND-INTERACTING 1* (*NAC082* or *VNI1*, Log2FC = 5.12), *ECHIDNA* (*ECH*, Log2FC = 5.88), as well as two pathogenesis-related thaumatin superfamily proteins (AT1G20030, Log2FC = 8.11; AT4G38660, Log2FC = 4.14), and an alpha/beta-hydrolases superfamily protein (AT5G65400, Log2FC = 5.70).

AT5G65400 was upregulated in the pine *Pinus sylvestris* after mechanical wounding [[139]](https://paperpile.com/c/slGjxJ/TYAgB).

*AGL17*, a transcription factor target of *WRKY33*, was upregulated in *A. thaliana* as part of the transcriptional network associated with defense during resistance against the fungus *Botrytis cinerea* [[140]](https://paperpile.com/c/slGjxJ/1Je39). *AGL17* was upregulated in canola *Brassica napus* following inoculation by the fungus *Alternaria brassicicola* [[141]](https://paperpile.com/c/slGjxJ/DDCDr).

*GLDH* is involved in L-ascorbate biosynthesis [[142]](https://paperpile.com/c/slGjxJ/LoH9B). Ascorbic acid is an antioxidant that plays a key role in scavenging ROS to protect the plant from cellular cytotoxicity [[142]](https://paperpile.com/c/slGjxJ/LoH9B). *GLDH* was upregulated during incompatible interaction between *A. thaliana* and bacterial elicitors from *Pseudomonas syringae* [[142]](https://paperpile.com/c/slGjxJ/LoH9B).

AT4G38660 belongs to the PR5 family, and is induced by salicylic acid [[143]](https://paperpile.com/c/slGjxJ/DBBj1) and wounding [[144]](https://paperpile.com/c/slGjxJ/z4u2X). This gene was downregulated in *A. thaliana* during compatible interaction with the gall-inducing bacteria *Agrobacterium tumefaciens* [[145]](https://paperpile.com/c/slGjxJ/YGZza).

*NAC103* is the closest homolog to *VNI1* at the molecular level, both promoting the differentiation of various types of cells *via* the modulation of multiple NAC domain transcription factors [[146]](https://paperpile.com/c/slGjxJ/cuCi5). In canola *Brassica napus* and tobacco relative *Nicotiana benthamiana*, *NAC103* transcription factor induces ROS accumulation and cell death during HR against biotic stress, such as inoculation by the fungus *Sclerotinia sclerotiorum* [[121]](https://paperpile.com/c/slGjxJ/bGdDI). *NAC103* is a wound- and ABA-inducible negative regulator of aliphatic suberin and wax accumulation [232].

*ECH* is required for flavonoid accumulation in *A. thaliana* [[147]](https://paperpile.com/c/slGjxJ/gXoRH).

*NAC082* (or *VNI1*) is a ribosomal stress response marker [[148]](https://paperpile.com/c/slGjxJ/OlR9C), and is a NAC transcription factor involved in flavonoid biosynthesis [[149, 150]](https://paperpile.com/c/slGjxJ/Fx65G+0wISJ). *NAC082* was highly upregulated in the tobacco relative *Nicotiana benthamiana* in response to the fungus *Phytophthora infestans* effectors, leading to enhanced pathogenicity, ribosome malfunction, and cell death [[148]](https://paperpile.com/c/slGjxJ/OlR9C). Additionally, this NAC gene interacts with *VASCULAR-RELATED NAC-DOMAIN PROTEIN 7* (*VND7*), a repressor of xylem vessel development and differentiation [[146, 151]](https://paperpile.com/c/slGjxJ/RLl3Y+cuCi5).

***2.5 Day 5***

On day 5, we found 66 differentially regulated *Arabidopsis* orthologs, 44 genes (61 loci) were upregulated and 5 genes (5 loci) were downregulated.

***Upregulated DEGs*** *—* Among the upregulated genes, we identified *RLP1* (Log2FC = 2.70, 3.13, 5.74, 5.95, 4 loci), *PBL21* (Log2FC = 3.55-3.62, 2 loci), *ECH* (Log2FC = 5.17), *BGAL3* (Log2FC = 1.52), *CDC26* (Log2FC = 4.19), *UGT73B4* (Log2FC = 5.55), *NAC082* (or *VNI1*, Log2FC = 3.04), *MIK2* (Log2FC = 1.63, 1.95, 2.90, 2.95, 4.22, 4.29, 4.91, 5.30, 8 loci), *OSM34* (Log2FC = 5.77), *RECEPTOR-LIKE KINASE 7* (*RLK7* or *LRR XI-23*, Log2FC = 4.29), *LEAF RUST 10 DISEASE-RESISTANCE LOCUS RECEPTOR- LIKE PROTEIN KINASE-LIKE 1.2* (*LRK10L1.2*, Log2FC = 2.99), *LUTEIN DEFICIENT 1* (*LUT1* or *CYP97C1*, Log2FC = 1.48), *PBS1-LIKE 36* (*PBL36*, Log2FC = 2.65), *OLIGOGALACTURONIDE OXIDASE 1* (*OGOX1* or *BBE19*, Log2FC = 1.40), *INDOLE-3-BUTYRIC ACID RESPONSE 10* (*IBR10*, Log2FC = 1.63), *EXTENSIN 3* (*EXT3*, Log2FC = 2.99), as well as an alpha/beta-hydrolases superfamily protein (AT5G65400, Log2FC = 4.92), a pathogenesis-related thaumatin superfamily proteins involved in plant defense (AT4G38660, Log2FC = 6.72), and an exostosin family protein (AT5G11610, Log2FC = 6.20).

*RLK7* (or *LRR XI-23*) was upregulated in response to wounding in both local and systemic tissue in *A. thaliana* [[152]](https://paperpile.com/c/slGjxJ/3lVZc). *RLK7* is required for PIP1- (PIP, PAMP-Induced Peptides) and PIP2-elicited immune activation, and plays a crucial role in PTI amplification, leading to enhanced resistance of *A. thaliana* against the bacteria *Pseudomonas syringae* and fungus *Fusarium oxysporum* [[153]](https://paperpile.com/c/slGjxJ/pxfGU). *RLK7* binds PIP1 (PAMP-induced secreted peptides), inducing the reinforcement of immune signaling *via* the induction of stomatal closure, callose deposition, MAPK activation (*MPK3* and *MPK6*), Ca^2+^ signaling, and production of ROS [[153, 154]](https://paperpile.com/c/slGjxJ/pxfGU+YSrra).

*LRK10L1.2* is a RLK involved in ABA signaling during oxidative stress [[155]](https://paperpile.com/c/slGjxJ/k60XP).

*CYP97C1* (or *LUT1*) is involved in the production of the carotenoid lutein, a dihydroxy derivative of α-carotene, and maybe β-carotene [[156, 157]](https://paperpile.com/c/slGjxJ/hEzUN+Gn6VK). It is the most abundant carotenoid in the photosynthetic plant tissues and precursor in the synthesis of abscisic acid (ABA) [[158]](https://paperpile.com/c/slGjxJ/KzJrQ). Overexpression of *CYP97C1* led to an increase in zeinoxanthin and zeaxanthin, as well as a decrease in α-carotene and β-cryptoxanthin [[157]](https://paperpile.com/c/slGjxJ/Gn6VK). Carotenoids are in constant turnover, and oxidative cleavage catalyzed by carotenoid cleavage dioxygenases (CCDs) leads to the production of terpenoid compounds called apocarotenoids [[159–161]](https://paperpile.com/c/slGjxJ/DTotT+AzY4Z+pQFGg). Apocarotenoids have many important biological functions in plants, such as precursors of the phytohormones abscisic acid (ABA; stress/defense hormone) and strigolactones (SL; growth hormone), as well as apocarotenoid volatile organic compounds (VOCs) such as β-ionone [[160]](https://paperpile.com/c/slGjxJ/AzY4Z). β-ionone emissions increased in response to *Pieris rapae* caterpillar feeding vibrations alone [[162]](https://paperpile.com/c/slGjxJ/kayRt). β-ionone from clovers *Trifolium glanduliferum* and *Trifolium strictum* was a strong feeding deterrent of the redlegged earth mite *Halotydeus destructor* [[163]](https://paperpile.com/c/slGjxJ/YStwk). A similar result was reported for the two spotted spider mite *Tetranychus urticae* [[164]](https://paperpile.com/c/slGjxJ/wLxkA). *Brassica* β-ionone also had a repellent and feeding deterrent effect against the crucifer flea beetles *Phyllotreta cruciferae* [[165, 166]](https://paperpile.com/c/slGjxJ/88lvz+eGnr2). Flea beetle movements were strongly inhibited by the terpene VOCs linalool, limonene, β-pinene, geraniol, caryophyllene, and β-ionone [[165]](https://paperpile.com/c/slGjxJ/88lvz).​​Additionally, linalool and β-pinene were toxic to the confused flour beetle *Tribolium confusum* [[167]](https://paperpile.com/c/slGjxJ/Jmlpf). Lastly, β-ionone was a strong oviposition deterrent to silverleaf whiteflies *Bemisia tabaci* [[164]](https://paperpile.com/c/slGjxJ/wLxkA).

*PBL36* is a receptor-like cytoplasmic kinase involved in *CLE* and *BAM* signaling to maintain root meristem homeostasis. In particular, it is required for *CLAVATA 3* (*CLV3*) mediated-signaling in the shoot apical meristem, as well as *CLE25/26/45*-mediated signaling in the root apical meristem [[168]](https://paperpile.com/c/slGjxJ/ps4eH). Phosphorylation of the cytoplasmic receptor kinase *PBL36* leads to the activation of a MAPK signaling pathway, and therefore, activates plant immunity [[169]](https://paperpile.com/c/slGjxJ/Yv1LM). *Pseudomonas syringae* bacterial effector HopAO1 reduces phosphorylation of *PBL36*, suppresses immune response, and triggers host susceptibility in *A. thaliana* [[169]](https://paperpile.com/c/slGjxJ/Yv1LM).

*OGOX1*(or *BBE19*), an oligogalacturonide (OG) oxidase, is involved in plant immunity. OGs, a major component of pectin, are a well-known class of damage-associated molecular patterns (DAMPs) that activate immunity and protect plants against microbes [[170]](https://paperpile.com/c/slGjxJ/SwLBh). *A. thaliana* plants overexpressing *OGOX1* are more resistant to the fungus *Botrytis cinerea*, likely due to the difficult digestion of the oxidized OG dimer by fungal polygalacturonases [[171]](https://paperpile.com/c/slGjxJ/zp38e).

The defense-related gene *OSM34* was upregulated in flowering *A. thaliana* plants by *Pieris brassicae* eggs and feeding [[172]](https://paperpile.com/c/slGjxJ/4aAC9). *OSM34* was downregulated in giant cells during compatible interaction between *A. thaliana* and root-knot nematodes *Meloidogyne incognita* and *M. javanica* [[173]](https://paperpile.com/c/slGjxJ/6WzEM). The expression of *OSM34* was suppressed by the virulent soybean cyst nematode *Heterodera glycines* population in soybean *Glycine max* [[174]](https://paperpile.com/c/slGjxJ/QBmAU).

*IBR10* is a peroxisomal β-oxidation gene encoding for an IBA-specific enoyl-CoA hydratase involved in IBA to IAA conversion [[175, 176]](https://paperpile.com/c/slGjxJ/Rms8t+hmlXr). Auxin IAA regulates growth *via* cell elongation in stems, and plays a role in cell division/differentiation [[158]](https://paperpile.com/c/slGjxJ/KzJrQ). Additionally, IAA increased rapidly during feeding by the *Manduca sexta* caterpillars, or by treatment with the insect elicitor *N*-linolenoyl-Glu, as well as by mechanical wounding to a lesser extent, leading to the production of defenses in tobacco *Nicotiana attenuata* [[177, 178]](https://paperpile.com/c/slGjxJ/ziB4O+fRW5A).

*EXT3* is associated with normal cell wall formation, as well as remodeling [[179]](https://paperpile.com/c/slGjxJ/kUaXg). In *A. thaliana*, the expression of *EXT3* was elevated specifically at the wounding site [[180]](https://paperpile.com/c/slGjxJ/3BOrC), as well as in response to *Plutella xylostella* caterpillar feeding [[181]](https://paperpile.com/c/slGjxJ/H5Y6I).

AT5G11610 is an exostosin family protein. This gene was induced during resistance of *A. thaliana* against the fungus *Botrytis cinerea* [[182]](https://paperpile.com/c/slGjxJ/LlnMs).

***Downregulated DEGs*** *—* Among the downregulated genes, we identified *LEAF WILTING 1* (*LEW1*, Log2FC = -1.69) and *GAST1 PROTEIN HOMOLOG 4* (*GASA4*, Log2FC = -1.96).

*LEW1* encodes a heteromeric *cis*-prenyltransferase involved in the synthesis of dolichol, which is required for the biosynthesis of biologically important N-linked glycoproteins [[183]](https://paperpile.com/c/slGjxJ/6NdcR). Dolichol itself is synthesized by a common terpenoid pathway from acetate [[183]](https://paperpile.com/c/slGjxJ/6NdcR). Dolichols promote protein trafficking in the endoplasmic reticulum by carrying sugars for protein glycosylation [[184]](https://paperpile.com/c/slGjxJ/I0lXN). A mutation in *lew1* can cause a loss of membrane integrity and reduced protein glycosylation [[184]](https://paperpile.com/c/slGjxJ/I0lXN).

The gibberellin-induced *GASA4* is involved in cell division in *A. thaliana* [[185]](https://paperpile.com/c/slGjxJ/WNH31). *GASA4* is upregulated in all meristematic regions in *A. thaliana*, including vegetative, inflorescence, and floral meristems [[185]](https://paperpile.com/c/slGjxJ/WNH31).

***2.6 Day 6***

On day 6, we found 27 differentially regulated *Arabidopsis* orthologs, all 20 genes (27 loci) were upregulated.

***Upregulated DEGs*** *—* Among the upregulated genes, we identified *RLP1* (Log2FC = 2.96-5.19, 2 loci), *ECH* (Log2FC = 4.44), *ASG5* (or *PBL21*, Log2FC = 2.63-2.89, 2 loci), *PBL36* (Log2FC = 2.96), *UGT73B4* (Log2FC = 6.09), *NAC103* (Log2FC = 3.12), NAC082 (or *VNI1*, Log2FC = 2.84), *MIK2* (Log2FC = 1.72-4.36, 2 loci), *OSM34* (Log2FC = 3.78), *RECEPTOR LIKE PROTEIN 54* (*RLP54, Log2FC = 2.03*), *CYTOCHROME P450, FAMILY 82, SUBFAMILY G, POLYPEPTIDE 1* (*CYP82G1*, Log2FC = 1.95), *LACCASE 14* (*LAC14*, Log2FC = 2.29), as well as AT4G38660 (Log2FC = 5.28), a pathogenesis-related thaumatin superfamily proteins involved in plant defense.

*RLP54* is a pattern recognition receptor (PRR), part of a disease resistance family protein [[81]](https://paperpile.com/c/slGjxJ/Lf9Fu). *RLP54* was upregulated during resistance upon plant inoculation with infective second-stage nematode juveniles of *Heterodera schachtii* and treatment with NemaWater, a cocktail of nematode elicitors, in *A. thaliana* [[81]](https://paperpile.com/c/slGjxJ/Lf9Fu). Homoterpene plant volatiles released from herbivore-damaged tissue contribute to indirect plant defense by attracting natural enemies of the attacker, and induce defensive responses.

*CYP82G1* is responsible for the breakdown of the C20-precursor (E,E)-geranyllinalool into the insect-induced C16-homoterpene (E,E)-4,8,12-trimethyltrideca-1,3,7,11-tetraene (TMTT) in *A. thaliana* [[186]](https://paperpile.com/c/slGjxJ/XpPT7). TMTT is a repulsive volatile for aphids *Aphis gossypii* and *Aphidius ervi* [[187]](https://paperpile.com/c/slGjxJ/f7bTh). TMTT production was induced upon *Pieris rapae* or *Plutella xylostella* caterpillar infestation in *A. thaliana* [[188]](https://paperpile.com/c/slGjxJ/puWpM). The volatile blend, including TMTT, emitted by *P. rapae*-infected *A. thaliana* plants was attractive to the parasitic wasp *Cotesia rubecula*, and parasitism enhanced the plant fitness [[188, 189]](https://paperpile.com/c/slGjxJ/puWpM+MgpjZ). TMTT concentration was also elevated during infestation by the spider mite *Tetranychus urticae* in lima bean *Phaseolus lunatus* which induced the expression of *PR2*, *PR3*, *LOX*, *PAL*, and *FPS* genes [[190]](https://paperpile.com/c/slGjxJ/FcbTt). In the same system, the predatory mite *Phytoseiulus persimilis* was specifically able to locate *T. urticae*-infested lima bean plants using the herbivore-induced volatile blend, which included TMTT and methyl salicylate (MeSA) [[191]](https://paperpile.com/c/slGjxJ/4Amkl). However, *T. urticae*-infested lima bean plants were less attractive to the predatory mite when the plants were also treated with a terpenoid pathway inhibitor (fosmidomycin), which severely reduced the emission of homoterpenes [[192]](https://paperpile.com/c/slGjxJ/wBTU4). Finally, TMTT is involved in plant-plant communication by triggering the systemic induction of a LOX gene, activation of JA signaling pathway, and expression of defense genes in healthy lima beans, priming them for a faster defense response upon a subsequent attack [[190]](https://paperpile.com/c/slGjxJ/FcbTt).

*LAC14* is involved in lignin deposition [[193]](https://paperpile.com/c/slGjxJ/pa81L). Overexpressioon of *LAC14* in poplar *Populus tomentosa* promoted the lignification of poplar and reduced the proportion of syringyl/guaiacyl, while the knockout of this gene led to an increase of the syringyl/guaiacyl ratios [[193]](https://paperpile.com/c/slGjxJ/pa81L). The expression of *LAC14* was elevated against the root-knot nematode *Meloidogyne incognita* in cotton *Gossypium hirsutum* resistant genotype only [[194]](https://paperpile.com/c/slGjxJ/obt9A).

***2.7 Day 7***

On day 7, we found 1 differentially regulated *Arabidopsis* ortholog. We identified the upregulated gene *RLP1* (Log2FC = 2.98).

***3. Overall changes induced by oviposition by the gall-inducing wasp***

Early on, the number of DEGs was much greater in the resistant genotype than in the susceptible genotype (Figure 4A). In the resistant genotype, we can describe two waves of plant response: (1) strong response to oviposition (wounding/eggs + immunity priming) on day 1, and (2) strong resistance response starting on day 4 until day 6, with a minimal number of DEGs in between. In the susceptible genotype, we can describe three waves: (1) (reduced but present) immune response to oviposition on day 1 (wounding/eggs), (2) slight change when the cells surrounding the eggs become meristematic on day 4, and (3) elevated response during gall initiation starting on day 6 and continuing. The hypersensitive response in the resistant genotype, leading to the insect death, occurs within the first few days post-oviposition, while the initiation of gall development in the susceptible genotype starts on 3-4 dpo.

Our coexpression networks allowed us to characterize the changes occurring at the oviposition site on both genotypes (see Figure 6 and Supplemental Table S5 for the details of GO categories). In the resistant genotype, based on the results presented in section 4, we identified an upregulation of DEGs involved in (*i*) axis/polarity, cell wall, defense response, stress/defense phytohormone, immunity, meristem, phytohormone, plant organ, priming, reproduction, resistance, response to biotic stress, stomata, tissue development, and wounding on day 1, (*ii*) cell wall, defense response, growth phytohormone, pigments, response to stress, reproduction, resistance, response to biotic stress, stomata, and wounding on 1 and 5 dpo, as well as (*iii*) axis/polarity, cell cycle, cell wall, chromatin, defense response, stress/defense phytohormone, primary metabolism, and response to biotic stress on 5-6 dpo. In contrast, in the susceptible genotype, we determined that upregulated DEGs were involved in cell wall, defense response, growth phytohormone, pigments, response to stress, reproduction, resistance, response to biotic stress, stomata, and wounding, on 1 dpo only. We also found downregulated DEGs involved in axis/polarity, cell cycle, cell wall, chromatin, defense response, stress/defense phytohormone, primary metabolism, and response to biotic stress, axis/polarity, cell cycle, cell wall, defense response, stress/defense phytohormone, growth hormone, phytohormone, plant organ, and resistance on 6-7 dpo.

***Wounding response***

We only identified a few DEGs involved in the wounding response. This is due to the fact that our controls on both genotypes were mechanically damaged with a needle to mimic the stabbing behavior exhibited by the female wasp after laying its eggs into the stem. The wounding response is therefore minimal in this experiment, allowing for the characterization of the plant’s response to the insect only.

***Receptor-mediated recognition of oviposition***

Plant immunity can be activated by the specific perception and recognition of DAMPs, MAMPs, PAMPs, EAMPs (​​egg-associated molecular patterns), or HAMPs (​​herbivore-associated molecular patterns) by plant pattern recognition receptors (PRRs) or through identification by a resistance (R) protein-mediated process against race-specific effector molecules of pathogens[[195–198]](https://paperpile.com/c/slGjxJ/XkDmT+pkM71+6Lmnh+j9SYt). Several DEGs encoding for receptors were upregulated upon oviposition, with a larger number on the resistant genotype than in the susceptible genotype (Figure 5).

In the resistant genotype, we identified two receptor-like proteins (RLP) that are leucine-rich repeat receptor protein (LRR-RP)-type pattern recognition receptors (PRRs): *RLP1* (1 and 5-7 dpo, 4 loci on 5 dpo and 2 loci on 6 dpo) and *RLP54* (6 dpo). Not a DEG, but *RLP7* was also upregulated in this genotype (1-2, 4, and 7 dpo, but downregulated on 6 dpo). We also found two upregulated receptor-like kinases that are leucine-rich repeat receptor kinases (LRR-RK) and function as a PRR: *MIK2* (1 and 5-6 dpo, 8 loci on 5 dpo and 2 loci on 6 dpo) and *RLK7* (5 dpo). Additionally, we identified one L-type lectin receptor kinase and one disease-resistance locus receptor that were upregulated: *LECRK-S.5* (1 and 4 dpo) and *LRK10L1.2* (5 dpo), respectively. Lastly, we found two receptor-like cytoplasmic kinases (RLCK) that were upregulated in the resistant genotype: *PBL21* (1 and 4-6 dpo, 2 loci on 4 and 6 dpo) and *PBL36* (5-6 dpo).

In contrast, in the susceptible genotype, we found one upregulated RLP: *RLP1* (1, 3, 7 dpo), and not a DEG, but *RLP7* was also upregulated on 1 dpo. We also identified the LRR-RK *MIK2* (6 dpo) and RLCK *PBL36* (1 dpo) as upregulated DEGs in the susceptible genotype. We found a L-type lectin receptor kinase, different from the one in the resistant genotype, that was downregulated (*LECRK-VIII.2*, 6 dpo).

Additionally, the GWA analysis returned the QTL AT1G66880, encoding for a RLK with a LRKL extracellular domain and belonging to the LRK10L-1 subfamily, as a potential marker of blueberry resistance to the wasp. This receptor was activated in response to *P. brassicae* oviposition, nematode effectors, bacterial elicitors, and Turnip mosaic virus[[115, 199–202]](https://paperpile.com/c/slGjxJ/dkhu1+UM6V7+jlUJE+m1Oou+dxAiy).

All of these receptors could participate in the detection of the eggs oviposited within the stem tissues and/or oviposition fluids injected into the plants tissues along with the eggs, and could trigger the appropriate defense cascade to prevent further infestation by the wasp. Additional experiments will be necessary to determine whether and which receptors are required to mount the immune response in the blueberry highbush *V. corymbosum*.

***Pattern-Triggered Immunity (PTI) and Effector-Triggered Immunity (ETI)***

We found components belonging to PTI and ETI in the resistant genotype’s DEGs as soon as 1 dpo, while they were lacking or limited in the susceptible genotype (Figure 5). PTI is induced when pattern recognition receptors (PRRs, extracellular immune receptors) perceive generic molecules from microorganisms, and confers moderate disease resistance to a broad spectrum of pathogens[[60, 203]](https://paperpile.com/c/slGjxJ/vayWU+iB7so). ETI is activated upon recognition of the microorganism’s secreted effectors by intracellular immune receptors, responsible for strong and localized resistance to a specific pathogen carrying an avirulence (*Avr*) gene, and indispensable for establishing basal, specific immunity to virulent pathogen infection[[204, 205]](https://paperpile.com/c/slGjxJ/xoGO3+r7wdR). These two branches of plant immunity establish a robust defense response, and they synergistically enhance each other. ETI increases the abundance of signaling components, potentiating the PTI pathway, and thus amplifying the hypersensitive response[[203, 204]](https://paperpile.com/c/slGjxJ/xoGO3+vayWU).

The components of PTI and ETI identified in our resistant blueberry plants included, but not limited to, PRR induction (*RLP1* on days 1 and 5-7; *RLP54* on day 6; *RLK7* on day 5; *MIK2* on days 1 and 5-6), ROS production with H_2_O_2_ accumulation (*EDS1* on days 1), callose deposition in the plasmodesmata (*PBL1* on day 3) and inhibition of callose degradation (*BG_PPAP* on day 1), hypersensitive response leading to cell death (*MTK1* on day 1; *NAC103* on days 1, 4, and 6) at the oviposition site, and release of volatile organic compounds (VOCs) to attract natural enemies (*WRKY40* on day 1). We also found two PRRs (*RLP1* on days 1, 3, and 7; *MIK2* on day 6) and genes involved in H_2_O_2_ accumulation (*EDS1* on day 1) that were DEGs in our susceptible blueberry plants. However, in the susceptible genotype, the activation of these genes did not result in the hypersensitive response observed in the resistant genotype. While both genotypes exhibited some components of PTI and ETI resistance, only the resistant genotype mounted a wide, strong immune response leading to the hypersensitive response occurring at the oviposition site.

***Localized Hypersensitive Response (HR) and Systemic Acquired Resistance (SAR)***

Pathogenesis-related proteins (PRs) are induced upon plant attack by viruses, bacteria, fungi, nematodes, and insects leading to hypersensitive necrotic response (HR)[[206–208]](https://paperpile.com/c/slGjxJ/oFw6W+s1SYB+uFZgd). While induction of phytoalexins and cell wall rigidification are local reactions, PR accumulation is systemic upon infection and leads to SAR. In our system, only the resistant blueberry genotype mounted a SAR response upon *H. nubilipennis* oviposition.

We identified two uncharacterized PR thaumatin superfamily proteins among our DEGs. AT1G20030 was downregulated on 6 dpo in the susceptible genotype, while in the resistant genotype, it was upregulated on 1 and 4 dpo. Additionally, AT4G38660, belonging to the PR5 family, was upregulated in the resistant genotype only on 1-2 and 4-6 dpo.

***Plant Cell Wall-Mediated Resistance and Susceptibility to the Wasp***

Plant cell walls are the first physical and defensive barrier against pathogens. Cell walls usually undergo dynamic remodeling as an immune response upon infection. Components and modification of cell walls are associated with PTI and ETI[[209, 210]](https://paperpile.com/c/slGjxJ/zDWoB+d7AlG) (Figures 4C and 5). It is therefore not surprising that pathogens secrete effectors to target and dampen plant cell wall integrity and defense response.

In the resistant genotype, *LAC14*, a gene involved in lignin deposition, leading to a strengthening of cell walls, was upregulated on day 6[[211]](https://paperpile.com/c/slGjxJ/BSi3K). As a complex phenolic polymer, lignin enhances plant cell wall rigidity, and is an important barrier that protects against pests and pathogens such as root-knot nematode *Meloidogyne incognita* in *A. thaliana* and *​​Nicotiana tabacum* tobacco plants[[212]](https://paperpile.com/c/slGjxJ/XoCjy). Along with cell wall fortification, we also observed that the uncharacterized gene AT4G13210, encoding for a pectate lyase, was downregulated during all seven days post-oviposition, leading to an inhibition of cell wall degradation. Cell wall breakdown was observed at the feeding site in susceptible wheat plants attacked by Hessian flies[[213]](https://paperpile.com/c/slGjxJ/pb9Lk), while in contrast, cell wall fortification was noticed in resistant plants[[214]](https://paperpile.com/c/slGjxJ/5QBja). In our system, the maintenance and/or reinforcement of cell wall integrity/thickening at the oviposition site could thus participate in blueberry resistance by confining the wasp eggs during the resistance response.

Additionally, the upregulation of *GLUCAN SYNTHASE-LIKE 12* (*GSL12*; 1-2 and 4-5 dpo), and downregulation of *BG_PPAP* (1 dpo), a β-1,3-glucanase, should affect plasmodesmata (PD) permeability and symplastic cell-to-cell communication by limiting callose degradation[[129, 215]](https://paperpile.com/c/slGjxJ/cbppl+o1XgH). Another cue pointing out callose accumulation along PD is the strong upregulation of calreticulin genes at the oviposition site. These genes include *CALRETICULIN 1A* (*CRT1A*; 1-7 dpo), *CALRETICULIN 1B* (*CRT1B*; 1, 3, and 6-7 dpo), *CALRETICULIN 3* (*CRT3*; 3-7 dpo), and the uncharacterized gene AT5G07340 (1, 3, and 6-7 dpo) encoding for calreticulin family protein. Calreticulin preferentially localizes to PD, and accumulates at callose-enriched PDs[[216]](https://paperpile.com/c/slGjxJ/6ieP3). Callose deposition along PD is a mechanism used by plants to impede the spread of invading pathogens during resistance response[[217–220]](https://paperpile.com/c/slGjxJ/GDUOs+RFbRJ+PIIvh+G9Gl1). In *Oryza sativa* rice, attack by the brown planthopper *Nilaparvata lugens* leads to the activation of callose deposition, a key resistance mechanism[[221]](https://paperpile.com/c/slGjxJ/t5cAk). Callose deposition in galls also negatively affected rice parasitism by the root-knot nematode *Meloidogyne graminicola* by decreasing PD permeability, and thus reducing PD-mediated sucrose supply[[222]](https://paperpile.com/c/slGjxJ/zwuiQ). In our resistant genotype, alterations of PD permeability could be linked with blueberry resistance by (*i*) limiting cell-to-cell communication necessary to initiate the gall, and/or (*ii*) preventing the establishment of a nutrient sink at the site of oviposition.

The upregulation of AT1G67750 (6 dpo, pectate lyase involved in pectin catabolism), and downregulation of *FRA8* (6 dpo, xylan glucuronyltransferase), *GXM1* (4 dpo, glucuronoxylan methyltransferase), *COB* (7 dpo), *TBL33* and *TBL34* (7 dpo and 6-7 dpo respectively, xylan acetyltransferase[[223]](https://paperpile.com/c/slGjxJ/8qFjU)), *CESA4* and *CESA7* (6-7 dpo and 7 dpo respectively, cellulose synthase), *CAD4* and *CAD5* (6-7 dpo), should lead to a decrease of the cellulose, lignin (monolignol), pectin, and xylan (hemicellulose) content in the cell walls, as well as xylan acetyl content. A pectate lyase (AT4G13210) was also upregulated on days 2-3 and 5-6 dpo (downregulated on days 1 and 4). This enzyme is involved in pectin cleavage during cell wall degradation. Moreover, *ARA12*, a subtilisin-like protease inhibiting cell wall weakening, was downregulated on day 5. In the susceptible genotype, the observed changes in cell-wall related gene expression should loosen and/or weaken the cell walls at the oviposition site, which could therefore increase the susceptibility to the pathogen[[224]](https://paperpile.com/c/slGjxJ/KYVVQ), and contribute to gall initiation.

***Similarities between fruit development and nutritive tissue formation in galls***

Many galls resemble flowers or fruits (see Figure 1 from[[225]](https://paperpile.com/c/slGjxJ/ZmAbo)). In the system phylloxera *Daktulosphaira vitifoliae* / wild grape *Vitis riperia*, Schultz et al. showed that phylloxera leaf galls are developmentally and transcriptionally convergent on floral organs, particularly the carpel. If, in our system, the wasp also activates plant reproductive pathways to induce its gall, then it is not surprising that we identified genes from nine GO categories related to plant reproduction in our most significant coexpression network modules (Figures 6).

Expansins are proteins that play a critical role in cell wall modification and growth during plant development, such as fruit ripening. Expansins are also involved in the establishment of nematode syncytium[[226]](https://paperpile.com/c/slGjxJ/oLyZB), as both fruit ripening and syncytium formation require cell wall modifying proteins, such as polygalacturonases, pectate lyases, pectin methylesterase, endoglucanases, xyloglucan endotransglycosylase and expansins[[227–229]](https://paperpile.com/c/slGjxJ/c97mZ+sxrWc+oT6Ym). In tomato roots infected with the potato cyst nematode (*Globodera rostochiensis*), the increase in expansin expression was linked to increased susceptibility and formation of syncytial feeding structures[[25, 230]](https://paperpile.com/c/slGjxJ/xdnaV+UWfym). In our susceptible genotype, the upregulation of two expansins, *EXPA1* and *EXPA5* (6 dpo), suggests the loosening of cell wall hemicellulose-cellulose interactions, facilitating the enlargement of plant cells during gall initiation and development[[25, 230, 231]](https://paperpile.com/c/slGjxJ/xdnaV+UWfym+h7Tql).

***Stress/defense and growth phytohormone biosynthesis***

Among our DEGs, we identified genes involved in stress/defense phytohormone biosynthesis, more specifically SA and ET (Figures 4C and 5). In our susceptible genotype, *UGT74F1* expression patterns suggest an increased SA metabolism on day 1 that should lead to resistance, and a decrease on days 6-7, when the larvae have hatched and start feeding. In contrast, in the resistant genotype, the expression pattern suggests an enhanced resistance lasting longer (days 1-3) than in the susceptible genotype. Additionally, in the susceptible genotype, *SAM2* expression pattern suggests a small initial production of ET, but no following peak, suggesting an impediment of ET production. In the resistant genotype, its slight upregulation on day 1 (initial peak) was followed by upregulation on day 6 (following peak) should enhance ET emission. Such an increase of ET production on day 6 participates in the resistance response to wasp oviposition *via* cell death (HR; Figure 1B).

The enzyme *UGT74F1* is involved in the conversion of SA into its glucoside (SAG), and can inhibit the jasmonic acid (JA) pathway[[232]](https://paperpile.com/c/slGjxJ/xs2fD). *A. thaliana* plants failing to accumulate SA and SAG become more susceptible to bacterial (*Pseudomonas syringae*) and fungal (*Golovinomyces orontii*) infection[[232, 233]](https://paperpile.com/c/slGjxJ/AP19t+xs2fD). In our system, this gene was upregulated on day 1 in the susceptible genotype, and should lead to an enhanced conversion of SA into SAG. However, its downregulation on days 6-7 could increase the susceptibility of the host-plant to the wasp. In contrast, *UGT74F1* was upregulated on days 1-3, suggesting an enhanced resistance lasting longer than in the susceptible genotype.

The enzyme *SAM2* catalyzes the production of S-adenosyl-methionine (SAM), SAM is then converted into ethylene’s precursor 1-aminocyclopropane-1-carboxylate (ACC)[[46]](https://paperpile.com/c/slGjxJ/mXnC4). Ethylene (ET) is a volatile organic compound (VOC) involved in many plant developmental and physiological processes, and can be induced upon wounding and biotic stress[[234]](https://paperpile.com/c/slGjxJ/dSDgP). ET is known to intervene in the development of resistance against biotic stressors by inducing the expression of defense-related genes, such as PR proteins and phytoalexins[[235, 236]](https://paperpile.com/c/slGjxJ/ypnJM+6koMt). Wounding-induced ET production is called stress-ET, which mediates wounding signaling, leading to the induction of defense-related genes. When a biotic stress occurs in plants, there is a small first peak in ET production, followed by a larger second peak. The initial production uses ACC available within the plant tissues, while the following production of ET causes the expression of ACC synthase genes[[237, 238]](https://paperpile.com/c/slGjxJ/jH0Ys+ZZOOm). The late production of ET causes chlorosis, senescence, and abscission. ET is a modulator of stress-induced cell death and apoptosis[[239]](https://paperpile.com/c/slGjxJ/41aW0). In the susceptible genotype, *SAM2* was slightly upregulated on day 1 (initial peak), but downregulated on days 2-3 and 5-7, which should impede ET production (no following peak). In the resistant genotype, its slight upregulation on day 1 (initial peak) was followed by upregulation on day 6 (following peak) should enhance ET emission.

We also identified DEGs involved in growth phytohormone biosynthesis (Figures 4C and 5). These included *IBR10* involved in auxin (AUX) biosynthesis. The enzyme IBR10 is involved in the conversion of AUX indole-3-butyric acid (IBA) into AUX indole-3-acetic acid (IAA). AUX regulates many aspects of plant growth and development by directing cell division, cell elongation, and cell differentiation, wound healing, stem bending, and leaf epinasty[[240–244]](https://paperpile.com/c/slGjxJ/AMj8F+l6PQB+AUk8D+UIGmn+fqLq7). The conversion of IBA into IAA suggests that IBA acts as a storage sink for IAA[[245]](https://paperpile.com/c/slGjxJ/OtRj7). However, IBA can also work as an active AUX on its own[[246]](https://paperpile.com/c/slGjxJ/MGRPh). IAA increases during the late stage of wound healing[[244]](https://paperpile.com/c/slGjxJ/fqLq7). The conversion of IBA into IAA was upregulated on days 1 and 5 in the susceptible genotype, while it was upregulated on days 1-7 in the resistant genotype. The upregulation of *IBR10* should lead to an accumulation of IAA at the oviposition site on both genotypes. However, the role of such accumulation could have similar or different roles in both genotypes depending on the concentration and timing; for example: cell division/growth during compatible interaction (gall initiation) in the susceptible genotype and wound healing (HR) during incompatible interaction in the resistant genotype. Only further investigation would clarify the role of AUX at the oviposition site in both genotypes.

Additionally, our GWA analysis returned three QTL genes involved in growth phytohormone biosynthesis (Supplemental Table S1). These included *CYP735A1* and *STE1*, involved in cytokinin (CK) and brassinosteroid (BR) biosynthesis, respectively.

The CYP735A1 enzyme catalyzes the conversion of CK isopentenyladenine (iP) precursors into CK *trans*-zeatin (*t*Z) precursors[[247]](https://paperpile.com/c/slGjxJ/qq7Co). CKs affect many aspects of plant growth and development, such as cell division, wound healing sink/source relationships, nutrient uptake, as well as vascular and embryonic development, often acting in concert with other phytohormones, most notably AUX[[244, 248]](https://paperpile.com/c/slGjxJ/vcEQ7+fqLq7). CKs can be increased by wounding, leading to the stimulation of cell division and tissue repair[[249, 250]](https://paperpile.com/c/slGjxJ/XFTaP+oFfUZ). Studies also reported a role of CKs in enhancing plant resistance against plant pathogens, such as pest insects (gypsy moth *Lymantria dispar*[*[251]*](https://paperpile.com/c/slGjxJ/i66NJ), specialist herbivore *Manduca sexta*[*[252]*](https://paperpile.com/c/slGjxJ/fBFmG)), fungi (*Erysiphe graminis*[*[253]*](https://paperpile.com/c/slGjxJ/6M1n5), *Magnaporthe oryzae*[*[254, 255]*](https://paperpile.com/c/slGjxJ/3iMF0+68Rj1)), and bacteria (*Pseudomonas syringae*[*[256–258]*](https://paperpile.com/c/slGjxJ/VxxKQ+UUR3e+AfvCV)). Interestingly, the bacterial phytopathogen *Rhodococcus fascians* secretes a mix of CKs, including *t*Z and 2-methylthio-*cis*-zeatin to induce the production of leafy galls on *A. thaliana*[*[259]*](https://paperpile.com/c/slGjxJ/NNhnu). In the susceptible genotype, this gene was upregulated on days 3-4 when the cells surrounding become meristematic, as well as on day 7 after gall initiation started, suggesting a role of *t*Z in cell division and growth at the oviposition site on blueberry. In contrast, in the resistant genotype, the downregulation of this gene on days 1 and 4-5 should lead to an accumulation of iP, while its upregulation on days 6-7 dpo suggests a conversion of the accumulated iP into *t*Z. While we can establish the difference expected in the composition of the CK pool, we cannot assess the impact on blueberry without bioassays. A slight bump appears during HR on the stem where the oviposition wounds are located (Figure 1B); therefore, CKs could be involved in cell division, wound healing, and/or resistance in the resistant genotype.

The STE1 enzyme catalyzes a step in the biosynthesis of campesterol, the starting point for BR biosynthesis[[260]](https://paperpile.com/c/slGjxJ/OX3va). BRs are a group of steroid phytohormones structurally similar to animal and insect steroids, and regulate many processes in plant growth and development[[261]](https://paperpile.com/c/slGjxJ/5L10C). These functions include cell development, cell elongation, cell division, flowering, and biotic stress response, among others[[261–264]](https://paperpile.com/c/slGjxJ/tGIms+D5BaQ+bobXc+5L10C). *STE1* expression suggests the production of BR only on day 1 in the susceptible genotype, and only on day 5 in the resistant genotype.

Future investigation will include examination of the expression of all the genes involved in phytohormone biosynthetic pathway to establish the timeline of induced phytohormone production in both genotypes after exposition to wasp oviposition. Additionally, phytohormone profiling will allow the comparison of gene expression and phytohormone signature, and would highlight potential post-transcriptional regulation of phytohormone biosynthesis.

***Direct and indirect defense***

Upon biotic attack, plants activate defense cascades, including but not limited to (*i*) direct chemical defenses to harm and/or deter the attacker, and (*ii*) indirect chemical defenses with the emission of volatile organic compounds (VOCs) to attract natural enemies of the attacked and communicate with intact neighboring plants[[265, 266]](https://paperpile.com/c/slGjxJ/xOGJy+HALUs). In our susceptible genotype, the DEG *ILR3*, encoding for aliphatic glucosinolates was downregulated on 5-7 dpo, while *NAC082*/*VNI1* involved in flavonoid biosynthesis was upregulated on 1 dpo. In contrast to the limited defense response in the susceptible genotype, the resistant genotype’s DEGs showed an upregulation of phenylpropanoid, terpenoid, and lipoxygenase pathways. In the phenylpropanoid pathway, these included *ECH* (4-6 dpo) required for flavonoid accumulation, *TT7* (1 dpo) encoding for the flavonoid 3’ hydrolyse involved in the production of flavonols, *WRKY18* (1 dpo) encoding for isoflavonoid phytoalexins, as well as *NAC082*/*VNI1* which was upregulated on day 1 similarly to the susceptible genotype, but also on 4-6 dpo in the resistant genotype. DEGs from the terpenoid pathways should enhance the emission of VOCs. *CYP97C1* (5 dpo) and *CYP82G1* (6 dpo), encoding for the biosynthesis of the apocarotenoid β-ionone and homoterpene TMTT respectively, were upregulated and should lead to an increase of this diterpenoid VOC bouquet. Lastly, the DEG *WRKY40*, encoding the biosynthesis of the green leaf volatile E-2-hexenal *via* the (13-hydroperoxide) lipoxygenase pathway, was upregulated on day 1 dpo. Moreover, our GWA analysis returned the QTL *SCL14* involved in cJ production *via* the same pathway as a potential candidate for blueberry resistance. The upregulation of this gene on 1 and 4 dpo (but slightly downregulated on 2 dpo) in the resistant genotype should enhance direct and indirect plant defense against insect herbivores[[267]](https://paperpile.com/c/slGjxJ/PKzYD) by repelling harmful herbivorous insects and attracting beneficial natural enemies of the attacker[[268]](https://paperpile.com/c/slGjxJ/Wpuoo).

While the defense-related DEG was only upregulated on 1 dpo in the susceptible genotype, we found multiple DEGs upregulated on days 1 and 4-6 dpo in the resistant genotype. These observations suggest the production of plant defense in both genotypes on day 1, but to a lesser extent in the susceptible genotype than in the resistant genotype (Figures 4C and 5). Additionally, only the resistant genotype had upregulated DEGs involved in secondary metabolite biosynthesis. These results indicate that the resistant genotype mounts a stronger and long-lasting defense response upon the wasp oviposition, while it seems like the susceptible genotype tolerates the presence of the insect past the wounding response occurring on 1 dpo. The production of such a defense blend is most likely one of the keys of blueberry resistance to the gall-inducing wasp.

***Role of oviposition fluids in plant immune response and gall initiation success***

Galls start to become visible on the susceptible genotype on 3-4 dpo (Figure 1A). However, larvae only hatch on 12-14 dpo[[269, 270]](https://paperpile.com/c/slGjxJ/JnY9q+8cgd7). This observation suggests that the mechanisms underlying gall initiation start when the female wasp oviposits her eggs, rather than when the eggs hatch. Female parasitoid wasps are known to inject venom into the host along with the eggs[[271]](https://paperpile.com/c/slGjxJ/K9Tgp), and a recent study in galling wasps of oak trees and rose bushes found highly expressed genes in the venom gland and ovaries that are thought to be involved in early plant defense suppression[[272]](https://paperpile.com/c/slGjxJ/jsheO). Taken together, this suggests a role for secretions from the female’s venom glands and/or ovaries in gall formation in blueberry. Further analyses will be required to demonstrate that a similar mechanism is employed by *H. nubilipennis* to evade the host-plant’s immune system and initiate the gall formation.

In conclusion, we identified candidate genes that could explain the resistance to the wasp *H. nubilipennis* wasps. In response to wasp oviposition, the resistant genotype mounted a stronger and longer lasting resistance response than the susceptible genotype. Additionally, this oviposition-induced resistance in the resistant genotype included local and systemic responses.

**Bibliography**

[1. Falk A, Feys BJ, Frost LN, Jones JD, Daniels MJ, Parker JE (1999) *EDS1*, an essential component of *R* gene-mediated disease resistance in *Arabidopsis* has homology to eukaryotic lipases. *Proceedings of the National Academy of Sciences of the United States of America*, 96(6):3292–3297. https://doi.org/](http://paperpile.com/b/slGjxJ/RCf4v)[10.1073/pnas.96.6.3292](http://dx.doi.org/10.1073/pnas.96.6.3292)

[2. Lorenzo-Orts L, Witthoeft J, Deforges J, Martinez J, Loubéry S, Placzek A, Poirier Y, Hothorn LA, Jaillais Y, Hothorn M (2019) Concerted expression of a cell cycle regulator and a metabolic enzyme from a bicistronic transcript in plants. *Nature plants*, 5(2):184–193. https://doi.org/](http://paperpile.com/b/slGjxJ/XrjRF)[10.1038/s41477-019-0358-3](http://dx.doi.org/10.1038/s41477-019-0358-3)

[3. Zhou Y, Memelink J, Linthorst HJM (2018) An E. coli biosensor for screening of cDNA libraries for isochorismate pyruvate lyase-encoding cDNAs. *Molecular genetics and genomics: MGG*, 293(5):1181–1190. https://doi.org/](http://paperpile.com/b/slGjxJ/nluvH)[10.1007/s00438-018-1450-5](http://dx.doi.org/10.1007/s00438-018-1450-5)

[4. Showalter AM, Keppler B, Lichtenberg J, Gu D, Welch LR (2010) A bioinformatics approach to the identification, classification, and analysis of hydroxyproline-rich glycoproteins. *Plant physiology*, 153(2):485–513. https://doi.org/](http://paperpile.com/b/slGjxJ/VgBMb)[10.1104/pp.110.156554](http://dx.doi.org/10.1104/pp.110.156554)

[5. Steidele CE, Stam R (2021) Multi-omics approach highlights differences between RLP classes in Arabidopsis thaliana. *BMC genomics*, 22(1):557. https://doi.org/](http://paperpile.com/b/slGjxJ/WKgGb)[10.1186/s12864-021-07855-0](http://dx.doi.org/10.1186/s12864-021-07855-0)

[6. Jiang Z, Ge S, Xing L, Han D, Kang Z, Zhang G, Wang X, Wang X, Chen P, Cao A (2013) RLP1.1, a novel wheat receptor-like protein gene, is involved in the defence response against Puccinia striiformis f. sp. tritici. *Journal of experimental botany*, 64(12):3735–3746. https://doi.org/](http://paperpile.com/b/slGjxJ/9nuk4)[10.1093/jxb/ert206](http://dx.doi.org/10.1093/jxb/ert206)

[7. Lee C, Teng Q, Zhong R, Yuan Y, Haghighat M, Ye Z-H (2012) Three Arabidopsis DUF579 domain-containing GXM proteins are methyltransferases catalyzing 4-o-methylation of glucuronic acid on xylan. *Plant & cell physiology*, 53(11):1934–1949. https://doi.org/](http://paperpile.com/b/slGjxJ/ABjSu)[10.1093/pcp/pcs138](http://dx.doi.org/10.1093/pcp/pcs138)

[8. Peralta AG, Venkatachalam S, Stone SC, Pattathil S (2017) Xylan epitope profiling: an enhanced approach to study organ development-dependent changes in xylan structure, biosynthesis, and deposition in plant cell walls. *Biotechnology for biofuels*, 10:245. https://doi.org/](http://paperpile.com/b/slGjxJ/odSBS)[10.1186/s13068-017-0935-5](http://dx.doi.org/10.1186/s13068-017-0935-5)

[9. Samira R, Li B, Kliebenstein D, Li C, Davis E, Gillikin JW, Long TA (2018) The bHLH transcription factor ILR3 modulates multiple stress responses in Arabidopsis. *Plant molecular biology*, 97(4-5):297–309. https://doi.org/](http://paperpile.com/b/slGjxJ/kMYZN)[10.1007/s11103-018-0735-8](http://dx.doi.org/10.1007/s11103-018-0735-8)

[10. Schaller A, Stintzi A, Rivas S, Serrano I, Chichkova NV, Vartapetian AB, Martínez D, Guiamét JJ, Sueldo DJ, Hoorn RAL van der, Ramírez V, Vera P (2018) From structure to function - a family portrait of plant subtilases. *The New phytologist*, 218(3):901–915. https://doi.org/](http://paperpile.com/b/slGjxJ/B2eNx)[10.1111/nph.14582](http://dx.doi.org/10.1111/nph.14582)

[11. Rautengarten C, Usadel B, Neumetzler L, Hartmann J, Büssis D, Altmann T (2008) A subtilisin-like serine protease essential for mucilage release from Arabidopsis seed coats. *The Plant journal: for cell and molecular biology*, 54(3):466–480. https://doi.org/](http://paperpile.com/b/slGjxJ/9F6zv)[10.1111/j.1365-313X.2008.03437.x](http://dx.doi.org/10.1111/j.1365-313X.2008.03437.x)

[12. Hamilton JMU, Simpson DJ, Hyman SC, Ndimba BK, Slabas AR (2003) Ara12 subtilisin-like protease from Arabidopsis thaliana: purification, substrate specificity and tissue localization. *Biochemical Journal*, 370(Pt 1):57–67. https://doi.org/](http://paperpile.com/b/slGjxJ/GF4fh)[10.1042/BJ20021125](http://dx.doi.org/10.1042/BJ20021125)

[13. Nomberg G, Marinov O, Arya GC, Manasherova E, Cohen H (2022) The Key Enzymes in the Suberin Biosynthetic Pathway in Plants: An Update. *Plants*, 11(3)https://doi.org/](http://paperpile.com/b/slGjxJ/Ck3Vw)[10.3390/plants11030392](http://dx.doi.org/10.3390/plants11030392)

[14. Guimarães A, Vieira R, Vieira A (2020) Structure of Leaf Galls in Clusia fluminensis Planch and Triana (Clusiaceae): Sex-Biased Development in a Dioecious Host Plant. *Plants*, 10(1)https://doi.org/](http://paperpile.com/b/slGjxJ/YVXQU)[10.3390/plants10010020](http://dx.doi.org/10.3390/plants10010020)

[15. Hirano T, Kimura S, Sakamoto T, Okamoto A, Nakayama T, Matsuura T, Ikeda Y, Takeda S, Suzuki Y, Ohshima I, Sato MH (2020) Reprogramming of the Developmental Program of Rhus javanica During Initial Stage of Gall Induction by Schlechtendalia chinensis. *Frontiers in plant science*, 11:471. https://doi.org/](http://paperpile.com/b/slGjxJ/AX2Tn)[10.3389/fpls.2020.00471](http://dx.doi.org/10.3389/fpls.2020.00471)

[16. Liu X, Bai J, Huang L, Zhu L, Liu X, Weng N, Reese JC, Harris M, Stuart JJ, Chen M-S (2007) Gene expression of different wheat genotypes during attack by virulent and avirulent Hessian fly (Mayetiola destructor) larvae. *Journal of chemical ecology*, 33(12):2171–2194. https://doi.org/](http://paperpile.com/b/slGjxJ/tZSU9)[10.1007/s10886-007-9382-2](http://dx.doi.org/10.1007/s10886-007-9382-2)

[17. Oliveira DC, Mendonça MS, Moreira ASFP, Lemos-Filho JP, Isaias RMS (2013) Water stress and phenological synchronism between Copaifera langsdorffii (Fabaceae) and multiple galling insects: formation of seasonal patterns. *Journal of plant interactions*, 8(3):225–233. https://doi.org/](http://paperpile.com/b/slGjxJ/EXWFQ)[10.1080/17429145.2012.705339](http://dx.doi.org/10.1080/17429145.2012.705339)

[18. Hou S, Liu D, Huang S, Luo D, Liu Z, Xiang Q, Wang P, Mu R, Han Z, Chen S, Chai J, Shan L, He P (2021) The Arabidopsis MIK2 receptor elicits immunity by sensing a conserved signature from phytocytokines and microbes. *Nature communications*, 12(1):5494. https://doi.org/](http://paperpile.com/b/slGjxJ/7YChK)[10.1038/s41467-021-25580-w](http://dx.doi.org/10.1038/s41467-021-25580-w)

[19. Stahl E, Fernandez Martin A, Glauser G, Guillou M-C, Aubourg S, Renou J-P, Reymond P (2022) The MIK2/SCOOP Signaling System Contributes to Arabidopsis Resistance Against Herbivory by Modulating Jasmonate and Indole Glucosinolate Biosynthesis. *Frontiers in plant science*, 13:852808. https://doi.org/](http://paperpile.com/b/slGjxJ/EV3t0)[10.3389/fpls.2022.852808](http://dx.doi.org/10.3389/fpls.2022.852808)

[20. Van der Does D, Boutrot F, Engelsdorf T, Rhodes J, McKenna JF, Vernhettes S, Koevoets I, Tintor N, Veerabagu M, Miedes E, Segonzac C, Roux M, Breda AS, Hardtke CS, Molina A, Rep M, Testerink C, Mouille G, Höfte H, Hamann T, Zipfel C (2017) The Arabidopsis leucine-rich repeat receptor kinase MIK2/LRR-KISS connects cell wall integrity sensing, root growth and response to abiotic and biotic stresses. *PLoS genetics*, 13(6):e1006832. https://doi.org/](http://paperpile.com/b/slGjxJ/PaABx)[10.1371/journal.pgen.1006832](http://dx.doi.org/10.1371/journal.pgen.1006832)

[21. Coculo D, Lionetti V (2022) The Plant Invertase/Pectin Methylesterase Inhibitor Superfamily. *Frontiers in plant science*, 13:863892. https://doi.org/](http://paperpile.com/b/slGjxJ/50ISO)[10.3389/fpls.2022.863892](http://dx.doi.org/10.3389/fpls.2022.863892)

[22. Xu C, Cao H, Xu E, Zhang S, Hu Y (2018) Genome-Wide Identification of Arabidopsis LBD29 Target Genes Reveals the Molecular Events behind Auxin-Induced Cell Reprogramming during Callus Formation. *Plant & cell physiology*, 59(4):744–755. https://doi.org/](http://paperpile.com/b/slGjxJ/PXPUU)[10.1093/pcp/pcx168](http://dx.doi.org/10.1093/pcp/pcx168)

[23. Wormit A, Usadel B (2018) The Multifaceted Role of Pectin Methylesterase Inhibitors (PMEIs). *International journal of molecular sciences*, 19(10)https://doi.org/](http://paperpile.com/b/slGjxJ/dCw9Y)[10.3390/ijms19102878](http://dx.doi.org/10.3390/ijms19102878)

[24. Sampedro J, Cosgrove DJ (2005) The expansin superfamily. *Genome biology*, 6(12):242. https://doi.org/](http://paperpile.com/b/slGjxJ/RdMoH)[10.1186/gb-2005-6-12-242](http://dx.doi.org/10.1186/gb-2005-6-12-242)

[25. Marowa P, Ding A, Kong Y (2016) Expansins: roles in plant growth and potential applications in crop improvement. *Plant cell reports*, 35(5):949–965. https://doi.org/](http://paperpile.com/b/slGjxJ/xdnaV)[10.1007/s00299-016-1948-4](http://dx.doi.org/10.1007/s00299-016-1948-4)

[26. Baldacci-Cresp F, Sacré P-Y, Twyffels L, Mol A, Vermeersch M, Ziemons E, Hubert P, Pérez-Morga D, El Jaziri M, Almeida Engler J de, Baucher M (2016) Poplar-Root Knot Nematode Interaction: A Model for Perennial Woody Species. *Molecular plant-microbe interactions: MPMI*, 29(7):560–572. https://doi.org/](http://paperpile.com/b/slGjxJ/Ms2TX)[10.1094/MPMI-01-16-0015-R](http://dx.doi.org/10.1094/MPMI-01-16-0015-R)

[27. Jammes F, Lecomte P, Almeida-Engler J de, Bitton F, Martin-Magniette M-L, Renou JP, Abad P, Favery B (2005) Genome-wide expression profiling of the host response to root-knot nematode infection in Arabidopsis. *The Plant journal: for cell and molecular biology*, 44(3):447–458. https://doi.org/](http://paperpile.com/b/slGjxJ/k3a5p)[10.1111/j.1365-313X.2005.02532.x](http://dx.doi.org/10.1111/j.1365-313X.2005.02532.x)

[28. Wieczorek K, Golecki B, Gerdes L, Heinen P, Szakasits D, Durachko DM, Cosgrove DJ, Kreil DP, Puzio PS, Bohlmann H, Grundler FMW (2006) Expansins are involved in the formation of nematode-induced syncytia in roots of Arabidopsis thaliana. *The Plant journal: for cell and molecular biology*, 48(1):98–112. https://doi.org/](http://paperpile.com/b/slGjxJ/vWxiI)[10.1111/j.1365-313X.2006.02856.x](http://dx.doi.org/10.1111/j.1365-313X.2006.02856.x)

[29. Fudali S, Janakowski S, Sobczak M, Griesser M, Grundler FMW, Golinowski W (2008) Two tomato alpha-expansins show distinct spatial and temporal expression patterns during development of nematode-induced syncytia. *Physiologia plantarum*, 132(3):370–383. https://doi.org/](http://paperpile.com/b/slGjxJ/PuAHG)[10.1111/j.1399-3054.2007.01017.x](http://dx.doi.org/10.1111/j.1399-3054.2007.01017.x)

[30. Griesser M, Grundler FMW (2008) Quantification of tomato expansins in nematode feeding sites of cyst and root-knot nematodes. *Journal of plant diseases and protection: scientific journal of the German Phytomedical Society* , 115(6):263–272. https://doi.org/](http://paperpile.com/b/slGjxJ/2A4D4)[10.1007/BF03356275](http://dx.doi.org/10.1007/BF03356275)

[31. Gal TZ, Aussenberg ER, Burdman S, Kapulnik Y, Koltai H (2006) Expression of a plant expansin is involved in the establishment of root knot nematode parasitism in tomato. *Planta*, 224(1):155–162. https://doi.org/](http://paperpile.com/b/slGjxJ/lCh24)[10.1007/s00425-005-0204-x](http://dx.doi.org/10.1007/s00425-005-0204-x)

[32. Zhong R, Peña MJ, Zhou G-K, Nairn CJ, Wood-Jones A, Richardson EA, Morrison WH 3rd, Darvill AG, York WS, Ye Z-H (2005) Arabidopsis fragile fiber8, which encodes a putative glucuronyltransferase, is essential for normal secondary wall synthesis. *The Plant cell*, 17(12):3390–3408. https://doi.org/](http://paperpile.com/b/slGjxJ/iv9tf)[10.1105/tpc.105.035501](http://dx.doi.org/10.1105/tpc.105.035501)

[33. Yuan Y, Teng Q, Zhong R, Haghighat M, Richardson EA, Ye Z-H (2016) Mutations of Arabidopsis TBL32 and TBL33 Affect Xylan Acetylation and Secondary Wall Deposition. *PloS one*, 11(1):e0146460. https://doi.org/](http://paperpile.com/b/slGjxJ/yq0KP)[10.1371/journal.pone.0146460](http://dx.doi.org/10.1371/journal.pone.0146460)

[34. Yuan Y, Teng Q, Zhong R, Ye Z-H (2016) Roles of Arabidopsis TBL34 and TBL35 in xylan acetylation and plant growth. *Plant science: an international journal of experimental plant biology*, 243:120–130. https://doi.org/](http://paperpile.com/b/slGjxJ/rNSMf)[10.1016/j.plantsci.2015.12.007](http://dx.doi.org/10.1016/j.plantsci.2015.12.007)

[35. Hernández-Blanco C, Feng DX, Hu J, Sánchez-Vallet A, Deslandes L, Llorente F, Berrocal-Lobo M, Keller H, Barlet X, Sánchez-Rodríguez C, Anderson LK, Somerville S, Marco Y, Molina A (2007) Impairment of cellulose synthases required for Arabidopsis secondary cell wall formation enhances disease resistance. *The Plant cell*, 19(3):890–903. https://doi.org/](http://paperpile.com/b/slGjxJ/KqGQM)[10.1105/tpc.106.048058](http://dx.doi.org/10.1105/tpc.106.048058)

[36. Barros J, Serk H, Granlund I, Pesquet E (2015) The cell biology of lignification in higher plants. *Annals of botany*, 115(7):1053–1074. https://doi.org/](http://paperpile.com/b/slGjxJ/ONLoY)[10.1093/aob/mcv046](http://dx.doi.org/10.1093/aob/mcv046)

[37. Sibout R, Eudes A, Mouille G, Pollet B, Lapierre C, Jouanin L, Séguin A (2005) CINNAMYL ALCOHOL DEHYDROGENASE-C and -D are the primary genes involved in lignin biosynthesis in the floral stem of Arabidopsis. *The Plant cell*, 17(7):2059–2076. https://doi.org/](http://paperpile.com/b/slGjxJ/Z8B5d)[10.1105/tpc.105.030767](http://dx.doi.org/10.1105/tpc.105.030767)

[38. Bhuiyan NH, Selvaraj G, Wei Y, King J (2009) Gene expression profiling and silencing reveal that monolignol biosynthesis plays a critical role in penetration defence in wheat against powdery mildew invasion. *Journal of experimental botany*, 60(2):509–521. https://doi.org/](http://paperpile.com/b/slGjxJ/YQqiK)[10.1093/jxb/ern290](http://dx.doi.org/10.1093/jxb/ern290)

[39. Lin J-S, Huang X-X, Li Q, Cao Y, Bao Y, Meng X-F, Li Y-J, Fu C, Hou B-K (2016) UDP-glycosyltransferase 72B1 catalyzes the glucose conjugation of monolignols and is essential for the normal cell wall lignification in Arabidopsis thaliana. *The Plant journal: for cell and molecular biology*, 88(1):26–42. https://doi.org/](http://paperpile.com/b/slGjxJ/HCHag)[10.1111/tpj.13229](http://dx.doi.org/10.1111/tpj.13229)

[40. Speeckaert N, El Jaziri M, Baucher M, Behr M (2022) UGT72, a Major Glycosyltransferase Family for Flavonoid and Monolignol Homeostasis in Plants. *Biology*, 11(3)https://doi.org/](http://paperpile.com/b/slGjxJ/LnK6x)[10.3390/biology11030441](http://dx.doi.org/10.3390/biology11030441)

[41. Saint Paul V von, Zhang W, Kanawati B, Geist B, Faus-Kessler T, Schmitt-Kopplin P, Schäffner AR (2011) The Arabidopsis glucosyltransferase UGT76B1 conjugates isoleucic acid and modulates plant defense and senescence. *The Plant cell*, 23(11):4124–4145. https://doi.org/](http://paperpile.com/b/slGjxJ/4e4aP)[10.1105/tpc.111.088443](http://dx.doi.org/10.1105/tpc.111.088443)

[42. Müller K, Levesque-Tremblay G, Fernandes A, Wormit A, Bartels S, Usadel B, Kermode A (2013) Overexpression of a pectin methylesterase inhibitor in Arabidopsis thaliana leads to altered growth morphology of the stem and defective organ separation. *Plant signaling & behavior*, 8(12):e26464. https://doi.org/](http://paperpile.com/b/slGjxJ/qNnJl)[10.4161/psb.26464](http://dx.doi.org/10.4161/psb.26464)

[43. Wen B, Zhang F, Wu X, Li H (2020) Characterization of the Tomato (Solanum lycopersicum) Pectin Methylesterases: Evolution, Activity of Isoforms and Expression During Fruit Ripening. *Frontiers in plant science*, 11:238. https://doi.org/](http://paperpile.com/b/slGjxJ/Vvv6o)[10.3389/fpls.2020.00238](http://dx.doi.org/10.3389/fpls.2020.00238)

[44. Osorio S, Castillejo C, Quesada MA, Medina-Escobar N, Brownsey GJ, Suau R, Heredia A, Botella MA, Valpuesta V (2008) Partial demethylation of oligogalacturonides by pectin methyl esterase 1 is required for eliciting defence responses in wild strawberry (Fragaria vesca). *The Plant journal: for cell and molecular biology*, 54(1):43–55. https://doi.org/](http://paperpile.com/b/slGjxJ/RZZXC)[10.1111/j.1365-313X.2007.03398.x](http://dx.doi.org/10.1111/j.1365-313X.2007.03398.x)

[45. Körner E, Dahl CC von, Bonaventure G, Baldwin IT (2009) Pectin methylesterase NaPME1 contributes to the emission of methanol during insect herbivory and to the elicitation of defence responses in Nicotiana attenuata. *Journal of experimental botany*, 60(9):2631–2640. https://doi.org/](http://paperpile.com/b/slGjxJ/7AQkU)[10.1093/jxb/erp106](http://dx.doi.org/10.1093/jxb/erp106)

[46. Mao D, Yu F, Li J, Van de Poel B, Tan D, Li J, Liu Y, Li X, Dong M, Chen L, Li D, Luan S (2015) FERONIA receptor kinase interacts with S-adenosylmethionine synthetase and suppresses S-adenosylmethionine production and ethylene biosynthesis in Arabidopsis. *Plant, cell & environment*, 38(12):2566–2574. https://doi.org/](http://paperpile.com/b/slGjxJ/mXnC4)[10.1111/pce.12570](http://dx.doi.org/10.1111/pce.12570)

[47. Arimura G-I, Ozawa R, Nishioka T, Boland W, Koch T, Kühnemann F, Takabayashi J (2002) Herbivore-induced volatiles induce the emission of ethylene in neighboring lima bean plants. *The Plant journal: for cell and molecular biology*, 29(1):87–98. https://doi.org/](http://paperpile.com/b/slGjxJ/jXOvt)[10.1046/j.1365-313x.2002.01198.x](http://dx.doi.org/10.1046/j.1365-313x.2002.01198.x)

[48. Xiao W, Hu S, Zou X, Cai R, Liao R, Lin X, Yao R, Guo X (2021) Lectin receptor-like kinase LecRK-VIII.2 is a missing link in MAPK signaling-mediated yield control. *Plant physiology*, 187(1):303–320. https://doi.org/](http://paperpile.com/b/slGjxJ/PAVUE)[10.1093/plphys/kiab241](http://dx.doi.org/10.1093/plphys/kiab241)

[49. Ruzicka K, Strader LC, Bailly A, Yang H, Blakeslee J, Langowski L, Nejedlá E, Fujita H, Itoh H, Syono K, Hejátko J, Gray WM, Martinoia E, Geisler M, Bartel B, Murphy AS, Friml J (2010) Arabidopsis PIS1 encodes the ABCG37 transporter of auxinic compounds including the auxin precursor indole-3-butyric acid. *Proceedings of the National Academy of Sciences of the United States of America*, 107(23):10749–10753. https://doi.org/](http://paperpile.com/b/slGjxJ/e9zk2)[10.1073/pnas.1005878107](http://dx.doi.org/10.1073/pnas.1005878107)

[50. Liu R, Guan C, Fan Z, Ding L, Gao Y, Zhang Q (2021) The mechanism of flower opening and closing times revealed by transcriptome profiling. *Research Square*, https://doi.org/](http://paperpile.com/b/slGjxJ/MntVF)[10.21203/rs.3.rs-1043621/v1](http://dx.doi.org/10.21203/rs.3.rs-1043621/v1)

[51. Gonzalez-Ibeas D, Ibanez V, Perez-Roman E, Borredá C, Terol J, Talon M (2021) Shaping the biology of citrus: II. Genomic determinants of domestication. *The plant genome*, 14(3):e20133. https://doi.org/](http://paperpile.com/b/slGjxJ/jTex8)[10.1002/tpg2.20133](http://dx.doi.org/10.1002/tpg2.20133)

[52. Bailey S, Percy DM, Hefer CA, Cronk QCB (2015) The transcriptional landscape of insect galls: psyllid (Hemiptera) gall formation in Hawaiian Metrosideros polymorpha (Myrtaceae). *BMC genomics*, 16:943. https://doi.org/](http://paperpile.com/b/slGjxJ/3cRuJ)[10.1186/s12864-015-2109-9](http://dx.doi.org/10.1186/s12864-015-2109-9)

[53. Sánchez-López ÁM, Baslam M, De Diego N, Muñoz FJ, Bahaji A, Almagro G, Ricarte-Bermejo A, García-Gómez P, Li J, Humplík JF, Novák O, Spíchal L, Doležal K, Baroja-Fernández E, Pozueta-Romero J (2016) Volatile compounds emitted by diverse phytopathogenic microorganisms promote plant growth and flowering through cytokinin action. *Plant, cell & environment*, 39(12):2592–2608. https://doi.org/](http://paperpile.com/b/slGjxJ/MEsxx)[10.1111/pce.12759](http://dx.doi.org/10.1111/pce.12759)

[54. Abbas M, Peszlen I, Shi R, Kim H, Katahira R, Kafle K, Xiang Z, Huang X, Min D, Mohamadamin M, Yang C, Dai X, Yan X, Park S, Li Y, Kim SH, Davis M, Ralph J, Sederoff RR, Chiang VL, Li Q (2020) Involvement of CesA4, CesA7-A/B and CesA8-A/B in secondary wall formation in Populus trichocarpa wood. *Tree physiology*, 40(1):73–89. https://doi.org/](http://paperpile.com/b/slGjxJ/i4Wby)[10.1093/treephys/tpz020](http://dx.doi.org/10.1093/treephys/tpz020)

[55. Ko J-H, Kim JH, Jayanty SS, Howe GA, Han K-H (2006) Loss of function of COBRA, a determinant of oriented cell expansion, invokes cellular defence responses in Arabidopsis thaliana. *Journal of experimental botany*, 57(12):2923–2936. https://doi.org/](http://paperpile.com/b/slGjxJ/ABwCV)[10.1093/jxb/erl052](http://dx.doi.org/10.1093/jxb/erl052)

[56. Jensen JK, Kim H, Cocuron J-C, Orler R, Ralph J, Wilkerson CG (2011) The DUF579 domain containing proteins IRX15 and IRX15-L affect xylan synthesis in Arabidopsis. *The Plant journal: for cell and molecular biology*, 66(3):387–400. https://doi.org/](http://paperpile.com/b/slGjxJ/DeYtR)[10.1111/j.1365-313X.2010.04475.x](http://dx.doi.org/10.1111/j.1365-313X.2010.04475.x)

[57. Brown D, Wightman R, Zhang Z, Gomez LD, Atanassov I, Bukowski J-P, Tryfona T, McQueen-Mason SJ, Dupree P, Turner S (2011) Arabidopsis genes IRREGULAR XYLEM (IRX15) and IRX15L encode DUF579-containing proteins that are essential for normal xylan deposition in the secondary cell wall. *The Plant journal: for cell and molecular biology*, 66(3):401–413. https://doi.org/](http://paperpile.com/b/slGjxJ/0P3tN)[10.1111/j.1365-313X.2011.04501.x](http://dx.doi.org/10.1111/j.1365-313X.2011.04501.x)

[58. Hussain S, Zhang N, Wang W, Ahmed S, Cheng Y, Chen S, Wang X, Wang Y, Hu X, Wang T, Wang S (2021) Involvement of ABA Responsive SVB Genes in the Regulation of Trichome Formation in Arabidopsis. *International journal of molecular sciences*, 22(13)https://doi.org/](http://paperpile.com/b/slGjxJ/tcRzI)[10.3390/ijms22136790](http://dx.doi.org/10.3390/ijms22136790)

[59. Bastedo DP, Khan M, Martel A, Seto D, Kireeva I, Zhang J, Masud W, Millar D, Lee JY, Lee AH-Y, Gong Y, Santos-Severino A, Guttman DS, Desveaux D (2019) Perturbations of the ZED1 pseudokinase activate plant immunity. *PLoS pathogens*, 15(7):e1007900. https://doi.org/](http://paperpile.com/b/slGjxJ/OTxRf)[10.1371/journal.ppat.1007900](http://dx.doi.org/10.1371/journal.ppat.1007900)

[60. Couto D, Zipfel C (2016) Regulation of pattern recognition receptor signalling in plants. *Nature reviews. Immunology*, 16(9):537–552. https://doi.org/](http://paperpile.com/b/slGjxJ/iB7so)[10.1038/nri.2016.77](http://dx.doi.org/10.1038/nri.2016.77)

[61. Wang G, Roux B, Feng F, Guy E, Li L, Li N, Zhang X, Lautier M, Jardinaud M-F, Chabannes M, Arlat M, Chen S, He C, Noël LD, Zhou J-M (2015) The Decoy Substrate of a Pathogen Effector and a Pseudokinase Specify Pathogen-Induced Modified-Self Recognition and Immunity in Plants. *Cell host & microbe*, 18(3):285–295. https://doi.org/](http://paperpile.com/b/slGjxJ/RIbTP)[10.1016/j.chom.2015.08.004](http://dx.doi.org/10.1016/j.chom.2015.08.004)

[62. Bozorov TA, Pandey SP, Dinh ST, Kim S-G, Heinrich M, Gase K, Baldwin IT (2012) DICER-like proteins and their role in plant-herbivore interactions in Nicotiana attenuata. *Journal of integrative plant biology*, 54(3):189–206. https://doi.org/](http://paperpile.com/b/slGjxJ/ygiwG)[10.1111/j.1744-7909.2012.01104.x](http://dx.doi.org/10.1111/j.1744-7909.2012.01104.x)

[63. Mirabella R, Rauwerda H, Allmann S, Scala A, Spyropoulou EA, Vries M de, Boersma MR, Breit TM, Haring MA, Schuurink RC (2015) WRKY40 and WRKY6 act downstream of the green leaf volatile E-2-hexenal in Arabidopsis. *The Plant journal: for cell and molecular biology*, 83(6):1082–1096. https://doi.org/](http://paperpile.com/b/slGjxJ/5CIux)[10.1111/tpj.12953](http://dx.doi.org/10.1111/tpj.12953)

[64. Li J, Zhu L, Hull JJ, Liang S, Daniell H, Jin S, Zhang X (2016) Transcriptome analysis reveals a comprehensive insect resistance response mechanism in cotton to infestation by the phloem feeding insect Bemisia tabaci (whitefly). *Plant biotechnology journal*, 14(10):1956–1975. https://doi.org/](http://paperpile.com/b/slGjxJ/qj8BU)[10.1111/pbi.12554](http://dx.doi.org/10.1111/pbi.12554)

[65. Steenbergen M (2022) Gene regulatory network induced by Western flower thrips in Arabidopsis. https://doi.org/](http://paperpile.com/b/slGjxJ/9wKRc)[10.33540/1291](http://dx.doi.org/10.33540/1291)

[66. Schweizer F, Bodenhausen N, Lassueur S, Masclaux FG, Reymond P (2013) Differential Contribution of Transcription Factors to Arabidopsis thaliana Defense Against Spodoptera littoralis. *Frontiers in plant science*, 4:13. https://doi.org/](http://paperpile.com/b/slGjxJ/AMdDU)[10.3389/fpls.2013.00013](http://dx.doi.org/10.3389/fpls.2013.00013)

[67. Appel HM, Fescemyer H, Ehlting J, Weston D, Rehrig E, Joshi T, Xu D, Bohlmann J, Schultz J (2014) Transcriptional responses of Arabidopsis thaliana to chewing and sucking insect herbivores. *Frontiers in plant science*, 5:565. https://doi.org/](http://paperpile.com/b/slGjxJ/PCKwW)[10.3389/fpls.2014.00565](http://dx.doi.org/10.3389/fpls.2014.00565)

[68. Macharia TN, Bellieny-Rabelo D, Moleleki LN (2020) Transcriptome Profiling of Potato (Solanum tuberosum L.) Responses to Root-Knot Nematode (Meloidogyne javanica) Infestation during A Compatible Interaction. *Microorganisms*, 8(9)https://doi.org/](http://paperpile.com/b/slGjxJ/MXOFa)[10.3390/microorganisms8091443](http://dx.doi.org/10.3390/microorganisms8091443)

[69. Chinnapandi B, Bucki P, Braun Miyara S (2017) SlWRKY45, nematode-responsive tomato WRKY gene, enhances susceptibility to the root knot nematode; M. javanica infection. *Plant signaling & behavior*, 12(12):e1356530. https://doi.org/](http://paperpile.com/b/slGjxJ/ILvP)[10.1080/15592324.2017.1356530](http://dx.doi.org/10.1080/15592324.2017.1356530)

[70. Kerchev PI (2011) Local and systemic responses to Myzus persicae in Arabidopsis. A role for redox components.](http://paperpile.com/b/slGjxJ/1qv28) <https://etheses.whiterose.ac.uk/2404/>

[71. Imran QM, Falak N, Hussain A, Mun B-G, Sharma A, Lee S-U, Kim K-M, Yun B-W (2016) Nitric Oxide Responsive Heavy Metal-Associated Gene AtHMAD1 Contributes to Development and Disease Resistance in Arabidopsis thaliana. *Frontiers in plant science*, 7:1712. https://doi.org/](http://paperpile.com/b/slGjxJ/y63Kf)[10.3389/fpls.2016.01712](http://dx.doi.org/10.3389/fpls.2016.01712)

[72. Mata-Pérez C, Spoel SH (2019) Thioredoxin-mediated redox signalling in plant immunity. *Plant science: an international journal of experimental plant biology*, 279:27–33. https://doi.org/](http://paperpile.com/b/slGjxJ/SmYVt)[10.1016/j.plantsci.2018.05.001](http://dx.doi.org/10.1016/j.plantsci.2018.05.001)

[73. Alves Teixeira M (2017) Root-Knot Nematode-Triggered Defense Responses in Arabidopsis Thaliana during Early Stages of Parasitism.](http://paperpile.com/b/slGjxJ/bO1nD) <http://ezproxy.msu.edu/login?url=https://www.proquest.com/dissertations-theses/root-knot-nematode-triggered-defense-responses-i/docview/1911954123/se-2>

[74. Barah P, Winge P, Kusnierczyk A, Tran DH, Bones AM (2013) Molecular signatures in Arabidopsis thaliana in response to insect attack and bacterial infection. *PloS one*, 8(3):e58987. https://doi.org/](http://paperpile.com/b/slGjxJ/fI1rP)[10.1371/journal.pone.0058987](http://dx.doi.org/10.1371/journal.pone.0058987)

[75. Kuznetsov, Shevyakova (2007) Polyamines and stress tolerance of plants. *Plant stress*,](http://paperpile.com/b/slGjxJ/oeyvx) <https://www.academia.edu/download/45454312/PS_1150-71o.pdf>

[76. Cowley T, Walters DR (2002) Polyamine Metabolism in an Incompatible Interaction between Barley and the Powdery Mildew Fungus, Blumeria graminis f. sp. hordei. *Phytopathologische Zeitschrift. Journal of phytopathology*, 150(11-12):581–586. https://doi.org/](http://paperpile.com/b/slGjxJ/0wECX)[10.1046/j.1439-0434.2002.00816.x](http://dx.doi.org/10.1046/j.1439-0434.2002.00816.x)

[77. Kot I, Sempruch C, Chrzanowski G, Czerniewicz P (2019) The effect of leaf galls of Cynipidae on accumulation and biosynthesis of plant amines in oak trees. *Biochemical systematics and ecology*, 83:26–32. https://doi.org/](http://paperpile.com/b/slGjxJ/nE3bm)[10.1016/j.bse.2018.12.016](http://dx.doi.org/10.1016/j.bse.2018.12.016)

[78. Sempruch C, Horbowicz M, Kosson R, Leszczyński B (2012) Biochemical interactions between triticale (Triticosecale; Poaceae) amines and bird cherry-oat aphid (Rhopalosiphum padi; Aphididae). *Biochemical systematics and ecology*, 40:162–168. https://doi.org/](http://paperpile.com/b/slGjxJ/gnNQ1)[10.1016/j.bse.2011.10.004](http://dx.doi.org/10.1016/j.bse.2011.10.004)

[79. Sempruch C, Goławska S, Osiński P, Leszczyński B, Czerniewicz P, Sytykiewicz H, Matok H (2016) Influence of selected plant amines on probing behaviour of bird cherry-oat aphid (Rhopalosiphum padi L.). *Bulletin of entomological research*, 106(3):368–377. https://doi.org/](http://paperpile.com/b/slGjxJ/LPwdz)[10.1017/S0007485316000055](http://dx.doi.org/10.1017/S0007485316000055)

[80. Woo JY, Jeong KJ, Kim YJ, Paek K-H (2016) CaLecRK-S.5, a pepper L-type lectin receptor kinase gene, confers broad-spectrum resistance by activating priming. *Journal of experimental botany*, 67(19):5725–5741. https://doi.org/](http://paperpile.com/b/slGjxJ/kYFXp)[10.1093/jxb/erw336](http://dx.doi.org/10.1093/jxb/erw336)

[81. Sharon L Identification and Characterization of PAMP/NAMP Receptors Recognizing Nematodes.](http://paperpile.com/b/slGjxJ/Lf9Fu) <https://libstore.ugent.be/fulltxt/RUG01/002/862/997/RUG01-002862997_2020_0001_AC.pdf>

[82. Feys BJ, Moisan LJ, Newman MA, Parker JE (2001) Direct interaction between the Arabidopsis disease resistance signaling proteins, EDS1 and PAD4. *The EMBO journal*, 20(19):5400–5411. https://doi.org/](http://paperpile.com/b/slGjxJ/2EZGK)[10.1093/emboj/20.19.5400](http://dx.doi.org/10.1093/emboj/20.19.5400)

[83. Du B, Zhang W, Liu B, Hu J, Wei Z, Shi Z, He R, Zhu L, Chen R, Han B, He G (2009) Identification and characterization of *Bph14*, a gene conferring resistance to brown planthopper in rice. *Proceedings of the National Academy of Sciences of the United States of America*, 106(52):22163–22168. https://doi.org/](http://paperpile.com/b/slGjxJ/YeCJN)[10.1073/pnas.0912139106](http://dx.doi.org/10.1073/pnas.0912139106)

[84. Vlot AC, Dempsey DA, Klessig DF (2009) Salicylic Acid, a multifaceted hormone to combat disease. *Annual review of phytopathology*, 47:177–206. https://doi.org/](http://paperpile.com/b/slGjxJ/8G0mD)[10.1146/annurev.phyto.050908.135202](http://dx.doi.org/10.1146/annurev.phyto.050908.135202)

[85. Reymond P (2013) Perception, signaling and molecular basis of oviposition-mediated plant responses. *Planta*, 238(2):247–258. https://doi.org/](http://paperpile.com/b/slGjxJ/yAwYI)[10.1007/s00425-013-1908-y](http://dx.doi.org/10.1007/s00425-013-1908-y)

[86. Gouhier-Darimont C, Schmiesing A, Bonnet C, Lassueur S, Reymond P (2013) Signalling of Arabidopsis thaliana response to Pieris brassicae eggs shares similarities with PAMP-triggered immunity. *Journal of experimental botany*, 64(2):665–674. https://doi.org/](http://paperpile.com/b/slGjxJ/cgG3M)[10.1093/jxb/ers362](http://dx.doi.org/10.1093/jxb/ers362)

[87. Chen Y-L, Lee C-Y, Cheng K-T, Chang W-H, Huang R-N, Nam HG, Chen Y-R (2014) Quantitative peptidomics study reveals that a wound-induced peptide from PR-1 regulates immune signaling in tomato. *The Plant cell*, 26(10):4135–4148. https://doi.org/](http://paperpile.com/b/slGjxJ/E84Gy)[10.1105/tpc.114.131185](http://dx.doi.org/10.1105/tpc.114.131185)

[88. Perdiguero P, Sobrino-Plata J, Venturas M, Martín JA, Gil L, Collada C (2018) Gene expression trade-offs between defence and growth in English elm induced by Ophiostoma novo-ulmi. *Plant, cell & environment*, 41(1):198–214. https://doi.org/](http://paperpile.com/b/slGjxJ/9WX2Z)[10.1111/pce.13085](http://dx.doi.org/10.1111/pce.13085)

[89. Nintemann SJ, Vik D, Svozil J, Bak M, Baerenfaller K, Burow M, Halkier BA (2017) Unravelling Protein-Protein Interaction Networks Linked to Aliphatic and Indole Glucosinolate Biosynthetic Pathways in Arabidopsis. *Frontiers in plant science*, 8:2028. https://doi.org/](http://paperpile.com/b/slGjxJ/6XNaw)[10.3389/fpls.2017.02028](http://dx.doi.org/10.3389/fpls.2017.02028)

[90. Ngou P (2020) Roles and mechanisms of effector-triggered immunity in plant disease resistance.](http://paperpile.com/b/slGjxJ/Ph0Gq) <https://ueaeprints.uea.ac.uk/id/eprint/81783/>

[91. Ralph SG, Yueh H, Friedmann M, Aeschliman D, Zeznik JA, Nelson CC, Butterfield YSN, Kirkpatrick R, Liu J, Jones SJM, Marra MA, Douglas CJ, Ritland K, Bohlmann J (2006) Conifer defence against insects: microarray gene expression profiling of Sitka spruce (Picea sitchensis) induced by mechanical wounding or feeding by spruce budworms (Choristoneura occidentalis) or white pine weevils (Pissodes strobi) reveals large-scale changes of the host transcriptome. *Plant, cell & environment*, 29(8):1545–1570. https://doi.org/](http://paperpile.com/b/slGjxJ/sMgni)[10.1111/j.1365-3040.2006.01532.x](http://dx.doi.org/10.1111/j.1365-3040.2006.01532.x)

[92. Duhlian L, Koramutla MK, Subramanian S, Chamola R, Bhattacharya R (2020) Comparative transcriptomics revealed differential regulation of defense related genes in Brassica juncea leading to successful and unsuccessful infestation by aphid species. *Scientific reports*, 10(1):10583. https://doi.org/](http://paperpile.com/b/slGjxJ/6Fx4o)[10.1038/s41598-020-66217-0](http://dx.doi.org/10.1038/s41598-020-66217-0)

[93. Zheng S, Liu W, Luo J, Wang L, Zhu X, Gao X, Hua H, Cui J (2022) Helicoverpa armigera herbivory negatively impacts Aphis gossypii populations via inducible metabolic changes. *Pest management science*, 78(6):2357–2369. https://doi.org/](http://paperpile.com/b/slGjxJ/tPaZO)[10.1002/ps.6865](http://dx.doi.org/10.1002/ps.6865)

[94. Kovalchuk A, Raffaello T, Jaber E, Keriö S, Ghimire R, Lorenz WW, Dean JFD, Holopainen JK, Asiegbu FO (2015) Activation of defence pathways in Scots pine bark after feeding by pine weevil (Hylobius abietis). *BMC genomics*, 16:352. https://doi.org/](http://paperpile.com/b/slGjxJ/KC3AB)[10.1186/s12864-015-1546-9](http://dx.doi.org/10.1186/s12864-015-1546-9)

[95. Bindschedler LV, Dewdney J, Blee KA, Stone JM, Asai T, Plotnikov J, Denoux C, Hayes T, Gerrish C, Davies DR, Ausubel FM, Bolwell GP (2006) Peroxidase-dependent apoplastic oxidative burst in Arabidopsis required for pathogen resistance. *The Plant journal: for cell and molecular biology*, 47(6):851–863. https://doi.org/](http://paperpile.com/b/slGjxJ/kqJpv)[10.1111/j.1365-313X.2006.02837.x](http://dx.doi.org/10.1111/j.1365-313X.2006.02837.x)

[96. Schön M, Töller A, Diezel C, Roth C, Westphal L, Wiermer M, Somssich IE (2013) Analyses of wrky18 wrky40 plants reveal critical roles of SA/EDS1 signaling and indole-glucosinolate biosynthesis for Golovinomyces orontii resistance and a loss-of resistance towards Pseudomonas syringae pv. tomato AvrRPS4. *Molecular plant-microbe interactions: MPMI*, 26(7):758–767. https://doi.org/](http://paperpile.com/b/slGjxJ/dxoDZ)[10.1094/MPMI-11-12-0265-R](http://dx.doi.org/10.1094/MPMI-11-12-0265-R)

[97. Iwase A, Kondo Y, Laohavisit A, Takebayashi A, Ikeuchi M, Matsuoka K, Asahina M, Mitsuda N, Shirasu K, Fukuda H, Sugimoto K (2021) WIND transcription factors orchestrate wound-induced callus formation, vascular reconnection and defense response in Arabidopsis. *The New phytologist*, 232(2):734–752. https://doi.org/](http://paperpile.com/b/slGjxJ/z8uib)[10.1111/nph.17594](http://dx.doi.org/10.1111/nph.17594)

[98. Xu X, Chen C, Fan B, Chen Z (2006) Physical and functional interactions between pathogen-induced Arabidopsis WRKY18, WRKY40, and WRKY60 transcription factors. *The Plant cell*, 18(5):1310–1326. https://doi.org/](http://paperpile.com/b/slGjxJ/KHrVL)[10.1105/tpc.105.037523](http://dx.doi.org/10.1105/tpc.105.037523)

[99. Chen X, Li C, Wang H, Guo Z (2019) WRKY transcription factors: evolution, binding, and action. *Phytopathology Research*, 1(1):1–15. https://doi.org/](http://paperpile.com/b/slGjxJ/r6M8F)[10.1186/s42483-019-0022-x](http://dx.doi.org/10.1186/s42483-019-0022-x)

[100. Hart SV, Kogan M, Paxton JD (1983) Effect of soybean phytoalexins on the herbivorous insects mexican bean beetle and soybean looper. *Journal of chemical ecology*, 9(6):657–672. https://doi.org/](http://paperpile.com/b/slGjxJ/ghBtQ)[10.1007/BF00988774](http://dx.doi.org/10.1007/BF00988774)

[101. Russell GB, Sutherland ORW, Hutchins RFN, Christmas PE (1978) Vestitol: A phytoalexin with insect feeding-deterrent activity. *Journal of chemical ecology*, 4(5):571–579. https://doi.org/](http://paperpile.com/b/slGjxJ/OJ2TR)[10.1007/BF00988921](http://dx.doi.org/10.1007/BF00988921)

[102. Hohenstein JD, Studham ME, Klein A, Kovinich N, Barry K, Lee Y-J, MacIntosh GC (2019) Transcriptional and Chemical Changes in Soybean Leaves in Response to Long-Term Aphid Colonization. *Frontiers in plant science*, 10:310. https://doi.org/](http://paperpile.com/b/slGjxJ/XpxQC)[10.3389/fpls.2019.00310](http://dx.doi.org/10.3389/fpls.2019.00310)

[103. Sylvestre-Gonon E, Law SR, Schwartz M, Robe K, Keech O, Didierjean C, Dubos C, Rouhier N, Hecker A (2019) Functional, Structural and Biochemical Features of Plant Serinyl-Glutathione Transferases. *Frontiers in plant science*, 10:608. https://doi.org/](http://paperpile.com/b/slGjxJ/mhZgg)[10.3389/fpls.2019.00608](http://dx.doi.org/10.3389/fpls.2019.00608)

[104. Kusnierczyk A, Winge P, Midelfart H, Armbruster WS, Rossiter JT, Bones AM (2007) Transcriptional responses of Arabidopsis thaliana ecotypes with different glucosinolate profiles after attack by polyphagous Myzus persicae and oligophagous Brevicoryne brassicae. *Journal of experimental botany*, 58(10):2537–2552. https://doi.org/](http://paperpile.com/b/slGjxJ/kHe9K)[10.1093/jxb/erm043](http://dx.doi.org/10.1093/jxb/erm043)

[105. Sytykiewicz H, Chrzanowski G, Czerniewicz P, Sprawka I, Łukasik I, Goławska S, Sempruch C (2014) Expression profiling of selected glutathione transferase genes in Zea mays (L.) seedlings infested with cereal aphids. *PloS one*, 9(11):e111863. https://doi.org/](http://paperpile.com/b/slGjxJ/SdktC)[10.1371/journal.pone.0111863](http://dx.doi.org/10.1371/journal.pone.0111863)

[106. Ockels FS, Eyles A, McPherson BA, Wood DL, Bonello P (2007) Phenolic chemistry of coast live oak response to Phytophthora ramorum infection. *Journal of chemical ecology*, 33(9):1721–1732. https://doi.org/](http://paperpile.com/b/slGjxJ/kLbir)[10.1007/s10886-007-9332-z](http://dx.doi.org/10.1007/s10886-007-9332-z)

[107. Rehman HM, Nawaz MA, Shah ZH, Ludwig-Müller J, Chung G, Ahmad MQ, Yang SH, Lee SI (2018) Comparative genomic and transcriptomic analyses of Family-1 UDP glycosyltransferase in three Brassica species and Arabidopsis indicates stress-responsive regulation. *Scientific reports*, 8(1):1875. https://doi.org/](http://paperpile.com/b/slGjxJ/gdUHo)[10.1038/s41598-018-19535-3](http://dx.doi.org/10.1038/s41598-018-19535-3)

[108. Nathoo N (2015) Identification of Putative Plant Defense Genes Using a Novel Hydroponic Co-Cultivation Technique for Studying Plant-Pathogen Interaction.](http://paperpile.com/b/slGjxJ/GA6B0) <https://ir.lib.uwo.ca/etd/2883/>

[109. Stahl E, Brillatz T, Ferreira Queiroz E, Marcourt L, Schmiesing A, Hilfiker O, Riezman I, Riezman H, Wolfender J-L, Reymond P (2020) Phosphatidylcholines from Pieris brassicae eggs activate an immune response in Arabidopsis. *eLife*, 9https://doi.org/](http://paperpile.com/b/slGjxJ/CcHt6)[10.7554/eLife.60293](http://dx.doi.org/10.7554/eLife.60293)

[110. George Thompson AM, Iancu CV, Neet KE, Dean JV, Choe J-Y (2017) Differences in salicylic acid glucose conjugations by UGT74F1 and UGT74F2 from Arabidopsis thaliana. *Scientific reports*, 7:46629. https://doi.org/](http://paperpile.com/b/slGjxJ/Gfjja)[10.1038/srep46629](http://dx.doi.org/10.1038/srep46629)

[111. Haroth S, Feussner K, Kelly AA, Zienkiewicz K, Shaikhqasem A, Herrfurth C, Feussner I (2019) The glycosyltransferase UGT76E1 significantly contributes to 12-O-glucopyranosyl-jasmonic acid formation in wounded Arabidopsis thaliana leaves. *The Journal of biological chemistry*, 294(25):9858–9872. https://doi.org/](http://paperpile.com/b/slGjxJ/zoZXB)[10.1074/jbc.RA119.007600](http://dx.doi.org/10.1074/jbc.RA119.007600)

[112. Quiel JA, Bender J (2003) Glucose conjugation of anthranilate by the Arabidopsis UGT74F2 glucosyltransferase is required for tryptophan mutant blue fluorescence. *The Journal of biological chemistry*, 278(8):6275–6281. https://doi.org/](http://paperpile.com/b/slGjxJ/mtZzd)[10.1074/jbc.M211822200](http://dx.doi.org/10.1074/jbc.M211822200)

[113. Lim E-K, Doucet CJ, Li Y, Elias L, Worrall D, Spencer SP, Ross J, Bowles DJ (2002) The activity of Arabidopsis glycosyltransferases toward salicylic acid, 4-hydroxybenzoic acid, and other benzoates. *The Journal of biological chemistry*, 277(1):586–592. https://doi.org/](http://paperpile.com/b/slGjxJ/vcijh)[10.1074/jbc.M109287200](http://dx.doi.org/10.1074/jbc.M109287200)

[114. Cartwright AM, Lim E-K, Kleanthous C, Bowles DJ (2008) A kinetic analysis of regiospecific glucosylation by two glycosyltransferases of Arabidopsis thaliana: domain swapping to introduce new activities. *The Journal of biological chemistry*, 283(23):15724–15731. https://doi.org/](http://paperpile.com/b/slGjxJ/mFxLO)[10.1074/jbc.M801983200](http://dx.doi.org/10.1074/jbc.M801983200)

[115. Little D, Gouhier-Darimont C, Bruessow F, Reymond P (2007) Oviposition by pierid butterflies triggers defense responses in Arabidopsis. *Plant physiology*, 143(2):784–800. https://doi.org/](http://paperpile.com/b/slGjxJ/dxAiy)[10.1104/pp.106.090837](http://dx.doi.org/10.1104/pp.106.090837)

[116. Chhajed S, Mostafa I, He Y, Abou-Hashem M, El-Domiaty M, Chen S (2020) Glucosinolate Biosynthesis and the Glucosinolate–Myrosinase System in Plant Defense. *Agronomy*, 10(11):1786. https://doi.org/](http://paperpile.com/b/slGjxJ/fX4x4)[10.3390/agronomy10111786](http://dx.doi.org/10.3390/agronomy10111786)

[117. Asano T, Kimura M, Nishiuchi T (2012) The defense response in Arabidopsis thaliana against Fusarium sporotrichioides. *Proteome science*, 10(1):61. https://doi.org/](http://paperpile.com/b/slGjxJ/KwWiu)[10.1186/1477-5956-10-61](http://dx.doi.org/10.1186/1477-5956-10-61)

[118. Alfenas-Zerbini P, Maia IG, Fávaro RD, Cascardo JCM, Brommonschenkel SH, Zerbini FM (2009) Genome-wide analysis of differentially expressed genes during the early stages of tomato infection by a potyvirus. *Molecular plant-microbe interactions: MPMI*, 22(3):352–361. https://doi.org/](http://paperpile.com/b/slGjxJ/3kHLx)[10.1094/MPMI-22-3-0352](http://dx.doi.org/10.1094/MPMI-22-3-0352)

[119. Fatouros NE, Broekgaarden C, Bukovinszkine’Kiss G, Loon JJA van, Mumm R, Huigens ME, Dicke M, Hilker M (2008) Male-derived butterfly anti-aphrodisiac mediates induced indirect plant defense. *Proceedings of the National Academy of Sciences of the United States of America*, 105(29):10033–10038. https://doi.org/](http://paperpile.com/b/slGjxJ/8x2uq)[10.1073/pnas.0707809105](http://dx.doi.org/10.1073/pnas.0707809105)

[120. Takekawa M, Posas F, Saito H (1997) A human homolog of the yeast Ssk2/Ssk22 MAP kinase kinase kinases, MTK1, mediates stress-induced activation of the p38 and JNK pathways. *The EMBO journal*, 16(16):4973–4982. https://doi.org/](http://paperpile.com/b/slGjxJ/OsBOX)[10.1093/emboj/16.16.4973](http://dx.doi.org/10.1093/emboj/16.16.4973)

[121. Niu F, Wang B, Wu F, Yan J, Li L, Wang C, Wang Y, Yang B, Jiang Y-Q (2014) Canola (Brassica napus L.) NAC103 transcription factor gene is a novel player inducing reactive oxygen species accumulation and cell death in plants. *Biochemical and biophysical research communications*, 454(1):30–35. https://doi.org/](http://paperpile.com/b/slGjxJ/bGdDI)[10.1016/j.bbrc.2014.10.057](http://dx.doi.org/10.1016/j.bbrc.2014.10.057)

[122. Kosma DK, Nemacheck JA, Jenks MA, Williams CE (2010) Changes in properties of wheat leaf cuticle during interactions with Hessian fly. *The Plant journal: for cell and molecular biology*, 63(1):31–43. https://doi.org/](http://paperpile.com/b/slGjxJ/bZj0E)[10.1111/j.1365-313X.2010.04229.x](http://dx.doi.org/10.1111/j.1365-313X.2010.04229.x)

[123. Weidenbach D, Jansen M, Bodewein T, Nagel KA, Schaffrath U (2015) Shoot and root phenotyping of the barley mutant kcs6 (3-ketoacyl-CoA synthase6) depleted in epicuticular waxes under water limitation. *Plant signaling & behavior*, 10(4):1–3. https://doi.org/](http://paperpile.com/b/slGjxJ/QXcax)[10.1080/15592324.2014.1003752](http://dx.doi.org/10.1080/15592324.2014.1003752)

[124. Body MJA, Appel HM, Edger PP, Schultz JC (2019) A gall-forming insect manipulates hostplant phytohormone synthesis, concentrations, and signaling. *bioRxiv*, :658823. https://doi.org/](http://paperpile.com/b/slGjxJ/9TU9d)[10.1101/658823](http://dx.doi.org/10.1101/658823)

[125. Cao T, Lahiri I, Singh V, Louis J, Shah J, Ayre BG (2013) Metabolic engineering of raffinose-family oligosaccharides in the phloem reveals alterations in carbon partitioning and enhances resistance to green peach aphid. *Frontiers in plant science*, 4:263. https://doi.org/](http://paperpile.com/b/slGjxJ/6slKf)[10.3389/fpls.2013.00263](http://dx.doi.org/10.3389/fpls.2013.00263)

[126. Berenbaum MR (1995) Turnabout is fair play: Secondary roles for primary compounds. *Journal of chemical ecology*, 21(7):925–940. https://doi.org/](http://paperpile.com/b/slGjxJ/sbGoP)[10.1007/BF02033799](http://dx.doi.org/10.1007/BF02033799)

[127. Moran N, Hamilton WD (1980) Low nutritive quality as defense against herbivores. *Journal of theoretical biology*, 86(2):247–254. https://doi.org/](http://paperpile.com/b/slGjxJ/8f6Vu)[10.1016/0022-5193(80)90004-1](http://dx.doi.org/10.1016/0022-5193(80)90004-1)

[128. Yan A, Borg M, Berger F, Chen Z (2020) The atypical histone variant H3.15 promotes callus formation in Arabidopsis thaliana. *Development* , 147(11)https://doi.org/](http://paperpile.com/b/slGjxJ/WySrm)[10.1242/dev.184895](http://dx.doi.org/10.1242/dev.184895)

[129. Levy A, Erlanger M, Rosenthal M, Epel BL (2007) A plasmodesmata-associated beta-1,3-glucanase in Arabidopsis. *The Plant journal: for cell and molecular biology*, 49(4):669–682. https://doi.org/](http://paperpile.com/b/slGjxJ/cbppl)[10.1111/j.1365-313X.2006.02986.x](http://dx.doi.org/10.1111/j.1365-313X.2006.02986.x)

[130. Johnston M (2021) The proteins found at plasmodesmata and the interactions between them.](http://paperpile.com/b/slGjxJ/hTdCw) <https://ueaeprints.uea.ac.uk/id/eprint/81897/>

[131. Berardini TZ, Reiser L, Li D, Mezheritsky Y, Muller R, Strait E, Huala E (2015) The Arabidopsis information resource: Making and mining the “gold standard” annotated reference plant genome. *Genesis* , 53(8):474–485. https://doi.org/](http://paperpile.com/b/slGjxJ/6F9rp)[10.1002/dvg.22877](http://dx.doi.org/10.1002/dvg.22877)

[132. Gruner K, Griebel T, Návarová H, Attaran E, Zeier J (2013) Reprogramming of plants during systemic acquired resistance. *Frontiers in plant science*, 4:252. https://doi.org/](http://paperpile.com/b/slGjxJ/fagsN)[10.3389/fpls.2013.00252](http://dx.doi.org/10.3389/fpls.2013.00252)

[133. Deeken R, Engelmann JC, Efetova M, Czirjak T, Müller T, Kaiser WM, Tietz O, Krischke M, Mueller MJ, Palme K, Dandekar T, Hedrich R (2006) An integrated view of gene expression and solute profiles of Arabidopsis tumors: a genome-wide approach. *The Plant cell*, 18(12):3617–3634. https://doi.org/](http://paperpile.com/b/slGjxJ/hakCx)[10.1105/tpc.106.044743](http://dx.doi.org/10.1105/tpc.106.044743)

[134. Ranf S, Eschen-Lippold L, Fröhlich K, Westphal L, Scheel D, Lee J (2014) Microbe-associated molecular pattern-induced calcium signaling requires the receptor-like cytoplasmic kinases, PBL1 and BIK1. *BMC plant biology*, 14:374. https://doi.org/](http://paperpile.com/b/slGjxJ/mulEp)[10.1186/s12870-014-0374-4](http://dx.doi.org/10.1186/s12870-014-0374-4)

[135. Chinchilla D, Bauer Z, Regenass M, Boller T, Felix G (2006) The Arabidopsis receptor kinase FLS2 binds flg22 and determines the specificity of flagellin perception. *The Plant cell*, 18(2):465–476. https://doi.org/](http://paperpile.com/b/slGjxJ/89nY1)[10.1105/tpc.105.036574](http://dx.doi.org/10.1105/tpc.105.036574)

[136. Huang C, Sede AR, Mutterer J, Boutant E, Heinlein M (2021) Suppression of a dsRNA-induced plant immunity pathway by viral movement protein. *bioRxiv*, :2021.10.30.466425. https://doi.org/](http://paperpile.com/b/slGjxJ/KBmUj)[10.1101/2021.10.30.466425](http://dx.doi.org/10.1101/2021.10.30.466425)

[137. Zhang J, Li W, Xiang T, Liu Z, Laluk K, Ding X, Zou Y, Gao M, Zhang X, Chen S, Mengiste T, Zhang Y, Zhou J-M (2010) Receptor-like cytoplasmic kinases integrate signaling from multiple plant immune receptors and are targeted by a Pseudomonas syringae effector. *Cell host & microbe*, 7(4):290–301. https://doi.org/](http://paperpile.com/b/slGjxJ/ER1Hk)[10.1016/j.chom.2010.03.007](http://dx.doi.org/10.1016/j.chom.2010.03.007)

[138. Irieda H, Inoue Y, Mori M, Yamada K, Oshikawa Y, Saitoh H, Uemura A, Terauchi R, Kitakura S, Kosaka A, Singkaravanit-Ogawa S, Takano Y (2019) Conserved fungal effector suppresses PAMP-triggered immunity by targeting plant immune kinases. *Proceedings of the National Academy of Sciences of the United States of America*, 116(2):496–505. https://doi.org/](http://paperpile.com/b/slGjxJ/PmCPR)[10.1073/pnas.1807297116](http://dx.doi.org/10.1073/pnas.1807297116)

[139. Okeke G (2012) Pine transcriptomics : RNA-Seq data analysis of Scots pine (Pinus sylvestris) seedlings subjected to a wounding experiment.](http://paperpile.com/b/slGjxJ/TYAgB) <https://helda.helsinki.fi/handle/10138/36529>

[140. Shouan L (2014) The role of Arabidopsis WRKY33 in modulating host immunity towards the necrotroph Botrytis cinerea.](http://paperpile.com/b/slGjxJ/1Je39) <https://kups.ub.uni-koeln.de/6239/>

[141. Schenk PM, Thomas-Hall SR, Nguyen AV, Manners JM, Kazan K, Spangenberg G (2008) Identification of plant defence genes in canola using Arabidopsis cDNA microarrays. *Plant biology* , 10(5):539–547. https://doi.org/](http://paperpile.com/b/slGjxJ/DDCDr)[10.1111/j.1438-8677.2008.00056.x](http://dx.doi.org/10.1111/j.1438-8677.2008.00056.x)

[142. Czobor Á, Hajdinák P, Szarka A (2017) Rapid ascorbate response to bacterial elicitor treatment in Arabidopsis thaliana cells. *Acta physiologiae plantarum / Polish Academy of Sciences, Committee of Plant Physiology Genetics and Breeding*, 39(2):62. https://doi.org/](http://paperpile.com/b/slGjxJ/LoH9B)[10.1007/s11738-017-2365-1](http://dx.doi.org/10.1007/s11738-017-2365-1)

[143. Coppola M, Manco E, Vitiello A, Di Lelio I, Giorgini M, Rao R, Pennacchio F, Digilio MC (2018) Plant response to feeding aphids promotes aphid dispersal. *Entomologia experimentalis et applicata*, 166(5):386–394. https://doi.org/](http://paperpile.com/b/slGjxJ/DBBj1)[10.1111/eea.12677](http://dx.doi.org/10.1111/eea.12677)

[144. Shatters RG Jr, Boykin LM, Lapointe SL, Hunter WB, Weathersbee AA 3rd (2006) Phylogenetic and structural relationships of the PR5 gene family reveal an ancient multigene family conserved in plants and select animal taxa. *Journal of molecular evolution*, 63(1):12–29. https://doi.org/](http://paperpile.com/b/slGjxJ/z4u2X)[10.1007/s00239-005-0053-z](http://dx.doi.org/10.1007/s00239-005-0053-z)

[145. Ditt RF, Kerr KF, Figueiredo P de, Delrow J, Comai L, Nester EW (2006) The Arabidopsis thaliana transcriptome in response to Agrobacterium tumefaciens. *Molecular plant-microbe interactions: MPMI*, 19(6):665–681. https://doi.org/](http://paperpile.com/b/slGjxJ/YGZza)[10.1094/MPMI-19-0665](http://dx.doi.org/10.1094/MPMI-19-0665)

[146. Yamaguchi M, Nagahage ISP, Ohtani M, Ishikawa T, Uchimiya H, Kawai-Yamada M, Demura T (2015) Arabidopsis NAC domain proteins VND-INTERACTING1 and ANAC103 interact with multiple NAC domain proteins. *Plant biotechnology* , 32(2):119–123. https://doi.org/](http://paperpile.com/b/slGjxJ/cuCi5)[10.5511/plantbiotechnology.15.0208a](http://dx.doi.org/10.5511/plantbiotechnology.15.0208a)

[147. Ichino T, Maeda K, Hara-Nishimura I, Shimada T (2020) Arabidopsis ECHIDNA protein is involved in seed coloration, protein trafficking to vacuoles, and vacuolar biogenesis. *Journal of experimental botany*, 71(14):3999–4009. https://doi.org/](http://paperpile.com/b/slGjxJ/gXoRH)[10.1093/jxb/eraa147](http://dx.doi.org/10.1093/jxb/eraa147)

[148. Lee S, Kim J, Kim M-S, Min CW, Kim ST, Choi S-B, Lee JH, Choi D (2021) A Nucleolar Effector of Phytophthora infestans, Pi23226, Binds to rRNA and Subverts Ribosome Biogenesis to Cell Death. https://doi.org/](http://paperpile.com/b/slGjxJ/OlR9C)[10.2139/ssrn.3893906](http://dx.doi.org/10.2139/ssrn.3893906)

[149. Ohbayashi I, Lin C-Y, Shinohara N, Matsumura Y, Machida Y, Horiguchi G, Tsukaya H, Sugiyama M (2017) Evidence for a Role of ANAC082 as a Ribosomal Stress Response Mediator Leading to Growth Defects and Developmental Alterations in Arabidopsis. *The Plant cell*, 29(10):2644–2660. https://doi.org/](http://paperpile.com/b/slGjxJ/Fx65G)[10.1105/tpc.17.00255](http://dx.doi.org/10.1105/tpc.17.00255)

[150. Morreeuw ZP, Escobedo-Fregoso C, Ríos-González LJ, Castillo-Quiroz D, Reyes AG (2021) Transcriptome-based metabolic profiling of flavonoids in Agave lechuguilla waste biomass. *Plant science: an international journal of experimental plant biology*, 305:110748. https://doi.org/](http://paperpile.com/b/slGjxJ/0wISJ)[10.1016/j.plantsci.2020.110748](http://dx.doi.org/10.1016/j.plantsci.2020.110748)

[151. Yamaguchi M, Ohtani M, Mitsuda N, Kubo M, Ohme-Takagi M, Fukuda H, Demura T (2010) VND-INTERACTING2, a NAC domain transcription factor, negatively regulates xylem vessel formation in Arabidopsis. *The Plant cell*, 22(4):1249–1263. https://doi.org/](http://paperpile.com/b/slGjxJ/RLl3Y)[10.1105/tpc.108.064048](http://dx.doi.org/10.1105/tpc.108.064048)

[152. Mousavi SAR (2013) Long-distance wound signalling in arabidopsis.](http://paperpile.com/b/slGjxJ/3lVZc) <https://serval.unil.ch/resource/serval:BIB_5A04D5B19C03.P001/REF.pdf>

[153. Hou S, Wang X, Chen D, Yang X, Wang M, Turrà D, Di Pietro A, Zhang W (2014) The secreted peptide PIP1 amplifies immunity through receptor-like kinase 7. *PLoS pathogens*, 10(9):e1004331. https://doi.org/](http://paperpile.com/b/slGjxJ/pxfGU)[10.1371/journal.ppat.1004331](http://dx.doi.org/10.1371/journal.ppat.1004331)

[154. Shen J, Diao W, Zhang L, Acharya BR, Wang M, Zhao X, Chen D, Zhang W (2020) Secreted Peptide PIP1 Induces Stomatal Closure by Activation of Guard Cell Anion Channels in Arabidopsis. *Frontiers in plant science*, 11:1029. https://doi.org/](http://paperpile.com/b/slGjxJ/YSrra)[10.3389/fpls.2020.01029](http://dx.doi.org/10.3389/fpls.2020.01029)

[155. Jose J, Ghantasala S, Roy Choudhury S (2020) Arabidopsis Transmembrane Receptor-Like Kinases (RLKs): A Bridge between Extracellular Signal and Intracellular Regulatory Machinery. *International journal of molecular sciences*, 21(11)https://doi.org/](http://paperpile.com/b/slGjxJ/k60XP)[10.3390/ijms21114000](http://dx.doi.org/10.3390/ijms21114000)

[156. Tian L, Musetti V, Kim J, Magallanes-Lundback M, DellaPenna D (2004) The Arabidopsis LUT1 locus encodes a member of the cytochrome p450 family that is required for carotenoid epsilon-ring hydroxylation activity. *Proceedings of the National Academy of Sciences of the United States of America*, 101(1):402–407. https://doi.org/](http://paperpile.com/b/slGjxJ/hEzUN)[10.1073/pnas.2237237100](http://dx.doi.org/10.1073/pnas.2237237100)

[157. Kim J-E, Cheng KM, Craft NE, Hamberger B, Douglas CJ (2010) Over-expression of Arabidopsis thaliana carotenoid hydroxylases individually and in combination with a beta-carotene ketolase provides insight into in vivo functions. *Phytochemistry*, 71(2-3):168–178. https://doi.org/](http://paperpile.com/b/slGjxJ/Gn6VK)[10.1016/j.phytochem.2009.10.011](http://dx.doi.org/10.1016/j.phytochem.2009.10.011)

[158. Davies PJ (2004) Plant Hormones: Biosynthesis, Signal Transduction, Action! https://doi.org/](http://paperpile.com/b/slGjxJ/KzJrQ)[10.1007/978-1-4020-2686-7](http://dx.doi.org/10.1007/978-1-4020-2686-7)

[159. Wahlberg, Eklund (1998) Degraded carotenoids. *Carotenoids*,](http://paperpile.com/b/slGjxJ/DTotT)

[160. Felemban A, Braguy J, Zurbriggen MD, Al-Babili S (2019) Apocarotenoids Involved in Plant Development and Stress Response. *Frontiers in plant science*, 10:1168. https://doi.org/](http://paperpile.com/b/slGjxJ/AzY4Z)[10.3389/fpls.2019.01168](http://dx.doi.org/10.3389/fpls.2019.01168)

[161. Heath JJ, Cipollini DF, Stireman JO III (2013) The role of carotenoids and their derivatives in mediating interactions between insects and their environment. *Arthropod-plant interactions*, 7(1):1–20. https://doi.org/](http://paperpile.com/b/slGjxJ/pQFGg)[10.1007/s11829-012-9239-7](http://dx.doi.org/10.1007/s11829-012-9239-7)

[162. Body MJA, Neer WC, Vore C, Lin C-H, Vu DC, Schultz JC, Cocroft RB, Appel HM (2019) Caterpillar Chewing Vibrations Cause Changes in Plant Hormones and Volatile Emissions in Arabidopsis thaliana. *Frontiers in plant science*, 10:810. https://doi.org/](http://paperpile.com/b/slGjxJ/kayRt)[10.3389/fpls.2019.00810](http://dx.doi.org/10.3389/fpls.2019.00810)

[163. Wang S, Ghisalberti EL, Ridsdill-Smith J (1999) Volatiles from Trifolium as feeding deterrents of redlegged earth mites. *Phytochemistry*, 52(4):601–605. https://doi.org/](http://paperpile.com/b/slGjxJ/YStwk)[10.1016/S0031-9422(99)00254-X](http://dx.doi.org/10.1016/S0031-9422(99)00254-X)

[164. Cáceres LA, Lakshminarayan S, Yeung KK-C, McGarvey BD, Hannoufa A, Sumarah MW, Benitez X, Scott IM (2016) Repellent and Attractive Effects of α-, β-, and Dihydro-β- Ionone to Generalist and Specialist Herbivores. *Journal of chemical ecology*, 42(2):107–117. https://doi.org/](http://paperpile.com/b/slGjxJ/wLxkA)[10.1007/s10886-016-0669-z](http://dx.doi.org/10.1007/s10886-016-0669-z)

[165. Gruber MY, Xu N, Grenkow L, Li X, Onyilagha J, Soroka JJ, Westcott ND, Hegedus DD (2009) Responses of the crucifer flea beetle to Brassica volatiles in an olfactometer. *Environmental entomology*, 38(5):1467–1479. https://doi.org/](http://paperpile.com/b/slGjxJ/88lvz)[10.1603/022.038.0515](http://dx.doi.org/10.1603/022.038.0515)

[166. Wei S, Hannoufa A, Soroka J, Xu N, Li X, Zebarjadi A, Gruber M (2011) Enhanced β-ionone emission in Arabidopsis over-expressing AtCCD1 reduces feeding damage in vivo by the crucifer flea beetle. *Environmental entomology*, 40(6):1622–1630. https://doi.org/](http://paperpile.com/b/slGjxJ/eGnr2)[10.1603/EN11088](http://dx.doi.org/10.1603/EN11088)

[167. Ojimelukwe PC, Adler C (1999) Potential of zimtaldehyde, 4-allyl-anisol, linalool, terpineol and other phytochemicals for the control of the confused flour beetle (Tribolium confusum J. d. V.) (Col., Tenebrionidae). *Anzeiger für Schädlingskunde = Journal of pest science*, 72(4):81–86. https://doi.org/](http://paperpile.com/b/slGjxJ/Jmlpf)[10.1007/BF02768913](http://dx.doi.org/10.1007/BF02768913)

[168. Wang W, Hu C, Li X, Zhu Y, Tao L, Cui Y, Deng D, Fan X, Zhang H, Li J, Gou X, Yi J (2022) Receptor-like cytoplasmic kinases PBL34/35/36 are required for CLE peptide-mediated signaling to maintain shoot apical meristem and root apical meristem homeostasis in Arabidopsis. *The Plant Cell*, 34(4):1289–1307. https://doi.org/](http://paperpile.com/b/slGjxJ/ps4eH)[10.1093/plcell/koab315](http://dx.doi.org/10.1093/plcell/koab315)

[169. Luo X, Wu W, Liang Y, Xu N, Wang Z, Zou H, Liu J (2020) Tyrosine phosphorylation of the lectin receptor-like kinase LORE regulates plant immunity. *The EMBO journal*, 39(4):e102856. https://doi.org/](http://paperpile.com/b/slGjxJ/Yv1LM)[10.15252/embj.2019102856](http://dx.doi.org/10.15252/embj.2019102856)

[170. Benedetti M, Verrascina I, Pontiggia D, Locci F, Mattei B, De Lorenzo G, Cervone F (2018) Four Arabidopsis berberine bridge enzyme-like proteins are specific oxidases that inactivate the elicitor-active oligogalacturonides. *The Plant journal: for cell and molecular biology*, 94(2):260–273. https://doi.org/](http://paperpile.com/b/slGjxJ/SwLBh)[10.1111/tpj.13852](http://dx.doi.org/10.1111/tpj.13852)

[171. Voxeur A, Habrylo O, Guénin S, Miart F, Soulié M-C, Rihouey C, Pau-Roblot C, Domon J-M, Gutierrez L, Pelloux J, Mouille G, Fagard M, Höfte H, Vernhettes S (2019) Oligogalacturonide production upon Arabidopsis thaliana-Botrytis cinerea interaction. *Proceedings of the National Academy of Sciences of the United States of America*, 116(39):19743–19752. https://doi.org/](http://paperpile.com/b/slGjxJ/zp38e)[10.1073/pnas.1900317116](http://dx.doi.org/10.1073/pnas.1900317116)

[172. Valsamakis G, Bittner N, Kunze R, Hilker M, Lortzing V (2022) Priming of Arabidopsis resistance to herbivory by insect egg deposition depends on the plant’s developmental stage. *Journal of experimental botany*, 73(14):4996–5015. https://doi.org/](http://paperpile.com/b/slGjxJ/4aAC9)[10.1093/jxb/erac199](http://dx.doi.org/10.1093/jxb/erac199)

[173. Cabrera J, Barcala M, Fenoll C, Escobar C (2016) The Power of Omics to Identify Plant Susceptibility Factors and to Study Resistance to Root-knot Nematodes. *Current issues in molecular biology*, 19:53–72. https://doi.org/](http://paperpile.com/b/slGjxJ/6WzEM)[10.21775/9781910190357.07](http://dx.doi.org/10.21775/9781910190357.07)

[174. Kandoth PK, Ithal N, Recknor J, Maier T, Nettleton D, Baum TJ, Mitchum MG (2011) The Soybean Rhg1 locus for resistance to the soybean cyst nematode Heterodera glycines regulates the expression of a large number of stress- and defense-related genes in degenerating feeding cells. *Plant physiology*, 155(4):1960–1975. https://doi.org/](http://paperpile.com/b/slGjxJ/QBmAU)[10.1104/pp.110.167536](http://dx.doi.org/10.1104/pp.110.167536)

[175. Zolman BK, Monroe-Augustus M, Silva ID, Bartel B (2005) Identification and functional characterization of Arabidopsis PEROXIN4 and the interacting protein PEROXIN22. *The Plant cell*, 17(12):3422–3435. https://doi.org/](http://paperpile.com/b/slGjxJ/Rms8t)[10.1105/tpc.105.035691](http://dx.doi.org/10.1105/tpc.105.035691)

[176. De Rybel B, Audenaert D, Xuan W, Overvoorde P, Strader LC, Kepinski S, Hoye R, Brisbois R, Parizot B, Vanneste S, Liu X, Gilday A, Graham IA, Nguyen L, Jansen L, Njo MF, Inzé D, Bartel B, Beeckman T (2012) A role for the root cap in root branching revealed by the non-auxin probe naxillin. *Nature chemical biology*, 8(9):798–805. https://doi.org/](http://paperpile.com/b/slGjxJ/hmlXr)[10.1038/nchembio.1044](http://dx.doi.org/10.1038/nchembio.1044)

[177. Halitschke R, Gase K, Hui D, Schmidt DD, Baldwin IT (2003) Molecular Interactions between the Specialist Herbivore*Manduca sexta* (Lepidoptera, Sphingidae) and Its Natural Host *Nicotiana attenuata*. VI. Microarray Analysis Reveals That Most Herbivore-Specific Transcriptional Changes Are Mediated by Fatty Acid-Amino Acid Conjugates. *Plant Physiology*, 131(4):1894–1902. https://doi.org/](http://paperpile.com/b/slGjxJ/ziB4O)[10.1104/pp.102.018184](http://dx.doi.org/10.1104/pp.102.018184)

[178. Machado RAR, Robert CAM, Arce CCM, Ferrieri AP, Xu S, Jimenez-Aleman GH, Baldwin IT, Erb M (2016) Auxin Is Rapidly Induced by Herbivore Attack and Regulates a Subset of Systemic, Jasmonate-Dependent Defenses. *Plant physiology*, 172(1):521–532. https://doi.org/](http://paperpile.com/b/slGjxJ/fRW5A)[10.1104/pp.16.00940](http://dx.doi.org/10.1104/pp.16.00940)

[179. Saha P, Ray T, Tang Y, Dutta I, Evangelous NR, Kieliszewski MJ, Chen Y, Cannon MC (2013) Self-rescue of an EXTENSIN mutant reveals alternative gene expression programs and candidate proteins for new cell wall assembly in Arabidopsis. *The Plant journal: for cell and molecular biology*, 75(1):104–116. https://doi.org/](http://paperpile.com/b/slGjxJ/kUaXg)[10.1111/tpj.12204](http://dx.doi.org/10.1111/tpj.12204)

[180. Castilleux R, Plancot B, Ropitaux M, Carreras A, Leprince J, Boulogne I, Follet-Gueye M-L, Popper ZA, Driouich A, Vicré M (2018) Cell wall extensins in root-microbe interactions and root secretions. *Journal of experimental botany*, 69(18):4235–4247. https://doi.org/](http://paperpile.com/b/slGjxJ/3BOrC)[10.1093/jxb/ery238](http://dx.doi.org/10.1093/jxb/ery238)

[181. Kroes A, Broekgaarden C, Castellanos Uribe M, May S, Loon JJA van, Dicke M (2017) Brevicoryne brassicae aphids interfere with transcriptome responses of Arabidopsis thaliana to feeding by Plutella xylostella caterpillars in a density-dependent manner. *Oecologia*, 183(1):107–120. https://doi.org/](http://paperpile.com/b/slGjxJ/H5Y6I)[10.1007/s00442-016-3758-3](http://dx.doi.org/10.1007/s00442-016-3758-3)

[182. Coolen S, Van Pelt JA, Van Wees SCM, Pieterse CMJ (2019) Mining the natural genetic variation in Arabidopsis thaliana for adaptation to sequential abiotic and biotic stresses. *Planta*, 249(4):1087–1105. https://doi.org/](http://paperpile.com/b/slGjxJ/LlnMs)[10.1007/s00425-018-3065-9](http://dx.doi.org/10.1007/s00425-018-3065-9)

[183. Chang H-Y, Cheng T-H, Wang AH-J (2021) Structure, catalysis, and inhibition mechanism of prenyltransferase. *IUBMB life*, 73(1):40–63. https://doi.org/](http://paperpile.com/b/slGjxJ/6NdcR)[10.1002/iub.2418](http://dx.doi.org/10.1002/iub.2418)

[184. Erffelinck M-L, Goossens A (2018) Review: Endoplasmic Reticulum-Associated Degradation (ERAD)-Dependent Control of (Tri)terpenoid Metabolism in Plants. *Planta medica*, 84(12/13):874–880. https://doi.org/](http://paperpile.com/b/slGjxJ/I0lXN)[10.1055/a-0635-8369](http://dx.doi.org/10.1055/a-0635-8369)

[185. Aubert D, Chevillard M, Dorne AM, Arlaud G, Herzog M (1998) Expression patterns of GASA genes in Arabidopsis thaliana: the GASA4 gene is up-regulated by gibberellins in meristematic regions. *Plant molecular biology*, 36(6):871–883. https://doi.org/](http://paperpile.com/b/slGjxJ/WNH31)[10.1023/a:1005938624418](http://dx.doi.org/10.1023/a:1005938624418)

[186. Lee S, Badieyan S, Bevan DR, Herde M, Gatz C, Tholl D (2010) Herbivore-induced and floral homoterpene volatiles are biosynthesized by a single P450 enzyme (CYP82G1) in *Arabidopsis*. *Proceedings of the National Academy of Sciences of the United States of America*, 107(49):21205–21210. https://doi.org/](http://paperpile.com/b/slGjxJ/XpPT7)[10.1073/pnas.1009975107](http://dx.doi.org/10.1073/pnas.1009975107)

[187. Bruce TJA, Matthes MC, Chamberlain K, Woodcock CM, Mohib A, Webster B, Smart LE, Birkett MA, Pickett JA, Napier JA (2008) cis-Jasmone induces Arabidopsis genes that affect the chemical ecology of multitrophic interactions with aphids and their parasitoids. *Proceedings of the National Academy of Sciences of the United States of America*, 105(12):4553–4558. https://doi.org/](http://paperpile.com/b/slGjxJ/f7bTh)[10.1073/pnas.0710305105](http://dx.doi.org/10.1073/pnas.0710305105)

[188. Van Poecke RM, Posthumus MA, Dicke M (2001) Herbivore-induced volatile production by Arabidopsis thaliana leads to attraction of the parasitoid Cotesia rubecula: chemical, behavioral, and gene-expression analysis. *Journal of chemical ecology*, 27(10):1911–1928. https://doi.org/](http://paperpile.com/b/slGjxJ/puWpM)[10.1023/a:1012213116515](http://dx.doi.org/10.1023/a:1012213116515)

[189. Loon JJA, Boer JG, Dicke M (2000) Parasitoid-plant mutualism: parasitoid attack of herbivore increases plant reproduction. *Entomologia experimentalis et applicata*, 97(2):219–227. https://doi.org/](http://paperpile.com/b/slGjxJ/MgpjZ)[10.1046/j.1570-7458.2000.00733.x](http://dx.doi.org/10.1046/j.1570-7458.2000.00733.x)

[190. Arimura G, Ozawa R, Shimoda T, Nishioka T, Boland W, Takabayashi J (2000) Herbivory-induced volatiles elicit defence genes in lima bean leaves. *Nature*, 406(6795):512–515. https://doi.org/](http://paperpile.com/b/slGjxJ/FcbTt)[10.1038/35020072](http://dx.doi.org/10.1038/35020072)

[191. Boer JG de, Posthumus MA, Dicke M (2004) Identification of volatiles that are used in discrimination between plants infested with prey or nonprey herbivores by a predatory mite. *Journal of chemical ecology*, 30(11):2215–2230. https://doi.org/](http://paperpile.com/b/slGjxJ/4Amkl)[10.1023/b:joec.0000048784.79031.5e](http://dx.doi.org/10.1023/b:joec.0000048784.79031.5e)

[192. Mumm R, Posthumus MA, Dicke M (2008) Significance of terpenoids in induced indirect plant defence against herbivorous arthropods. *Plant, cell & environment*, 31(4):575–585. https://doi.org/](http://paperpile.com/b/slGjxJ/wBTU4)[10.1111/j.1365-3040.2008.01783.x](http://dx.doi.org/10.1111/j.1365-3040.2008.01783.x)

[193. Qin S, Fan C, Li X, Li Y, Hu J, Li C, Luo K (2020) LACCASE14 is required for the deposition of guaiacyl lignin and affects cell wall digestibility in poplar. *Biotechnology for biofuels*, 13(1):197. https://doi.org/](http://paperpile.com/b/slGjxJ/pa81L)[10.1186/s13068-020-01843-4](http://dx.doi.org/10.1186/s13068-020-01843-4)

[194. Kumar P, Khanal S, Da Silva M, Singh R, Davis RF, Nichols RL, Chee PW (2019) Transcriptome analysis of a nematode resistant and susceptible upland cotton line at two critical stages of Meloidogyne incognita infection and development. *PloS one*, 14(9):e0221328. https://doi.org/](http://paperpile.com/b/slGjxJ/obt9A)[10.1371/journal.pone.0221328](http://dx.doi.org/10.1371/journal.pone.0221328)

[195. Abdul Malik NA, Kumar IS, Nadarajah K (2020) Elicitor and Receptor Molecules: Orchestrators of Plant Defense and Immunity. *International journal of molecular sciences*, 21(3)https://doi.org/](http://paperpile.com/b/slGjxJ/XkDmT)[10.3390/ijms21030963](http://dx.doi.org/10.3390/ijms21030963)

[196. Gouhier-Darimont C, Stahl E, Glauser G, Reymond P (2019) The Arabidopsis Lectin Receptor Kinase LecRK-I.8 Is Involved in Insect Egg Perception. *Frontiers in plant science*, 10:623. https://doi.org/](http://paperpile.com/b/slGjxJ/pkM71)[10.3389/fpls.2019.00623](http://dx.doi.org/10.3389/fpls.2019.00623)

[197. Oates CN, Denby KJ, Myburg AA, Slippers B, Naidoo S (2021) Insect egg-induced physiological changes and transcriptional reprogramming leading to gall formation. *Plant, cell & environment*, 44(2):535–547. https://doi.org/](http://paperpile.com/b/slGjxJ/6Lmnh)[10.1111/pce.13930](http://dx.doi.org/10.1111/pce.13930)

[198. Oates CN, Denby KJ, Myburg AA, Slippers B, Naidoo S (2016) Insect Gallers and Their Plant Hosts: From Omics Data to Systems Biology. *International journal of molecular sciences*, 17(11)https://doi.org/](http://paperpile.com/b/slGjxJ/j9SYt)[10.3390/ijms17111891](http://dx.doi.org/10.3390/ijms17111891)

[199. (2010) The regulation of host translation initiation in plant-pathogen interactions.](http://paperpile.com/b/slGjxJ/dkhu1) <https://search.proquest.com/openview/201b2d076e540c2b57f2df69817d2527/1?pq-origsite=gscholar&cbl=18750>

[200. (2005) Molecular insights into Arabidopsis response to Myzus persicae Sulzer (green peach aphid).](http://paperpile.com/b/slGjxJ/UM6V7) <https://search.proquest.com/openview/1ac9497fd676562bade6d669ea5675fc/1?pq-origsite=gscholar&cbl=18750&diss=y>

[201. Mendy B, Wang’ombe MW, Radakovic ZS, Holbein J, Ilyas M, Chopra D, Holton N, Zipfel C, Grundler FMW, Siddique S (2017) Arabidopsis leucine-rich repeat receptor-like kinase NILR1 is required for induction of innate immunity to parasitic nematodes. *PLoS pathogens*, 13(4):e1006284. https://doi.org/](http://paperpile.com/b/slGjxJ/jlUJE)[10.1371/journal.ppat.1006284](http://dx.doi.org/10.1371/journal.ppat.1006284)

[202. Bassetti N, Caarls L, Bukovinszkine’Kiss G, El-Soda M, Veen J van, Bouwmeester K, Zwaan BJ, Schranz ME, Bonnema G, Fatouros NE (2022) Genetic analysis reveals three novel QTLs underpinning a butterfly egg-induced hypersensitive response-like cell death in Brassica rapa. *BMC plant biology*, 22(1):140. https://doi.org/](http://paperpile.com/b/slGjxJ/m1Oou)[10.1186/s12870-022-03522-y](http://dx.doi.org/10.1186/s12870-022-03522-y)

[203. Yuan M, Jiang Z, Bi G, Nomura K, Liu M, Wang Y, Cai B, Zhou J-M, He SY, Xin X-F (2021) Pattern-recognition receptors are required for NLR-mediated plant immunity. *Nature*, 592(7852):105–109. https://doi.org/](http://paperpile.com/b/slGjxJ/vayWU)[10.1038/s41586-021-03316-6](http://dx.doi.org/10.1038/s41586-021-03316-6)

[204. Ngou BPM, Ahn H-K, Ding P, Jones JDG (2021) Mutual potentiation of plant immunity by cell-surface and intracellular receptors. *Nature*, 592(7852):110–115. https://doi.org/](http://paperpile.com/b/slGjxJ/xoGO3)[10.1038/s41586-021-03315-7](http://dx.doi.org/10.1038/s41586-021-03315-7)

[205. Jung HW, Panigrahi GK, Jung GY, Lee YJ, Shin KH, Sahoo A, Choi ES, Lee E, Man Kim K, Yang SH, Jeon J-S, Lee SC, Kim SH (2020) Pathogen-Associated Molecular Pattern-Triggered Immunity Involves Proteolytic Degradation of Core Nonsense-Mediated mRNA Decay Factors During the Early Defense Response. *The Plant cell*, 32(4):1081–1101. https://doi.org/](http://paperpile.com/b/slGjxJ/r7wdR)[10.1105/tpc.19.00631](http://dx.doi.org/10.1105/tpc.19.00631)

[206. Edreva A, Kostoff D (2005) Pathogenesis-related proteins: Research progress in the last 15 years.](http://paperpile.com/b/slGjxJ/oFw6W) <http://www.bio21.bas.bg/ipp/gapbfiles/v-31/05_1-2_105-124.pdf?origin>

[207. Van Loon LC (1997) Induced resistance in plants and the role of pathogenesis-related proteins. *European journal of plant pathology / European Foundation for Plant Pathology*, 103(9):753–765. https://doi.org/](http://paperpile.com/b/slGjxJ/s1SYB)[10.1023/A:1008638109140](http://dx.doi.org/10.1023/A:1008638109140)

[208. Stintzi A, Heitz T, Prasad V, Wiedemann-Merdinoglu S, Kauffmann S, Geoffroy P, Legrand M, Fritig B (1993) Plant “pathogenesis-related” proteins and their role in defense against pathogens. *Biochimie*, 75(8):687–706. https://doi.org/](http://paperpile.com/b/slGjxJ/uFZgd)[10.1016/0300-9084(93)90100-7](http://dx.doi.org/10.1016/0300-9084(93)90100-7)

[209. Wan J, He M, Hou Q, Zou L, Yang Y, Wei Y, Chen X (2021) Cell wall associated immunity in plants. *Stress Biology*, 1(1):3. https://doi.org/](http://paperpile.com/b/slGjxJ/zDWoB)[10.1007/s44154-021-00003-4](http://dx.doi.org/10.1007/s44154-021-00003-4)

[210. Bacete L, Mélida H, Miedes E, Molina A (2018) Plant cell wall-mediated immunity: cell wall changes trigger disease resistance responses. *The Plant journal: for cell and molecular biology*, 93(4):614–636. https://doi.org/](http://paperpile.com/b/slGjxJ/d7AlG)[10.1111/tpj.13807](http://dx.doi.org/10.1111/tpj.13807)

[211. Hoffmann N, Benske A, Betz H, Schuetz M, Samuels AL (2020) Laccases and Peroxidases Co-Localize in Lignified Secondary Cell Walls throughout Stem Development. *Plant physiology*, 184(2):806–822. https://doi.org/](http://paperpile.com/b/slGjxJ/BSi3K)[10.1104/pp.20.00473](http://dx.doi.org/10.1104/pp.20.00473)

[212. Wuyts N, Lognay G, Swennen R, De Waele D (2006) Nematode infection and reproduction in transgenic and mutant Arabidopsis and tobacco with an altered phenylpropanoid metabolism. *Journal of experimental botany*, 57(11):2825–2835. https://doi.org/](http://paperpile.com/b/slGjxJ/XoCjy)[10.1093/jxb/erl044](http://dx.doi.org/10.1093/jxb/erl044)

[213. Harris MO, Freeman TP, Rohfritsch O, Anderson KG, Payne SA, Moore JA (2006) Virulent Hessian Fly (Diptera: Cecidomyiidae) Larvae Induce a Nutritive Tissue During Compatible Interactions with Wheat. *Annals of the Entomological Society of America*, 99(2):305–316. https://doi.org/](http://paperpile.com/b/slGjxJ/pb9Lk)[10.1603/0013-8746(2006)099[0305:VHFDCL]2.0.CO;2](http://dx.doi.org/10.1603/0013-8746(2006)099%5B0305:VHFDCL%5D2.0.CO;2)

[214. Harris MO, Freeman TP, Moore JA, Anderson KG, Payne SA, Anderson KM, Rohfritsch O (2010) H-gene-mediated resistance to Hessian fly exhibits features of penetration resistance to fungi. *Phytopathology*, 100(3):279–289. https://doi.org/](http://paperpile.com/b/slGjxJ/5QBja)[10.1094/PHYTO-100-3-0279](http://dx.doi.org/10.1094/PHYTO-100-3-0279)

[215. Tilsner J, Nicolas W, Rosado A, Bayer EM (2016) Staying Tight: Plasmodesmal Membrane Contact Sites and the Control of Cell-to-Cell Connectivity in Plants. *Annual review of plant biology*, 67:337–364. https://doi.org/](http://paperpile.com/b/slGjxJ/o1XgH)[10.1146/annurev-arplant-043015-111840](http://dx.doi.org/10.1146/annurev-arplant-043015-111840)

[216. Baluska F, Samaj J, Napier R, Volkmann D (1999) Maize calreticulin localizes preferentially to plasmodesmata in root apex. *The Plant journal: for cell and molecular biology*, 19(4):481–488. https://doi.org/](http://paperpile.com/b/slGjxJ/6ieP3)[10.1046/j.1365-313x.1999.00530.x](http://dx.doi.org/10.1046/j.1365-313x.1999.00530.x)

[217. Lee J-Y, Lu H (2011) Plasmodesmata: the battleground against intruders. *Trends in plant science*, 16(4):201–210. https://doi.org/](http://paperpile.com/b/slGjxJ/GDUOs)[10.1016/j.tplants.2011.01.004](http://dx.doi.org/10.1016/j.tplants.2011.01.004)

[218. Cheval C, Faulkner C (2018) Plasmodesmal regulation during plant-pathogen interactions. *The New phytologist*, 217(1):62–67. https://doi.org/](http://paperpile.com/b/slGjxJ/RFbRJ)[10.1111/nph.14857](http://dx.doi.org/10.1111/nph.14857)

[219. Liu J, Zhang L, Yan D (2021) Plasmodesmata-Involved Battle Against Pathogens and Potential Strategies for Strengthening Hosts. *Frontiers in plant science*, 12:644870. https://doi.org/](http://paperpile.com/b/slGjxJ/PIIvh)[10.3389/fpls.2021.644870](http://dx.doi.org/10.3389/fpls.2021.644870)

[220. Wang Y, Li X, Fan B, Zhu C, Chen Z (2021) Regulation and Function of Defense-Related Callose Deposition in Plants. *International journal of molecular sciences*, 22(5)https://doi.org/](http://paperpile.com/b/slGjxJ/G9Gl1)[10.3390/ijms22052393](http://dx.doi.org/10.3390/ijms22052393)

[221. Hao P, Liu C, Wang Y, Chen R, Tang M, Du B (2008) Herbivore-induced callose deposition on the sieve plates of rice: an important mechanism for host resistance. *Plant*,](http://paperpile.com/b/slGjxJ/t5cAk) <https://academic.oup.com/plphys/article-abstract/146/4/1810/6107251>

[222. Xu L-H, Xiao L-Y, Xiao Y-N, Peng D-L, Xiao X-Q, Huang W-K, Gheysen G, Wang G-F (2021) Plasmodesmata play pivotal role in sucrose supply to Meloidogyne graminicola-caused giant cells in rice. *Molecular plant pathology*, 22(5):539–550. https://doi.org/](http://paperpile.com/b/slGjxJ/zwuiQ)[10.1111/mpp.13042](http://dx.doi.org/10.1111/mpp.13042)

[223. Qaseem MF, Wu A-M (2020) Balanced Xylan Acetylation is the Key Regulator of Plant Growth and Development, and Cell Wall Structure and for Industrial Utilization. *International journal of molecular sciences*, 21(21)https://doi.org/](http://paperpile.com/b/slGjxJ/8qFjU)[10.3390/ijms21217875](http://dx.doi.org/10.3390/ijms21217875)

[224. Bhuiyan NH, Selvaraj G, Wei Y, King J (2009) Role of lignification in plant defense. *Plant signaling & behavior*, 4(2):158–159. https://doi.org/](http://paperpile.com/b/slGjxJ/KYVVQ)[10.4161/psb.4.2.7688](http://dx.doi.org/10.4161/psb.4.2.7688)

[225. Schultz JC, Edger PP, Body MJA, Appel HM (2019) A galling insect activates plant reproductive programs during gall development. *Scientific reports*, 9(1):1833. https://doi.org/](http://paperpile.com/b/slGjxJ/ZmAbo)[10.1038/s41598-018-38475-6](http://dx.doi.org/10.1038/s41598-018-38475-6)

[226. Kudla U (2006) The role of cell wall-modifying proteins in plant penetration and feeding site proliferation by the potato cyst nematode Globodera rostochiensis.](http://paperpile.com/b/slGjxJ/oLyZB) <https://library.wur.nl/WebQuery/wurpubs/345881>

[227. Brummell DA, Harpster MH (2001) Cell wall metabolism in fruit softening and quality and its manipulation in transgenic plants. *Plant molecular biology*, 47(1-2):311–340.](http://paperpile.com/b/slGjxJ/c97mZ) <https://www.ncbi.nlm.nih.gov/pubmed/11554479>

[228. Marín‐Rodríguez MC, Orchard J, Seymour GB (2002) Pectate lyases, cell wall degradation and fruit softening. *Journal of experimental botany*, 53(377):2115–2119. https://doi.org/](http://paperpile.com/b/slGjxJ/sxrWc)[10.1093/jxb/erf089](http://dx.doi.org/10.1093/jxb/erf089)

[229. Catalá C, Rose JK, Bennett AB (2000) Auxin-regulated genes encoding cell wall-modifying proteins are expressed during early tomato fruit growth. *Plant physiology*, 122(2):527–534. https://doi.org/](http://paperpile.com/b/slGjxJ/oT6Ym)[10.1104/pp.122.2.527](http://dx.doi.org/10.1104/pp.122.2.527)

[230. Fudali S, Sobczak M, Janakowski S, Griesser M, Grundler FM, Golinowski W (2008) Expansins are among plant cell wall modifying agents specifically expressed during development of nematode-induced syncytia. *Plant signaling & behavior*, 3(11):969–971. https://doi.org/](http://paperpile.com/b/slGjxJ/UWfym)[10.4161/psb.6169](http://dx.doi.org/10.4161/psb.6169)

[231. Kuluev BR, Knyazev AB, Lebedev YP, Chemeris AV (2012) Morphological and physiological characteristics of transgenic tobacco plants expressing expansin genes: AtEXP10 from Arabidopsis and PnEXPA1 from poplar. *Russian journal of plant physiology: a comprehensive Russian journal on modern phytophysiology*, 59(1):97–104. https://doi.org/](http://paperpile.com/b/slGjxJ/h7Tql)[10.1134/S1021443712010128](http://dx.doi.org/10.1134/S1021443712010128)

[232. Boachon B, Gamir J, Pastor V, Erb M, Dean JV, Flors V, Mauch-Mani B (2014) Role of two UDP-Glycosyltransferases from the L group of arabidopsis in resistance against pseudomonas syringae. *European journal of plant pathology / European Foundation for Plant Pathology*, 139(4):707–720. https://doi.org/](http://paperpile.com/b/slGjxJ/xs2fD)[10.1007/s10658-014-0424-7](http://dx.doi.org/10.1007/s10658-014-0424-7)

[233. Koo YJ, Kim MA, Kim EH, Song JT, Jung C, Moon J-K, Kim J-H, Seo HS, Song SI, Kim J-K, Lee JS, Cheong J-J, Choi YD (2007) Overexpression of salicylic acid carboxyl methyltransferase reduces salicylic acid-mediated pathogen resistance in Arabidopsis thaliana. *Plant molecular biology*, 64(1-2):1–15. https://doi.org/](http://paperpile.com/b/slGjxJ/AP19t)[10.1007/s11103-006-9123-x](http://dx.doi.org/10.1007/s11103-006-9123-x)

[234. Jasrotia S, Jasrotia R (2022) Role of ethylene in combating biotic stress. *Ethylene in Plant Biology*, :388–397. https://doi.org/](http://paperpile.com/b/slGjxJ/dSDgP)[10.1002/9781119744719.ch18](http://dx.doi.org/10.1002/9781119744719.ch18)

[235. Abdelsamad NA, MacIntosh GC, Leandro LFS (2019) Induction of ethylene inhibits development of soybean sudden death syndrome by inducing defense-related genes and reducing Fusarium virguliforme growth. *PloS one*, 14(5):e0215653. https://doi.org/](http://paperpile.com/b/slGjxJ/ypnJM)[10.1371/journal.pone.0215653](http://dx.doi.org/10.1371/journal.pone.0215653)

[236. Pieterse CMJ, Zamioudis C, Berendsen RL, Weller DM, Van Wees SCM, Bakker PAHM (2014) Induced systemic resistance by beneficial microbes. *Annual review of phytopathology*, 52:347–375. https://doi.org/](http://paperpile.com/b/slGjxJ/6koMt)[10.1146/annurev-phyto-082712-102340](http://dx.doi.org/10.1146/annurev-phyto-082712-102340)

[237. Ciardi JA, Tieman DM, Lund ST, Jones JB, Stall RE, Klee HJ (2000) Response to Xanthomonas campestris pv. vesicatoria in tomato involves regulation of ethylene receptor gene expression. *Plant physiology*, 123(1):81–92. https://doi.org/](http://paperpile.com/b/slGjxJ/jH0Ys)[10.1104/pp.123.1.81](http://dx.doi.org/10.1104/pp.123.1.81)

[238. Robison MM, Griffith M, Pauls KP, Glick BR (2008) Dual role for ethylene in susceptibility of tomato to Verticillium wilt. *Phytopathologische Zeitschrift. Journal of phytopathology*, 149(7-8):385–388. https://doi.org/](http://paperpile.com/b/slGjxJ/ZZOOm)[10.1111/j.1439-0434.2001.tb03867.x](http://dx.doi.org/10.1111/j.1439-0434.2001.tb03867.x)

[239. Rubinstein B (2000) Regulation of cell death in flower petals. *Plant molecular biology*, 44(3):303–318. https://doi.org/](http://paperpile.com/b/slGjxJ/41aW0)[10.1023/a:1026540524990](http://dx.doi.org/10.1023/a:1026540524990)

[240. Perrot-Rechenmann C (2010) Cellular responses to auxin: division versus expansion. *Cold Spring Harbor perspectives in biology*, 2(5):a001446. https://doi.org/](http://paperpile.com/b/slGjxJ/AMj8F)[10.1101/cshperspect.a001446](http://dx.doi.org/10.1101/cshperspect.a001446)

[241. Zimmerman PW (1935) Several chemical growth substances which cause initiation of roots and other responses in plants. *Contrib. Boyce Thompson Inst.*, 7:209–229.](http://paperpile.com/b/slGjxJ/l6PQB) <https://ci.nii.ac.jp/naid/10007774402/>

[242. Davies PJ (2010) The Plant Hormones: Their Nature, Occurrence, and Functions. *Plant Hormones: Biosynthesis, Signal Transduction, Action!*, :1–15. https://doi.org/](http://paperpile.com/b/slGjxJ/AUk8D)[10.1007/978-1-4020-2686-7_1](http://dx.doi.org/10.1007/978-1-4020-2686-7_1)

[243. Perrot‐Rechenmann C, Napier RM (2005) Auxins. *Vitamins & Hormones*, 72:203–233. https://doi.org/](http://paperpile.com/b/slGjxJ/UIGmn)[10.1016/S0083-6729(04)72006-3](http://dx.doi.org/10.1016/S0083-6729(04)72006-3)

[244. Wei X, Wei X, Guan W, Mao L (2021) Abscisic acid stimulates wound suberisation in kiwifruit (Actinidia chinensis) by regulating the production of jasmonic acid, cytokinin and auxin. *Functional plant biology: FPB*, 48(11):1100–1112. https://doi.org/](http://paperpile.com/b/slGjxJ/fqLq7)[10.1071/FP20360](http://dx.doi.org/10.1071/FP20360)

[245. Zolman BK, Martinez N, Millius A, Adham AR, Bartel B (2008) Identification and characterization of Arabidopsis indole-3-butyric acid response mutants defective in novel peroxisomal enzymes. *Genetics*, 180(1):237–251. https://doi.org/](http://paperpile.com/b/slGjxJ/OtRj7)[10.1534/genetics.108.090399](http://dx.doi.org/10.1534/genetics.108.090399)

[246. Ludwig-Müller J (2000) Indole-3-butyric acid in plant growth and development. *Plant growth regulation*, 32(2):219–230. https://doi.org/](http://paperpile.com/b/slGjxJ/MGRPh)[10.1023/A:1010746806891](http://dx.doi.org/10.1023/A:1010746806891)

[247. Takei K, Yamaya T, Sakakibara H (2004) Arabidopsis CYP735A1 and CYP735A2 encode cytokinin hydroxylases that catalyze the biosynthesis of trans-Zeatin. *The Journal of biological chemistry*, 279(40):41866–41872. https://doi.org/](http://paperpile.com/b/slGjxJ/qq7Co)[10.1074/jbc.M406337200](http://dx.doi.org/10.1074/jbc.M406337200)

[248. Kieber JJ, Schaller GE (2014) Cytokinins. *The Arabidopsis book / American Society of Plant Biologists*, 12:e0168. https://doi.org/](http://paperpile.com/b/slGjxJ/vcEQ7)[10.1199/tab.0168](http://dx.doi.org/10.1199/tab.0168)

[249. Mitchell JJ, Staden J van (1983) Cytokinins and the Wounding Response in Potato Tissue. *Zeitschrift für Pflanzenphysiologie*, 109(1):1–5. https://doi.org/](http://paperpile.com/b/slGjxJ/XFTaP)[10.1016/S0044-328X(83)80167-6](http://dx.doi.org/10.1016/S0044-328X(83)80167-6)

[250. Crane KE, Ross CW (1986) Effects of wounding on cytokinin activity in cucumber cotyledons. *Plant physiology*, 82(4):1151–1152. https://doi.org/](http://paperpile.com/b/slGjxJ/oFfUZ)[10.1104/pp.82.4.1151](http://dx.doi.org/10.1104/pp.82.4.1151)

[251. Dervinis C, Frost CJ, Lawrence SD, Novak NG, Davis JM (2010) Cytokinin primes plant responses to wounding and reduces insect performance. *Journal of plant growth regulation*, 29(3):289–296. https://doi.org/](http://paperpile.com/b/slGjxJ/i66NJ)[10.1007/s00344-009-9135-2](http://dx.doi.org/10.1007/s00344-009-9135-2)

[252. Hui D, Iqbal J, Lehmann K, Gase K, Saluz HP, Baldwin IT (2003) Molecular interactions between the specialist herbivore Manduca sexta (Lepidoptera, Sphingidae) and its natural host Nicotiana attenuata: V. Microarray analysis and further characterization of large-scale changes in herbivore-induced mRNAs. *Plant physiology*, 131(4):1877–1893.](http://paperpile.com/b/slGjxJ/fBFmG) <https://academic.oup.com/plphys/article-abstract/131/4/1877/6103049>

[253. Babosha AV (2009) Regulation of resistance and susceptibility in wheat–powdery mildew pathosystem with exogenous cytokinins. *Journal of plant physiology*, 166(17):1892–1903. https://doi.org/](http://paperpile.com/b/slGjxJ/6M1n5)[10.1016/j.jplph.2009.05.014](http://dx.doi.org/10.1016/j.jplph.2009.05.014)

[254. Akagi A, Fukushima S, Okada K, Jiang C-J, Yoshida R, Nakayama A, Shimono M, Sugano S, Yamane H, Takatsuji H (2014) WRKY45-dependent priming of diterpenoid phytoalexin biosynthesis in rice and the role of cytokinin in triggering the reaction. *Plant molecular biology*, 86(1-2):171–183. https://doi.org/](http://paperpile.com/b/slGjxJ/3iMF0)[10.1007/s11103-014-0221-x](http://dx.doi.org/10.1007/s11103-014-0221-x)

[255. Jiang C-J, Shimono M, Sugano S, Kojima M, Liu X, Inoue H, Sakakibara H, Takatsuji H (2013) Cytokinins act synergistically with salicylic acid to activate defense gene expression in rice. *Molecular plant-microbe interactions: MPMI*, 26(3):287–296. https://doi.org/](http://paperpile.com/b/slGjxJ/68Rj1)[10.1094/MPMI-06-12-0152-R](http://dx.doi.org/10.1094/MPMI-06-12-0152-R)

[256. Großkinsky DK, Tafner R, Moreno MV, Stenglein SA, Salamone IEG de, Nelson LM, Novák O, Strnad M, Graaff E van der, Roitsch T (2016) Cytokinin production by Pseudomonas fluorescens G20-18 determines biocontrol activity against Pseudomonas syringae in Arabidopsis. *Scientific Reports*, 6(1)https://doi.org/](http://paperpile.com/b/slGjxJ/VxxKQ)[10.1038/srep23310](http://dx.doi.org/10.1038/srep23310)

[257. Choi J, Huh SU, Kojima M, Sakakibara H, Paek K-H, Hwang I (2010) The cytokinin-activated transcription factor ARR2 promotes plant immunity via TGA3/NPR1-dependent salicylic acid signaling in Arabidopsis. *Developmental cell*, 19(2):284–295. https://doi.org/](http://paperpile.com/b/slGjxJ/UUR3e)[10.1016/j.devcel.2010.07.011](http://dx.doi.org/10.1016/j.devcel.2010.07.011)

[258. Grosskinsky DK, Naseem M, Abdelmohsen UR, Plickert N, Engelke T, Griebel T, Zeier J, Novák O, Strnad M, Pfeifhofer H, Graaff E van der, Simon U, Roitsch T (2011) Cytokinins mediate resistance against Pseudomonas syringae in tobacco through increased antimicrobial phytoalexin synthesis independent of salicylic acid signaling. *Plant physiology*, 157(2):815–830. https://doi.org/](http://paperpile.com/b/slGjxJ/AfvCV)[10.1104/pp.111.182931](http://dx.doi.org/10.1104/pp.111.182931)

[259. Pertry I, Václavíková K, Depuydt S, Galuszka P, Spíchal L, Temmerman W, Stes E, Schmülling T, Kakimoto T, Van Montagu MCE, Strnad M, Holsters M, Tarkowski P, Vereecke D (2009) Identification of *Rhodococcus fascians* cytokinins and their modus operandi to reshape the plant. *Proceedings of the National Academy of Sciences of the United States of America*, 106(3):929–934. https://doi.org/](http://paperpile.com/b/slGjxJ/NNhnu)[10.1073/pnas.0811683106](http://dx.doi.org/10.1073/pnas.0811683106)

[260. Yokota T (1999) Chapter 12 - Brassinosteroids. *New Comprehensive Biochemistry*, 33:277–293. https://doi.org/](http://paperpile.com/b/slGjxJ/OX3va)[10.1016/S0167-7306(08)60492-5](http://dx.doi.org/10.1016/S0167-7306(08)60492-5)

[261. Khan EA, Upadhyay TK, Prajapat RK, Mathur M (2022) Chapter 2 - Revisiting brassinosteroids signaling in plants: current advances and challenges. *Brassinosteroids in Plant Developmental Biology and Stress Tolerance*, :15–41. https://doi.org/](http://paperpile.com/b/slGjxJ/5L10C)[10.1016/B978-0-12-813227-2.00010-2](http://dx.doi.org/10.1016/B978-0-12-813227-2.00010-2)

[262. Divi UK, Krishna P (2009) Brassinosteroid: a biotechnological target for enhancing crop yield and stress tolerance. *New biotechnology*, 26(3-4):131–136. https://doi.org/](http://paperpile.com/b/slGjxJ/tGIms)[10.1016/j.nbt.2009.07.006](http://dx.doi.org/10.1016/j.nbt.2009.07.006)

[263. Krishna P (2003) Brassinosteroid-Mediated Stress Responses. *Journal of plant growth regulation*, 22(4):289–297. https://doi.org/](http://paperpile.com/b/slGjxJ/D5BaQ)[10.1007/s00344-003-0058-z](http://dx.doi.org/10.1007/s00344-003-0058-z)

[264. Clouse SD, Sasse JM (1998) BRASSINOSTEROIDS: Essential Regulators of Plant Growth and Development. *Annual review of plant physiology and plant molecular biology*, 49:427–451. https://doi.org/](http://paperpile.com/b/slGjxJ/bobXc)[10.1146/annurev.arplant.49.1.427](http://dx.doi.org/10.1146/annurev.arplant.49.1.427)

[265. Duffey SS, Stout MJ (1996) Antinutritive and toxic components of plant defense against insects. *Archives of insect biochemistry and physiology*, 32(1):3–37. https://doi.org/](http://paperpile.com/b/slGjxJ/xOGJy)[10.1002/(sici)1520-6327(1996)32:1<3::aid-arch2>3.0.co;2-1](http://dx.doi.org/10.1002/(sici)1520-6327(1996)32:1%3C3::aid-arch2%3E3.0.co;2-1)

[266. Kost C, Heil M (2006) Herbivore-induced plant volatiles induce an indirect defence in neighbouring plants. *Journal of Ecology*, 94(3):619–628. https://doi.org/](http://paperpile.com/b/slGjxJ/HALUs)[10.1111/j.1365-2745.2006.01120.x](http://dx.doi.org/10.1111/j.1365-2745.2006.01120.x)

[267. Matthes MC, Bruce TJA, Ton J, Verrier PJ, Pickett JA, Napier JA (2010) The transcriptome of cis-jasmone-induced resistance in Arabidopsis thaliana and its role in indirect defence. *Planta*, 232(5):1163–1180. https://doi.org/](http://paperpile.com/b/slGjxJ/PKzYD)[10.1007/s00425-010-1244-4](http://dx.doi.org/10.1007/s00425-010-1244-4)

[268. Birkett MA, Campbell CA, Chamberlain K, Guerrieri E, Hick AJ, Martin JL, Matthes M, Napier JA, Pettersson J, Pickett JA, Poppy GM, Pow EM, Pye BJ, Smart LE, Wadhams GH, Wadhams LJ, Woodcock CM (2000) New roles for cis-jasmone as an insect semiochemical and in plant defense. *Proceedings of the National Academy of Sciences of the United States of America*, 97(16):9329–9334. https://doi.org/](http://paperpile.com/b/slGjxJ/Wpuoo)[10.1073/pnas.160241697](http://dx.doi.org/10.1073/pnas.160241697)

[269. Shorthouse JD, West A, Landry RW, Thibodeau PD (1986) STRUCTURAL DAMAGE BY FEMALE HEMADAS NUBILIPENNIS (HYMENOPTERA: PTEROMALIDAE) AS A FACTOR IN GALL INDUCTION ON LOWBUSH BLUEBERRY. *The Canadian entomologist*, 118(3):249–254. https://doi.org/](http://paperpile.com/b/slGjxJ/JnY9q)[10.4039/Ent118249-3](http://dx.doi.org/10.4039/Ent118249-3)

[270. West A, Shorthouse JD (1989) Initiation and development of the stem gall induced by Hemadas nubilipennis (Hymenoptera: Pteromalidae) on lowbush blueberry, Vaccinium angustifolium (Ericaceae). *Canadian journal of botany. Journal canadien de botanique*, 67(7):2187–2198. https://doi.org/](http://paperpile.com/b/slGjxJ/8cgd7)[10.1139/b89-278](http://dx.doi.org/10.1139/b89-278)

[271. Poirié M, Colinet D, Gatti J-L (2014) Insights into function and evolution of parasitoid wasp venoms. *Current opinion in insect science*, 6:52–60. https://doi.org/](http://paperpile.com/b/slGjxJ/K9Tgp)[10.1016/j.cois.2014.10.004](http://dx.doi.org/10.1016/j.cois.2014.10.004)

[272. Cambier S, Ginis O, Moreau SJM, Gayral P, Hearn J, Stone GN, Giron D, Huguet E, Drezen J-M (2019) Gall Wasp Transcriptomes Unravel Potential Effectors Involved in Molecular Dialogues With Oak and Rose. *Frontiers in physiology*, 10:926. https://doi.org/](http://paperpile.com/b/slGjxJ/jsheO)[10.3389/fphys.2019.00926](http://dx.doi.org/10.3389/fphys.2019.00926)
