# Supplementary material for: Deciphering the underlying genetics of galling resistance to the blueberry stem gall wasp in northern highbush blueberry
Source: Hortic Res. 2025 Jul 29;12(11):uhaf197. doi: 10.1093/hr/uhaf197 (PMC12552771; doi:10.1093/hr/uhaf197)

**Supplemental Figure S2.** Allelic breakdown of plants (percent of plants having each combination) found to be resistant and susceptible to gall-inducing wasps at each of the four loci.


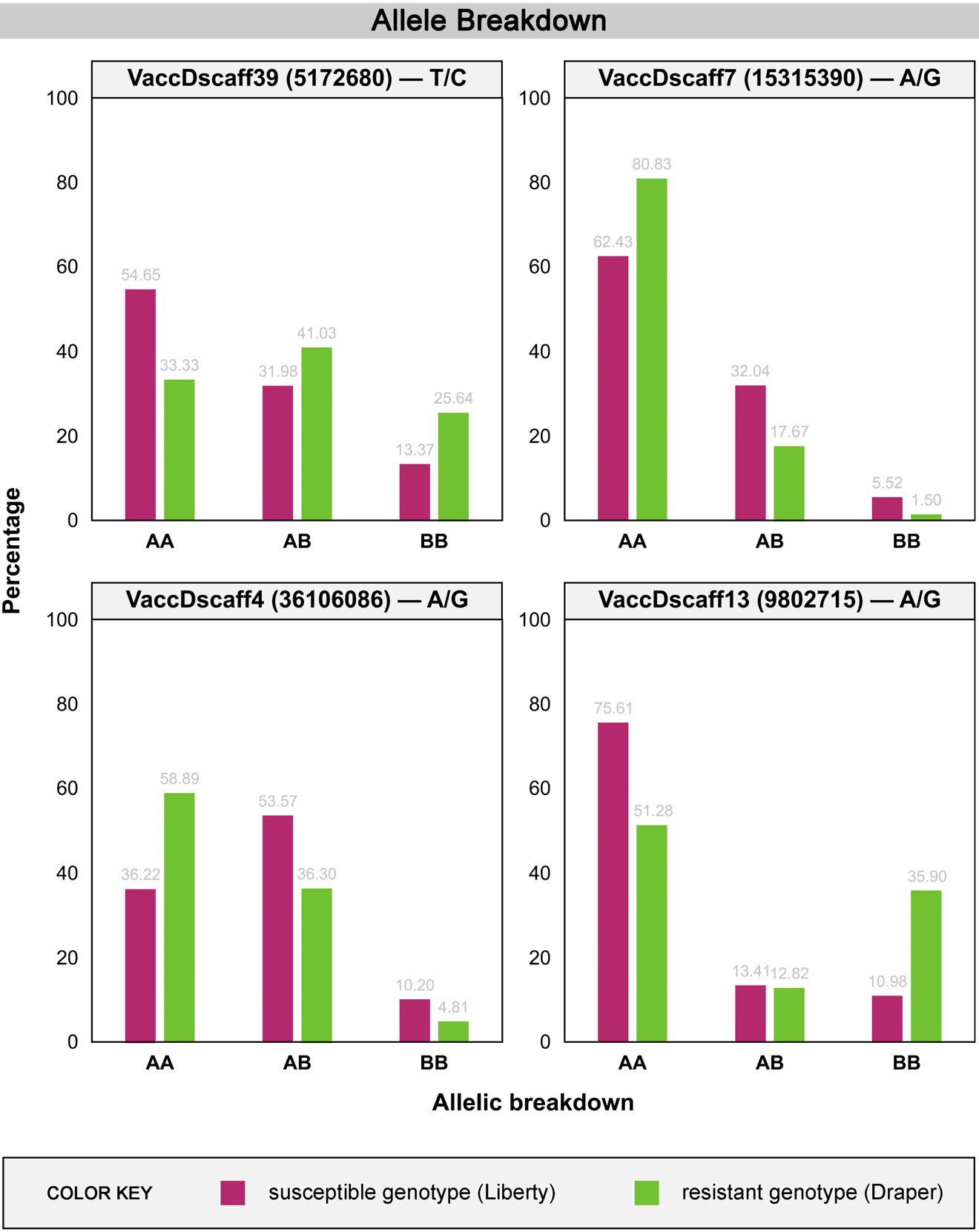

Supplement: Web_Material_uhaf197 [file web_material_uhaf197.zip › Supplemental Figure S1 - Allelic breakdown.docx]
